# Generation of a chromosome-scale genome assembly of the insect-repellent terpenoid-producing Lamiaceae species, *Callicarpa americana*

**DOI:** 10.1093/gigascience/giaa093

**Published:** 2020-09-07

**Authors:** John P Hamilton, Grant T Godden, Emily Lanier, Wajid Waheed Bhat, Taliesin J Kinser, Brieanne Vaillancourt, Haiyan Wang, Joshua C Wood, Jiming Jiang, Pamela S Soltis, Douglas E Soltis, Bjoern Hamberger, C Robin Buell

**Affiliations:** Department of Plant Biology, Michigan State University, 612 Wilson Road, East Lansing, MI 48824, USA; Florida Museum of Natural History, University of Florida, 3215 Hull Road, Gainesville, FL 32611, USA; Department of Biochemistry & Molecular Biology, Michigan State University, 603 Wilson Rd, East Lansing, MI 48824, USA; Department of Biochemistry & Molecular Biology, Michigan State University, 603 Wilson Rd, East Lansing, MI 48824, USA; Florida Museum of Natural History, University of Florida, 3215 Hull Road, Gainesville, FL 32611, USA; Department of Biology, University of Florida, 876 Newell Dr, Gainesville, Florida, 32611 USA; Department of Plant Biology, Michigan State University, 612 Wilson Road, East Lansing, MI 48824, USA; Department of Plant Biology, Michigan State University, 612 Wilson Road, East Lansing, MI 48824, USA; Department of Plant Biology, Michigan State University, 612 Wilson Road, East Lansing, MI 48824, USA; Department of Plant Biology, Michigan State University, 612 Wilson Road, East Lansing, MI 48824, USA; Department of Horticulture, Michigan State University, 1066 Bogue St, East Lansing, MI 48824, USA; MSU AgBioResearch, Michigan State University, 446 W. Circle Drive, East Lansing, MI 48824, USA; Florida Museum of Natural History, University of Florida, 3215 Hull Road, Gainesville, FL 32611, USA; Florida Museum of Natural History, University of Florida, 3215 Hull Road, Gainesville, FL 32611, USA; Department of Biology, University of Florida, 876 Newell Dr, Gainesville, Florida, 32611 USA; Department of Biochemistry & Molecular Biology, Michigan State University, 603 Wilson Rd, East Lansing, MI 48824, USA; MSU AgBioResearch, Michigan State University, 446 W. Circle Drive, East Lansing, MI 48824, USA; Department of Plant Biology, Michigan State University, 612 Wilson Road, East Lansing, MI 48824, USA; MSU AgBioResearch, Michigan State University, 446 W. Circle Drive, East Lansing, MI 48824, USA; Plant Resilience Institute, Michigan State University, 612 Wilson Road, East Lansing, MI 48824, USA

**Keywords:** beautyberry, callicarpenal, clerodane, gene cluster, insect repellent, kolavenyl diphosphate, specialized metabolites, terpene synthase

## Abstract

**Background:**

Plants exhibit wide chemical diversity due to the production of specialized metabolites that function as pollinator attractants, defensive compounds, and signaling molecules. Lamiaceae (mints) are known for their chemodiversity and have been cultivated for use as culinary herbs, as well as sources of insect repellents, health-promoting compounds, and fragrance.

**Findings:**

We report the chromosome-scale genome assembly of *Callicarpa americana* L. (American beautyberry), a species within the early-diverging Callicarpoideae clade of Lamiaceae, known for its metallic purple fruits and use as an insect repellent due to its production of terpenoids. Using long-read sequencing and Hi-C scaffolding, we generated a 506.1-Mb assembly spanning 17 pseudomolecules with N50 contig and N50 scaffold sizes of 7.5 and 29.0 Mb, respectively. In all, 32,164 genes were annotated, including 53 candidate terpene synthases and 47 putative clusters of specialized metabolite biosynthetic pathways. Our analyses revealed 3 putative whole-genome duplication events, which, together with local tandem duplications, contributed to gene family expansion of terpene synthases. Kolavenyl diphosphate is a gateway to many of the bioactive terpenoids in *C. americana*; experimental validation confirmed that *CamTPS2* encodes kolavenyl diphosphate synthase. Syntenic analyses with *Tectona grandis* L. f. (teak), a member of the Tectonoideae clade of Lamiaceae known for exceptionally strong wood resistant to insects, revealed 963 collinear blocks and 21,297 *C. americana* syntelogs.

**Conclusions:**

Access to the *C. americana* genome provides a road map for rapid discovery of genes encoding plant-derived agrichemicals and a key resource for understanding the evolution of chemical diversity in Lamiaceae.

## Data Description

### Introduction

**Figure 1:**
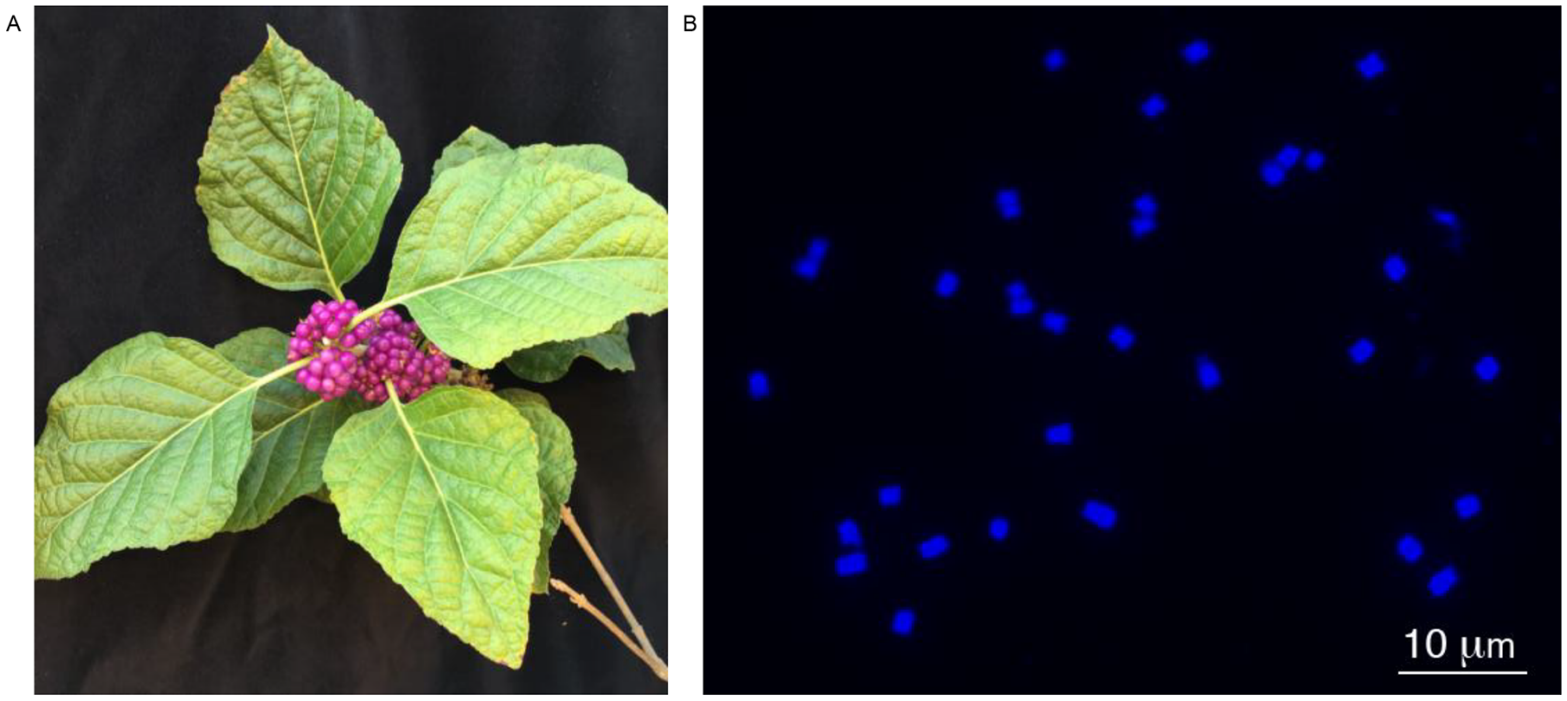
A, *Callicarpa americana* L. (beautyberry) plant with fruit. B, Somatic chromosome squash of a root tip cell of *C. americana* with 2*n* = 34. Bar = 10 μm.

Mints (Lamiaceae) are the sixth largest family of flowering plants and include many species grown for use as culinary herbs (basil, rosemary, thyme), food additives and flavorings (peppermint, spearmint), pharmaceuticals and health-promoting activities (skullcap, bee balm), feline euphoria induction (catnip), wood (teak), fragrance (lavender, patchouli), insect repellents (peppermint, rosemary), and ornamentals (coleus, chaste tree, beautyberry). This diverse set of uses for Lamiaceae is due in part to their production of specialized metabolites, primarily terpenes (monoterpenes, sesquiterpenes, diterpenes) and iridoids (irregular terpenes). Through an integrated phylogenetic-genomic-chemical approach, the evolutionary basis of Lamiaceae chemical diversity was shown to involve gene family expansion, differential gene expression, diversion of metabolic flux, and parallel evolution [1]. Genome sequences are currently available for a number of Lamiaceae species and are providing new insights into these phenomena, yet are primarily limited to members of Nepetoideae [2–5], the most species- and monoterpene-rich of the 12 major mint clades (=traditional subfamilies). As for the remaining major clades, a genome sequence is available only for *Tectona grandis* L. f. (teak; Tectonoideae) [6]. To expand our knowledge of the genome evolution underlying chemodiversity in this important family, we generated a chromosome-scale assembly of *Callicarpa americana* L. (American beautyberry, NCBI:txid204211), a species renowned for its charismatic purple fruits (Fig. 1A). *Callicarpa* occupies a pivotal phylogenetic position as a representative from the early-diverging mint lineage, Callicarpoideae [1]. The species is native to North America (southern USA, northern Mexico), North Atlantic (Bermuda, Bahamas), and Cuba, and has known insect repellent activity [7, 8] due to production of spathulenol, intermedeol, and callicarpenal [9]. Access to its genome will enable discovery of the genes encoding the biosynthetic pathways for these terpenes and the potential for heterologous expression of botanical-derived insect repellents; the genome is also an important evolutionary reference for the mint family.

### Plant material, DNA and RNA extraction, library preparation, and sequencing

Leaf tissue from a greenhouse-cultivated accession of *C. americana* (voucher: N. García 4530 [FLAS]) was harvested and frozen in liquid nitrogen. High molecular weight DNA for Pacific Biosciences (PacBio) libraries was extracted using a modified cetyl trimethylammonium bromide (CTAB) method (2% CTAB, 100 mM Tris, 1.4 M sodium chloride, 20 mM EDTA, 1% 2-mercaptoethanol) [10] and treated with RNase A. Large (>15 kb) insert libraries were constructed using the PacBio SMRTbell Template prep kit 1.0-SPV3 and sequenced on 11 PacBio Sequel SMRT Cells (Pacific Biosciences, Menlo Park, CA) at the University of Georgia Genomics and Bioinformatics Core. DNA was extracted from young leaf tissue using a modified CTAB method (2% CTAB, 100 mM Tris, 1.4 M sodium chloride, 20 mM EDTA, 1% 2-mercaptoethanol, 2% polyvinylpyrrolidone) method [10] and treated with RNase A. An Illumina-compatible 250-bp size-selected genomic paired-end library was constructed for use in error correction. Sequencing was performed on an Illumina HiSeq 4000 (Illumina HiSeq 3000/HiSeq 4000 System, RRID:SCR_016386) (Illumina, San Diego, CA) in paired-end mode, generating 150 nt reads. A proximity ligation (Hi-C) library was constructed from *C. americana* leaf tissue as described previously [11, 12] and sequenced on an Illumina HiSeq 4000. For transcriptome analyses, RNA was isolated from mature and young leaves, stems, petioles, roots, flowers (open and closed), and ripened whole fruits (denoted by the deep purple color) from growth chamber–grown plants using a hot phenol method [13]. Illumina TruSeq Stranded mRNA (polyA mRNA) libraries were constructed and sequenced on an Illumina HiSeq 4000 to 150 nt in paired-end mode. All Illumina sequencing was performed at the Research Technology Support Facility at Michigan State University.

### Genome assembly

The average flow cytometry genome size estimate of *C. americana* was 538 Mb, and we assembled the genome using 45 Gb (81× coverage) PacBio reads (≥1 kb) using Canu v1.7 (Canu, RRID:SCR_015880) [14] (Supplementary Tables S1 and S2) with the parameters minReadLength = 1000 genomeSize = 530m. The Canu assembly was polished with 2 rounds of Arrow v2.2.2 [15] using alignments of the PacBio reads generated with pbalign v0.3.1 [16]. Final polishing was then performed with Pilon v1.22 (Pilon, RRID:SCR_014731) [17] using whole-genome shotgun (WGS) Illumina reads that were trimmed using Cutadapt v1.15 (Cutadapt, RRID:SCR_011841) [18] with the parameters -n 2 -m 100 -q 10 and aligned to the assembly with BWA-MEM v0.7.17 (BWA-MEM, RRID:SCR_010910) [19]. The polished Canu contigs (965 total) had an N50 of 7,510,543 bp totaling 506,106,333 bp (Table 1), consistent with the estimated genome size. A chromosome count was performed using root tips as described previously [20], revealing 34 chromosomes (Fig. 1B); because *C. americana* is diploid, this represents a haploid chromosome number of 17. The Canu contigs were then scaffolded into 17 pseudochromosomes using the Hi-C reads (Supplementary Table S1) and the Phase Genomics Proximo Hi-C genome scaffolding platform as described in Jibran et al. [21]. The final assembly has an N50 scaffold size of 29,054,287 bp, representing 506,362,408 bp on 328 scaffolds; 493,744,786 bp are contained within the 17 pseudochromosomes, leaving 311 scaffolds representing 12,617,622 bp unanchored (Table 1).

**Table 1:** Metrics of final *Callicarpa americana* L. genome assembly

Feature	Metric
Canu-derived contigs	
N50 Contig size (bp)	7,510,543
NG50 Contig size (bp)	6,369,058
L50 Contig count	25
LG50 Contig count	27
Total assembly size (bp)	506,106,333
No. of contigs	965
Maximum contig length (bp)	18,804,173
Minimum contig length (bp)	1,028
Hi-C scaffolded assembly	
N50 Scaffold size (bp)	29,054,287
NG50 Scaffold size (bp)	28,692,425
L50 Scaffold count	8
LG50 Scaffold count	9
Total assembly size (bp)	506,362,408
No. of scaffolds	328
Maximum scaffold length (bp)	39,429,362
Minimum scaffold length (bp)	1,028
No. of pseudomolecules	17
Total pseudomolecule size (bp)	493,744,786
No. of unanchored scaffolds	311
Total unanchored scaffolds size (bp)	12,617,622
Pseudomolecules (bp)	
Chr01	39,429,362
Chr02	32,953,817
Chr03	32,428,638
Chr04	32,381,817
Chr05	31,681,419
Chr06	31,029,626
Chr07	29,370,463
Chr08	29,054,287
Chr09	28,692,425
Chr10	28,677,202
Chr11	28,224,296
Chr12	27,270,263
Chr13	27,197,714
Chr14	27,108,606
Chr15	23,772,120
Chr16	22,946,943
Chr17	21,525,788

To assess the genic representation in the final assembly, RNA-sequencing (RNA-seq) reads from 8 libraries (Supplementary Table S1) were processed using Cutadapt (v1.15; -n 2 -m 100 -q 10 [18]) to trim adapters and remove low-quality sequence. Cleaned RNA-seq reads were aligned to the genome using HiSAT2 v2.1.0 (HiSAT2, RRID:SCR_015530) [22] with the parameters –max-intronlen 5000 –rna-strandness RF, revealing a mean alignment percentage of 96.03% (Supplementary Table S3). Analysis using BUSCO v3.0.2 (BUSCO; RRID:SCR_015008) [23] with the Embryophyta v9 database revealed 93.8% complete orthologs (1,351), of which 1,241 (86.2%) were single copy and 110 (7.6%) were duplicated; 1.3% of the orthologs were fragmented (19), and 4.9% (70) were missing. Collectively, these data demonstrate a high-quality assembly of the *C. americana* genome.

To estimate the heterozygosity of the genome, canonical *k*-mers (*k* = 21) from the Illumina WGS reads were counted using Jellyfish2 v2.2.9 (Jellyfish2, RRID:SCR_005491) [24]. The *k*-mer count histogram was analyzed by the online version of GenomeScope (RRID:SCR_017014) [25, 26], and the heterozygosity of the genome was estimated at 0.158% (Supplementary Fig. S1).

### Genome annotation

To annotate the genome, we generated a species-specific custom repeat library using RepeatModeler v1.0.8 (RepeatModeler, RRID:SCR_015027) [27]. Protein-coding genes were removed using ProtExcluder (v1.1 [28]), and Viridiplantae repeats from RepBase [29] were used to create a final custom repeat library that was used to mask the genome. Repeat-masked versions of the genome were generated using RepeatMasker v4.0.6 (RepeatMasker, RRID:SCR_012954) [30] (-s -nolow -no_is -gff); in total, 55.9% of the genome was masked. Genome-guided transcripts were assembled from the HISAT2 v2.1.0 (HISAT2, RRID:SCR_015530) [31] (–max-intronlen 5000 –rna-strandness RF) alignments of each RNA-seq library using Trinity v2.6.6 (Trinity, RRID:SCR_013048) (–SS_lib_type RF –min_contig_length 500 –genome_guided_max_intron 5000 –genome_guided_bam; Supplementary Table S3 [32]). To train AUGUSTUS, genome-guided RNA-seq alignments from the young leaf library were used as evidence; initial gene predictions were made on the hard-masked assembly. Gene models were improved using PASA2 v2.1.0 (PASA2, RRID:SCR_014656) [33, 34] and the individual library genome-guided transcript assemblies as transcript evidence. Two rounds of annotation comparison were performed to generate the working gene model set ,which comprised 36,480 genes (loci) encoding 67,826 gene models (Table 2).

**Table 2:** *Callicarpa americana* L. gene annotation summary

	Working model set	High-confidence model set
No. of gene models	67,826	62,993
No. of loci	36,480	32,164
Maximum transcript length (bp)	16,862	15,978
Maximum CDS length (bp)	16,269	15,294
Mean transcript length (bp)	2,004.6	2,096.2
Mean CDS length (bp)	1,305.6	1,355.2
Mean exon length (bp)	323.5	323.8
Mean intron length (bp)	500.4	497.4
Single exon transcripts	18,140	14,496

CDS: coding sequence.

High-confidence gene models were identified using protein domain and gene expression abundance. Working gene models were searched against PFAM v32 (Pfam, RRID:SCR_004726) [35] with hmmscan (HMMER v3.1b2; RRID:SCR_005305) with a cut-off of –domE 1e-3 -E 1e-5. Gene expression values (transcripts per million [TPM]; Supplementary Table S4) for the working gene model set were generated using Kallisto v0.45.0 (Kallisto, RRID:SCR_016582) [36] and cleaned RNA-seq reads from each library. Gene models were identified as high confidence if they had a TPM value >0 in ≥1 RNA-seq library and/or had a PFAM domain match. Partial gene models and models with matches to transposable element–related PFAM domains were excluded from the high-confidence model set. Functional annotation was assigned by first searching the gene model–predicted proteins against the *Arabidopsis* proteome (TAIR10, RRID:SCR_004618) [37], the PFAM database v32 (Pfam, RRID:SCR_004726) [38], and Swiss-Prot plant proteins (release 2015_08) (UniProtKB, RRID:SCR_004426). The search results were processed in the same order, and the function of the first hit encountered was assigned to the gene model. The final high-confidence gene set contained 32,164 loci encoding 62,993 gene models (Table 2).

## Comparative Genome Analyses

**Figure 2:**
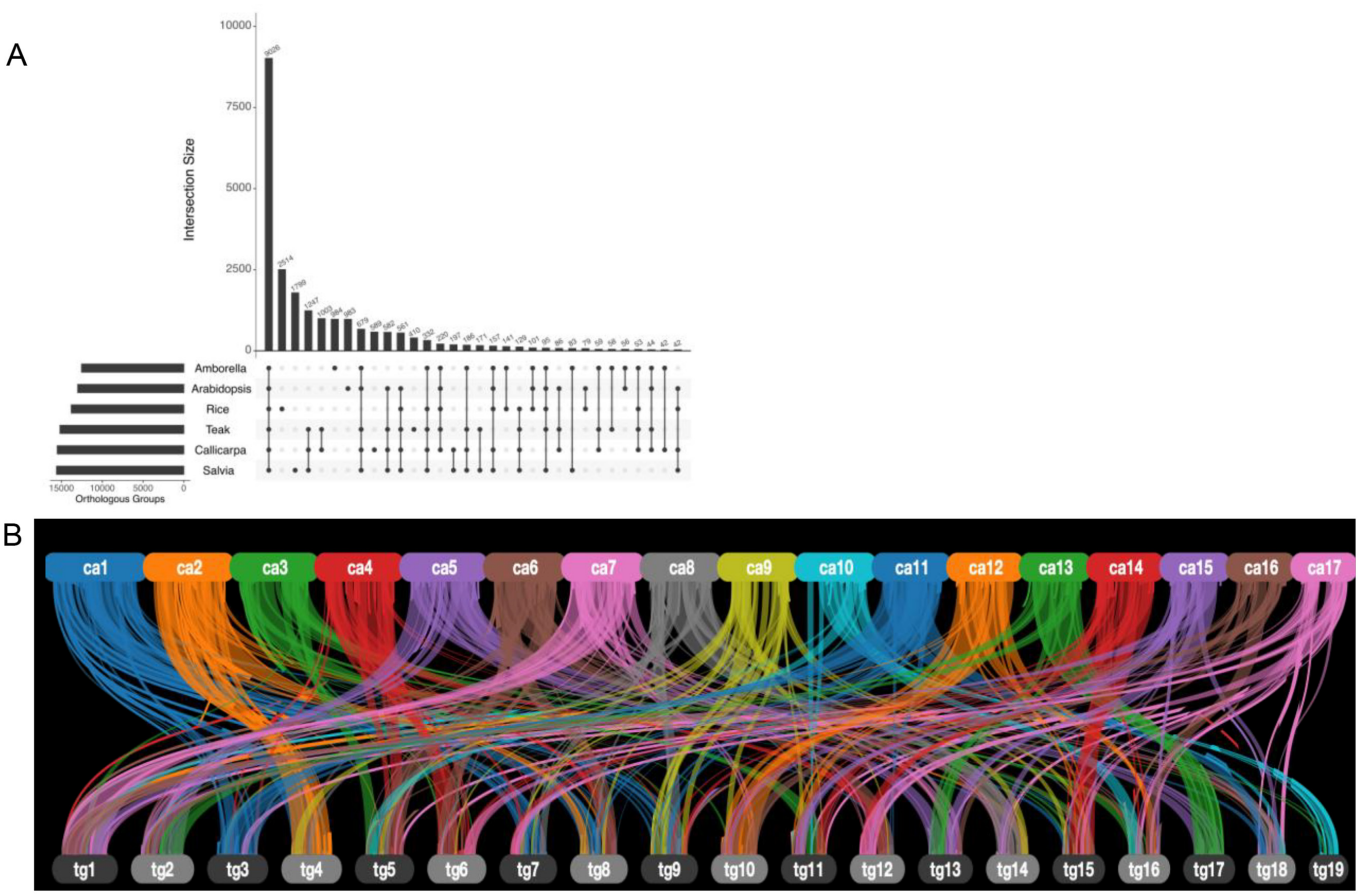
Comparative genome analyses with *Callicarpa americana* L. A. Upset plot showing orthologous groups between *C. americana* and 5 other angiosperms: *Amborella trichopoda* Baill [40] (*Amborella*), *Oryza sativa* L. (Rice, MSU v7), *Arabidopsis thaliana* (L.) Heynh (Araport 11 [42]) and 2 Lamiaceae species, *Tectona grandis* (teak, Tectonoideae [6]) and *Salvia splendens* Ker Gawl. (scarlet sage; Nepetoideae [4]). Only the 30 largest intersections are shown. B. Syntenic relationship between *T. grandis* (teak) and *C. americana* (beautyberry). The upper row shows the 17 *C. americana* pseudomolecules with syntenic alignments to the 19 *T. grandis* pseudomolecules.

*Callicarpa* is the only genus of Callicarpoideae, with ∼170 species. In addition to being the first species of *Callicarpa* with a genome sequence, the *C. americana* genome is useful for comparative studies because of its phylogenetic position within an early-diverging mint lineage. To better understand orthologous relationships within Lamiaceae, we used Orthofinder v2.3.7 (Orthofinder, RRID:SCR_017118) [39] with 6 angiosperm species: *Callicarpa americana* (this study), *Amborella trichopoda* Baill [40] (*Amborella*), *Oryza sativa* L. (Rice, MSU v7 [41]), *Arabidopsis thaliana* (L.) Heynh (Araport 11 [42]), and 2 Lamiaceae species: *Tectona grandis* (teak, Tectonoideae [6]) and *Salvia splendens* Ker Gawl. (scarlet sage; Nepetoideae [4]) (Fig. 2) to define orthologous and paralogous clusters. A total of 9,026 orthologous groups contained ≥1 protein from each of the 6 species (Fig. 2A) [1]. *T. grandis* (Tectonoideae), *S. splendens* (Nepetoideae), and *C. americana* (Callicarpoideae) represent 3 major subclades of Lamiaceae; the OrthoFinder analysis identified 1,247 orthogroups that were unique to Lamiaceae. Gene ontology (GO) terms were assigned to the *C. americana*–predicted proteome by searching the representative gene models against the Interpro databases using InterProScan v5.34.73.0 (InterProScan, RRID:SCR_005829) [43]. TopGO v2.36.0 (TopGO, RRID:SCR_014798) [44] analysis of Lamiaceae-specific genes revealed numerous biological process terms associated with response to stress (Supplementary Table S5), including defense response (GO:0006952), response to wounding (GO:0009611), and innate immunity (GO:0045087). Species of Lamiaceae are well known for their chemical diversity [1], and Lamiaceae-specific orthologous groups were enriched in molecular function terms including oxidoreductase activity (GO:0016705; GO:0016702), catechol oxidase activity (GO:0004097), and transferase activity (GO:0004097; GO:0016758) (Supplementary Table S5).

**Figure 3:**
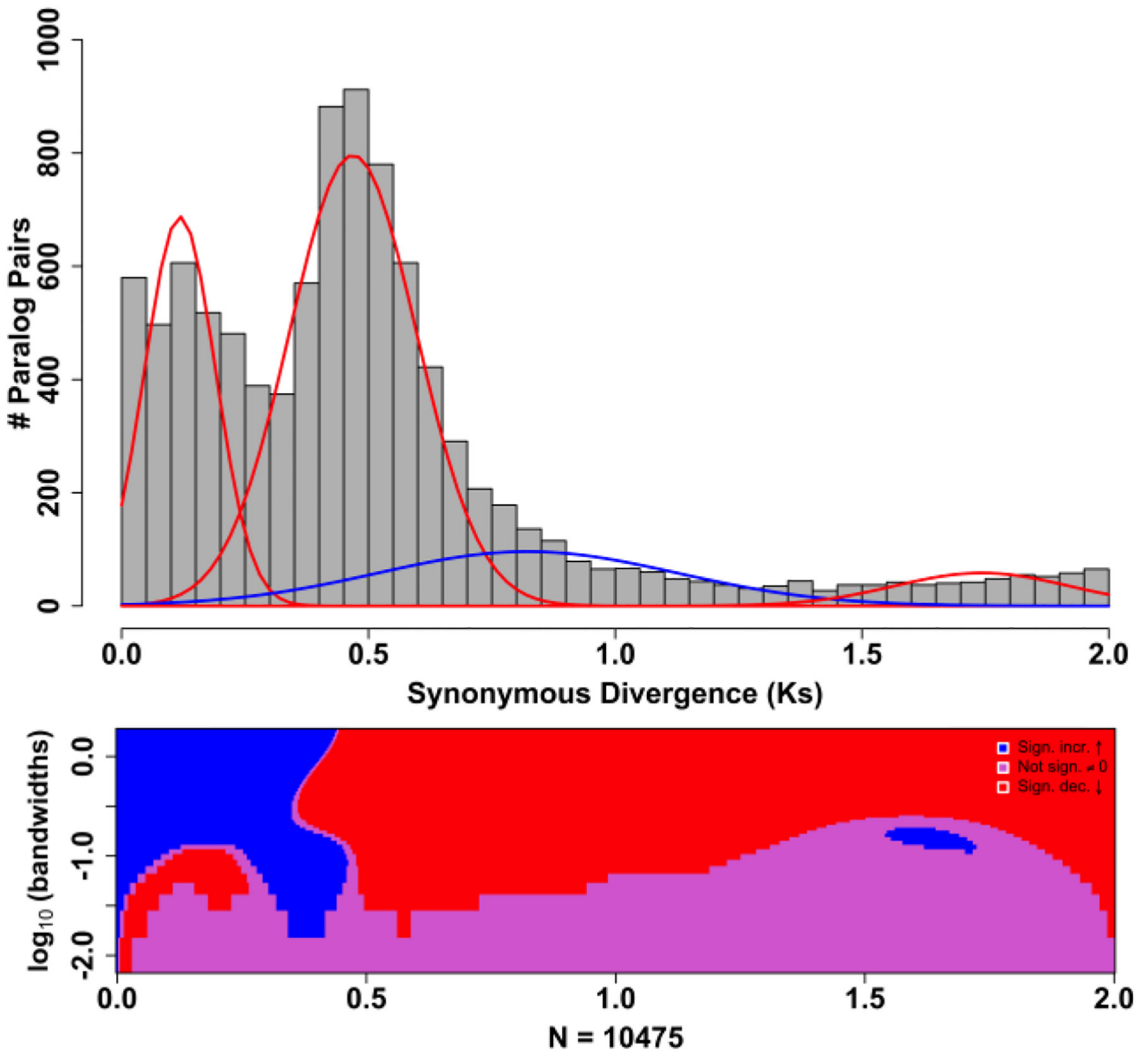
Whole-genome duplication (WGD) events inferred from the *Callicarpa americana* L. (beautyberry) genome. Gaussian distributions produced by mixture models in the mixtools R package [49] are shown as overlays on the *K*_S_ distribution, with red or blue color-coded peaks representing putative WGD events that were either corroborated or not corroborated (i.e., false-positive results), respectively, by results from SiZer analysis ([50]; lower plot). The SiZer plot shows significant increases (blue) or decreases (red), or no significant changes (pink) across the *K*_S_ distribution at various (log-transformed) bandwidths to distinguish true data features from noise.

Synteny analyses between *T. grandis* and *C. americana* were performed with MCScanX (git commit 7b61f32 [45, 46]) to identify inter-species collinear blocks. We identified 963 collinear blocks, representing 456 Mb of unique *C. americana* sequence; 31,235 *C. americana* genes were present in the collinear blocks, of which 21,297 were syntelogs with *T. grandis* (Fig. 2B). Ancient whole-genome duplication (WGD) events were inferred from estimates of divergence at synonymous sites (*K*_S_) among paralogous gene pairs present in the *C. americana* genome and compared with previous transcriptome-based inferences [47]. Coding sequences representing the longest isoform of each gene were filtered from the high-confidence gene set and analyzed with DupPipe using default settings [48]. Following an analysis workflow used previously with Lamiaceae [6, 47], significant peaks in the observed *K*_S_ distribution were identified with Gaussian mixture models, as implemented in the mixtools R package [49], and corroborated with results from a SiZer analysis [50]. Four components were predicted by the mixture models (Supplementary Table S6; Fig. 3), although only mean values at *K*_S_ = 0.12, 0.47, 1.74 were supported as significant data features by SiZer results, providing evidence for 3 ancient WGD events in *C. americana*. Of these putative WGDs, events placed at *K*_S_ = 0.12 and *K*_S_ = 1.74 were not previously detected or supported by transcriptome-based analyses, highlighting the benefits of WGD inferences from genomic data (discussed by Godden et al. [47]). We found no evidence for a shared ancient WGD on the basis of *K*_S_ results for *C. americana* and *T. grandis* using genomic data. Only one putative ancient WGD event (*K*_S_ = 0.60) was detected in *T. grandis* [6, 47], and available chromosome counts (i.e., 2*n* = 16 or 18 in *Callicarpa*vs 2*n* = 36 in *Tectona* [51]) suggest that *Tectona* has experienced ≥1 unique WGD event following its divergence from its common ancestor with *C. americana*. Moreover, results from recent phylotranscriptomic analyses [47] are most consistent with independent WGDs in (i) the common ancestor of *Callicarpa, Westringia*, and *Prostanthera* and (ii) the common ancestor of all remaining Lamiaceae, indicating that the WGDs of *Callicarpa* and *Tectona* are not shared. In contrast, one analysis of transcriptomic data shows a single WGD in the ancestor of Lamiaceae, suggesting that *Callicarpa* and *Tectona* share an ancestral WGD [47]. However, the genome-based *K*_S_ results do not show this pattern, and the overall results indicate independent WGDs in these two early branches of mint phylogeny.

## Specialized Metabolite Analyses

**Figure 4:**
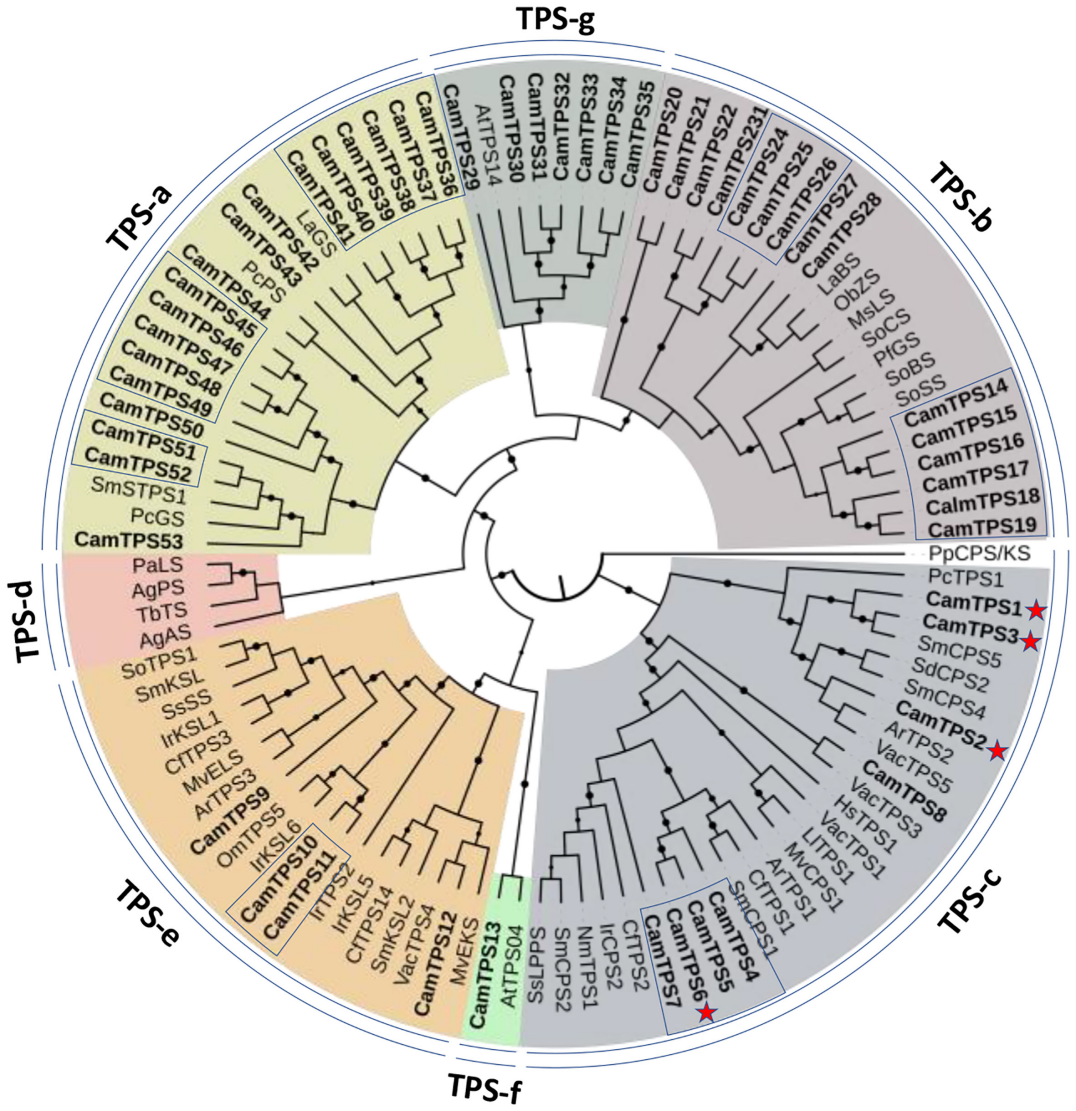
Phylogenetic analysis and classification of the *Callicarpa americana* terpene synthase family [6]. Shown are the distinct terpene synthase gene families TPS-a through TPS-g. Highlighted in boxes are TPSs clustered in proximity on the genomic pseudomolecules. *C. americana* TPSs are in boldface; red stars indicate functionally characterized members of the TPS-c subfamily; dots on branches indicate bootstrap support ≥80%. The phylogeny was rooted with the bifunctional *Physcomitrella patens* (moss) PpCPS/EKS. Annotation of *C. americana* and reference TPSs are given in Supplementary Tables S7 and S8.

**Figure 5:**
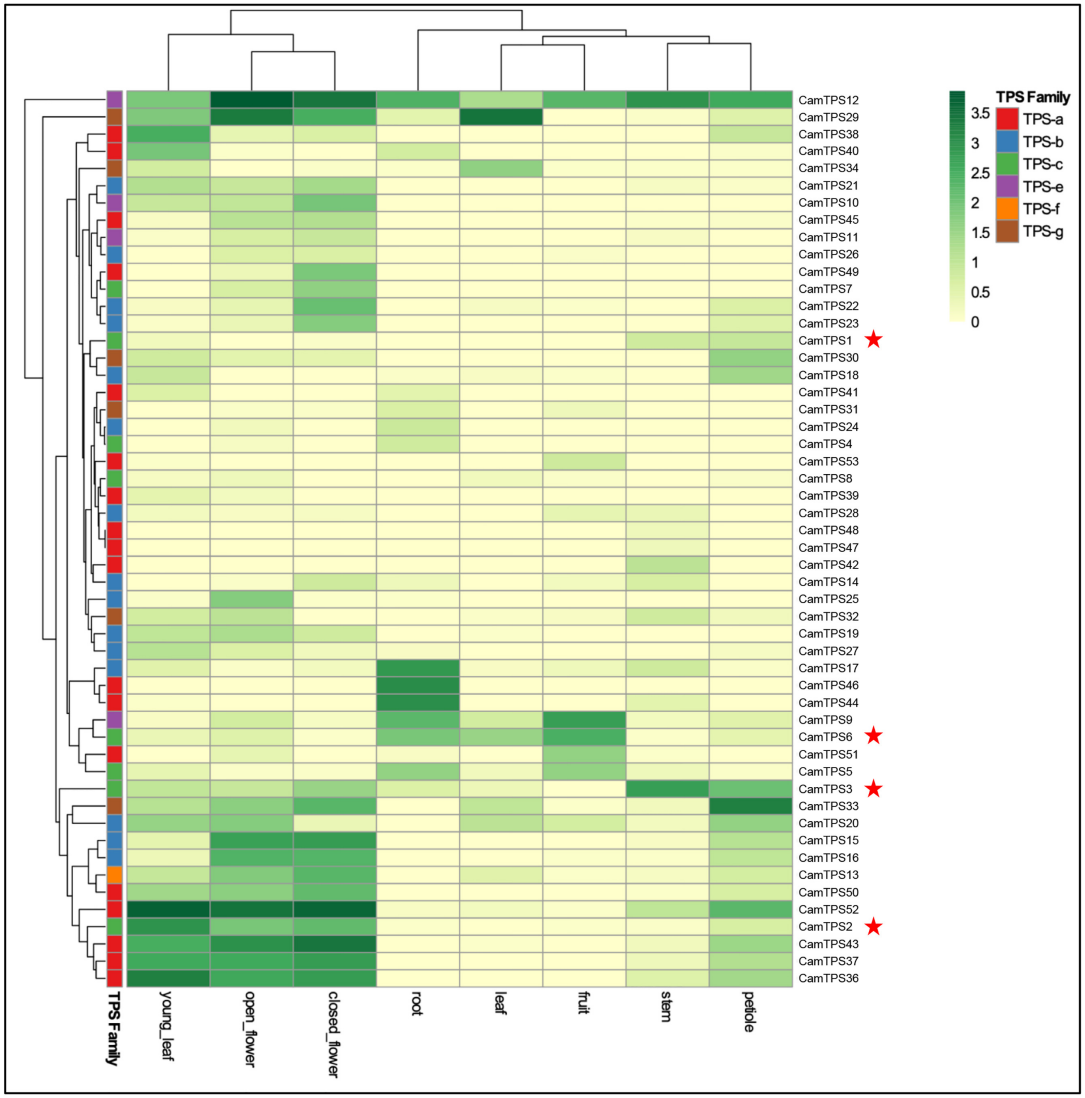
Tissue-specific expression of the *Callicarpa americana* L. terpene synthase gene family. Expression is in transcripts per million. TPS subfamily classification of *C. ameri*cana TPSs is given in Supplementary Table S8. Red stars indicate functionally characterized members of the TPS-c subfamily.

**Figure 6:**
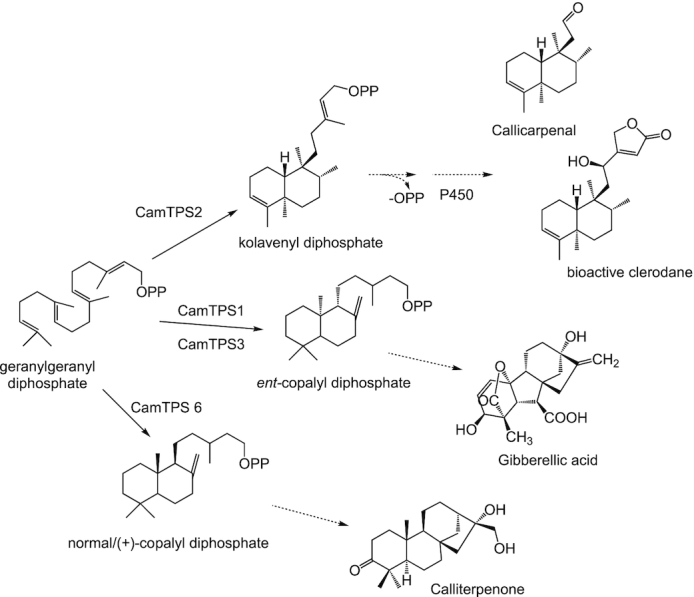
Activities of functionally characterized *Callicarpa americana* L. TPS-c. Dotted arrows indicate putative further functionalization by Class I diTPS and cytochromes P450 to diterpene products accumulating in *C. americana*.

*Callicarpa americana* produces a range of bioactive diterpenoids derived from the C_20_ clerodane skeleton [52], a less common instance of the labdanoid diterpenes. These include the C_16_ nor-diterpenoid (-)-callicarpenal, with a range of mosquito-, tick-, and arthropod-repellent activities [8]. Clerodane-type diterpenes are derived from the precursor kolavenyl diphosphate (KPP), which is formed by Class II diterpene synthases (diTPS) of the terpene synthase c (TPS-c) subfamily [53–55]. Here, we describe the annotation and validation of the KPP synthase in *C. americana*, a gateway to many of its bioactive terpenoids. Using the assembled genomic and transcriptomic data, we performed a sequence similarity search with BLASTP comparing the *C. americana* peptide models against a set of reference TPSs (Supplemental Text). Peptides shorter than 350 amino acids or having <30% identity to the most similar reference sequence were filtered out, yielding a total of 53 candidate TPSs (Supplementary Table S7). We used phylogenetic clustering (Fig. 4; Supplemental Text) with known TPSs to identify and classify candidates most likely to catalyze the formation of KPP. The complement and distribution of TPSs discovered was found in accordance with plant species [56], reflecting general metabolism and species-specific evolution of specialized metabolism in *C. americana*. Specifically, our study resulted in 8 putative diTPSs from the TPS-c subfamily; Class II diTPSs are typically involved in formation of the necessary diphosphate intermediates of the labdane-type chemistry. Of the 8 candidates, 4 were successfully cloned from complementary DNA (cDNA) and transferred into the plant expression vector pEAQ [6] as described previously. The others were not further pursued owing to low expression levels or a lack of expression in tissues relevant for callicarpenal formation. Expression analysis of tissue-specific accumulation of transcripts for the diTPSs (Fig. 5) showed the highest expression in young leaves and flowers for *CamTPS2*, consistent with the presence of callicarpenal in leaves. Characterization of the candidates through transient expression in *Nicotiana benthamiana*, and gas chromatography–mass spectrometry (GC-MS) analysis as described previously ([57]; Supplemental Text), showed that CamTPS1 and CamTPS3 catalyze the formation of *ent*-copalyl diphosphate (Fig. 6), the first step in the biosynthesis of the ubiquitous *ent*-kaurane type plant growth hormone gibberellic acid and specialized metabolites in the *ent*-configuration found in this genus. CamTPS6 yielded (+)-copalyl diphosphate, precursor of calliterpenone, a rare (+)-kaurane type diterpene found across several species of *Callicarpa* [52]. (+)-Copalyl diphosphate is also the intermediate to the common diterpene miltiradiene, precursor to many defense-related diterpenoids found in other Lamiaceae and previously identified in other *Callicarpa* species [52]. Finally, CamTPS2 was confirmed to yield the possible precursor of callicarpenal, KPP (Fig. 6). All products were confirmed by comparison with reference combinations of diTPS. Specifically, diTPS yielded access to *ent*-copalyl diphosphate (*ent*-CPP, CamTPS1, and Cam TPS3, Supplementary Fig. S2A), CPP in normal configuration ((+)-CPP, CamTPS6, Supplementary Fig. S2B), and kolavenyl diphosphate (KPP, CamTPS2, Supplementary Fig. S2C), all plausible precursors to the known chemical diversity of diterpene scaffolds in *C. americana*.

Genes encoding some specialized metabolic pathways are found physically clustered in plant genomes [58, 59]. We utilized the PlantiSMASH analytical pipeline [60] to identify physically clustered specialized metabolic pathway genes (Table 3). The most frequent type of cluster encoded saccharides (15), terpenes (9), uncharacterized clusters (8), and alkaloids (5). Several clusters of *C. americana* TPSs indicate significant expansion of the family by local tandem duplications (Fig. 4). Consistent with earlier findings in *Salvia miltiorrhiza* and *T. grandis*, where the genes involved in miltiradiene biosynthesis were found clustered [2, 6], *CamTPS6* was identified as part of a large cluster of putative terpene biosynthetic genes, including *CamTPS9*, the gene encoding the subsequently acting Class I enzyme CamTPS9. The cluster also carries several genes encoding cytochromes P450 of relevant subfamilies of the CYP71 clan, the largest repository for enzymes involved in terpene functionalization [61].

**Table 3:** Physically clustered specialized metabolite biosynthetic pathways in *Callicarpa americana* L. as identified by PlantiSMASH

Type	No.
Alkaloid	5
Lignan	2
Lignan-saccharide	1
Polyketide	3
Saccharide	15
Saccharide-polyketide	1
Saccharide-terpene	2
Terpene	9
Terpene-polyketide	1
Uncharacterized	8
Total	47

## Conclusion

The insect-repellent activity of *C. americana* is due to the production of the terpenoids spathulenol, intermedeol, and callicarpenal [9], and access to a chromosome-scale genome assembly of *C. americana* permitted identification of kolavenyl diphosphate synthase, which synthesizes kolavenyl diphosphate, a precursor to callicarpenal. As the sixth largest angiosperm family, and with extensive chemical diversity, Lamiaceae are an ideal group for application of phylogenomic data-mining, a powerful approach for biosynthetic pathway discovery. Generation of the genome of *C. americana*, of the early-diverging Callicarpoideae clade of Lamiaceae, provides a road map for rapid discovery of genes encoding plant-derived agrichemicals and a key resource for understanding the evolution of both chemical diversity and mint genomes.

## Availability of Supporting Data and Materials

All sequences generated in this study are available in the NCBI SRA under BioProject PRJNA529675. The genome assembly, annotation files, expression matrix, and other supporting data can be accessed at the *GigaScience* GigaDB database [62]. Genbank accession identifiers for cloned TPSs are MT083919–MT083922. Original raw GC-MS data were deposited to Zenodo [63] and Metabolights [64] under accession MTBLS1983.

## Additional Files

Supplementary Table S1: RNA-Seq, whole-genome shotgun, and Hi-C libraries used in this study.

Supplementary Table S2. PacBio flow cells used in this study.

Supplementary Table S3. *Callicarpa americana* RNA-seq alignment and genome-guided assembly transcript metrics.

Supplementary Table S4. Expression abundances of *Callicarpa americana* genes.

Supplementary Table S5. Gene ontology enrichment analyses of Lamiaceae-specific genes.

Supplementary Table S6. Gaussian mixture modeling and SiZer results for the *K*_S_ distribution estimated from the genome and transcriptome of *Callicarpa americana* L. Shown here are the number of inferred components, along with their corresponding means (**μ**), mixing proportions (**λ**), and standard deviations (**σ**) estimated by mixtools. The number of components corroborated by a SiZer analysis is indicated in brackets, with corresponding values of **μ, λ**, and **σ** from mixture models denoted with an asterisk (*). Transcriptome-based results from Godden et al. [47].

Supplementary Table S7. Terpene synthases identified in this study.

Supplementary Table S8. GenBank protein identifiers of the TPSs used for construction of phylogenetic tree.

Supplementary Table S9. Manually curated phylogeny for terpene synthases.

Supplementary Figure S1. Estimated heterozygosity of *Callicarpa americana* L. as revealed by GenomeScope [25].

Supplementary Figure S2. GC/MS data for TPS enzymes investigated alongside reference diTPS enzymes. Each class II diTPS is paired with a characterized class I diTPS and elution time/mass spectra compared to a pair of reference diTPS. A, CamTPS1 and CamTPS3 paired with NmTPS2 produce *ent-*kaurene, thus confirming that CamTPS1 and CamTPS3 both produce *ent*-CPP. The reference pair of ZmAn2 + NmTPS2 makes *ent-*kaurene from *ent-*CPP. B, CamTPS6 paired with CfTPS3 makes miltiradiene, confirming activity as a (*+*)-CPP synthase. The reference pair NmTPS1 + CfTPS3 makes miltiradiene from (*+*)-CPP. C, CamTPS2 paired with ScSS makes kolavelool, confirming CamTPS2 as a KPP synthase. The reference pair ShTPS1 + ScSS makes kolavelool from KPP. Reference enzymes NmTPS1, NmTPS2 *Nepeta mussini*; CfTPS3, *Coleus forskohlii*; ZmAN2, *Zea mays*; SsSCS, *Salvia sclarea* [55, 57, 65–67].

Supplemental Text 1. Supplemental methods used in this study.

giaa093_GIGA-D-20-00049_Original_Submission

giaa093_GIGA-D-20-00049_Revision_1

giaa093_Response_to_Reviewer_Comments_Original_Submission

giaa093_Reviewer_1_Report_Original_Submission Niklas MÃ¤hler -- 3/25/2020 Reviewed

giaa093_Reviewer_2_Report_Original_Submission Sunil Kumar Sahu, PhD., -- 3/31/2020 Reviewed

giaa093_Reviewer_2_Report_Revision_1 Sunil Kumar Sahu, PhD., -- 7/12/2020 Reviewed

giaa093_Supplemental_Files

## Abbreviations

BLAST: Basic Local Alignment Search Tool; bp: base pairs; BUSCO: Benchmarking Universal Single-Copy Orthologs; BWA: Burrows-Wheeler Aligner; cDNA: complementary DNA; CPP: copalyl diphosphate; CTAB: cetyl trimethylammonium bromide; EDTA: ethylenediaminetetraacetic acid; Gb: gigabases pairs; GC-MS: gas chromatography–mass spectrometry; GO: Gene Ontology; kb: kilobase pairs; KPP: kolavenyl diphosphate; Mb: megabase pairs; mRNA: messenger RNA; NCBI: National Center for Biotechnology Information; PacBio: Pacific Biosciences; RNA-seq: RNA-sequencing; SMRT: single-molecule real-time; SRA: Sequence Read Archive; TPM: transcripts per million; TPS: terpene synthase; WGD: whole-genome duplication; WGS: whole-genome shotgun.

## Competing Interests

The authors declare that they have no competing interests.

## Funding

Funds for this study were provided by a grant to C.R.B., D.E.S., and P.S.S. from the National Science Foundation Plant Genome Research Program (IOS-1444499), a grant to C.R.B. and B.H. from the Michigan State University Strategic Partnership Grants Program, and from Hatch funds to C.R.B. (MICL02431). B.H. gratefully acknowledges the U.S. Department of Energy-Great Lakes Bioenergy Research Center Cooperative Agreement DE-FC02-07ER64494 and DE-SC0018409, the Michigan State University Strategic Partnership Grant program “Plant-inspired Chemical Diversity,” startup funding from the Department of Molecular Biology and Biochemistry, Michigan State University, and support from Michigan State University AgBioResearch (MICL02454).

## Authors' Contributions

J.P.H. performed the genome assembly, annotation, and comparative analyses. B.V. and J.C.W. isolated nucleic acids and performed quality assessments. H.W. and J.J. performed the chromosome counting. G.G. and T.J.K. performed the whole-genome duplication analyses. B.H., E.L., D.E.S., P.S.S., and C.R.B. designed the experiments. W.W.B. performed the phylogenetic analyses and built the CamTPS repository. E.L. identified and functionally characterized the terpene synthases. C.R.B., J.H., G.T.G., and B.H. wrote the manuscript. All authors approved the final manuscript.
