# Supplementary material for: Generation of a chromosome-scale genome assembly of the insect-repellent terpenoid-producing Lamiaceae species, Callicarpa americana
Source: Gigascience. 2020 Sep 7;9(9):giaa093. doi: 10.1093/gigascience/giaa093 (PMC7476102; doi:10.1093/gigascience/giaa093)
Supplement: giaa093_GIGA-D-20-00049_Original_Submission [file giaa093_giga-d-20-00049_original_submission.pdf]

GigaScience

Generation of a chromosome-scale genome assembly of the insect-repellent terpenoid-producing Lamiaceae species, Callicarpa americana

--Manuscript Draft--

**Manuscript Number:**	GIGA-D-20-00049
**Full Title:**	Generation of a chromosome-scale genome assembly of the insect-repellent terpenoid-producing Lamiaceae species, Callicarpa americana
**Article Type:**	Data Note

Funding Information:

Funder	Recipient
Directorate for Biological Sciences (IOS-1444499)	Prof. C Robin Buell
US Department of Agriculture (MICL02431)	Prof. C Robin Buell
US Department of Agriculture (MICL02454)	Dr Bjoern Hamberger
US Department of Energy (DE-FC02-07ER64494)	Dr Bjoern Hamberger
US Department of Energy (DE-SC0018409)	Dr Bjoern Hamberger
Michigan State University Strategic Partnership Grant (None)	Dr Bjoern Hamberger<br>Prof. C Robin Buell

Abstract:

Background: Plants exhibit wide chemical diversity due to the production of specialized metabolites which function as pollinator attractants, defensive compounds, and signaling molecules. Lamiaceae (mints) are known for their chemodiversity and have been cultivated for use as culinary herbs as well as sources of insect repellents, health-promoting compounds, and fragrance. Findings: We report the chromosome-scale genome assembly of Callicarpa americana L . (American beautyberry) , a species within the early-diverging Callicarpoideae clade of Lamiaceae, known for its metallic purple fruits and used as an insect repellent due to its production of terpenoids. Using long read sequencing and Hi-C scaffolding, we generated a 506.1 Mb assembly spanning 17 pseudomolecules with N50 contig and N50 scaffold sizes of 7.5 Mb and 29.0 Mb, respectively. In all, 32,164 genes were annotated, including 53 candidate terpene synthases and 47 putative clusters of specialized metabolite biosynthetic pathways. Whole-genome duplication analyses revealed three putative events, which together with local tandem duplication events, contributed to gene family expansion of terpene synthases. Kolavenyl diphosphate is a gateway to many of C. americana 's bioactive terpenoids; experimental validation confirmed that CamTPS2 encodes kolavenyl diphosphate synthase. Syntenic analyses with Tectona grandis L. f. (teak), a member of the Tectonoideae clade of Lamiaceae known for exceptionally strong wood resistant to insects, revealed 963 collinear blocks and 21,297 C. americana syntelogs. Conclusions: Access to the C. americana genome provides a roadmap for rapid discovery of genes encoding plant-derived agrichemicals and a key resource for understanding the evolution of chemical diversity in Lamiaceae.

**Corresponding Author:**	C Robin Buell<br>Michigan State University<br>East Lansing, Michigan UNITED STATES
**Corresponding Author Secondary Information:**	
**Corresponding Author's Institution:**	Michigan State University
**Corresponding Author's Secondary Institution:**	
**First Author:**	John P Hamilton
**First Author Secondary Information:**	
**Order of Authors:**	John P Hamilton

Powered by Editorial Manager® and ProduXion Manager® from Aries Systems Corporation

	Grant Godden
	Emily Lanier
	Wajid W Bhat
	Taliesin Kinser
	Brieanne Vaillancourt
	Haiyan Wang
	Joshua Wood
	Jiming Jiang
	Pamela S Soltis
	Douglas E Soltis
	Bjoern Hamberger
	C Robin Buell
**Order of Authors Secondary Information:**	
**Additional Information:**	
**Question**	**Response**
Are you submitting this manuscript to a special series or article collection?	No
**Experimental design and statistics**<br><br>Full details of the experimental design and statistical methods used should be given in the Methods section, as detailed in our Minimum Standards Reporting Checklist. Information essential to interpreting the data presented should be made available in the figure legends.<br><br>Have you included all the information requested in your manuscript?	Yes
**Resources**<br><br>A description of all resources used, including antibodies, cell lines, animals and software tools, with enough information to allow them to be uniquely identified, should be included in the Methods section. Authors are strongly encouraged to cite Research Resource Identifiers (RRIDs) for antibodies, model organisms and tools, where possible.	Yes

Have you included the information requested as detailed in our Minimum Standards Reporting Checklist?	
**Availability of data and materials**<br><br>All datasets and code on which the conclusions of the paper rely must be either included in your submission or deposited in publicly available repositories (where available and ethically appropriate), referencing such data using a unique identifier in the references and in the “Availability of Data and Materials” section of your manuscript.<br><br>Have you have met the above requirement as detailed in our Minimum Standards Reporting Checklist?	Yes

Powered by Editorial Manager® and ProduXion Manager® from Aries Systems Corporation

Full manuscript including figures, tables, supp. tables
Click here to download Manuscript Sub1.PDF

DATA NOTE

Generation of a chromosome-scale genome assembly of the insect-repellent terpenoid-producing Lamiaceae species, *Callicarpa americana*

John P. Hamilton[1], Grant T. Godden[2], Emily Lanier[3], Wajid Waheed Bhat[3], Taliesin J. Kinser[2,4], Brieanne Vaillancourt[1], Haiyan Wang[1], Joshua C. Wood[1], Jiming Jiang[1,5,6], Pamela S. Soltis[2], Douglas E. Soltis[2,4], Bjoern Hamberger[3,6], and C. Robin Buell[1,6,7]

[1]Department of Plant Biology, Michigan State University, East Lansing, MI 48824, USA
[2]Florida Museum of Natural History, University of Florida, Gainesville, FL 32611, USA
[3]Department of Biochemistry & Molecular Biology, Michigan State University, East Lansing, MI 48824, USA
[5]Department of Horticulture, Michigan State University, East Lansing MI 48824 USA
[6]MSU AgBioResearch, Michigan State University, East Lansing MI 48824 USA
[7]Plant Resilience Institute, Michigan State University, East Lansing MI 48824 USA

∗Correspondence address. C. Robin Buell, Department of Plant Biology, Michigan State University, 612 Wilson Road, East Lansing, MI 48824, USA, E-mail: buell@msu.edu
http://orcid.org/0000-0002-6727-4677

Keywords: beautyberry, callicarpenal, clerodane, gene cluster, insect repellent, kolavenyl diphosphate, specialized metabolites, terpene synthase

Abstract

Background: Plants exhibit wide chemical diversity due to the production of specialized metabolites which function as pollinator attractants, defensive compounds, and signaling molecules. Lamiaceae (mints) are known for their chemodiversity and have been cultivated for use as culinary herbs as well as sources of insect repellents, health-promoting compounds, and fragrance. *Findings:* We report the chromosome-scale genome assembly of *Callicarpa americana* L. (American beautyberry), a species within the early-diverging Callicarpoideae clade of Lamiaceae, known for its metallic purple fruits and use as an insect repellent due to its production of terpenoids. Using long read sequencing and Hi-C scaffolding, we generated a 506.1 Mb assembly spanning 17 pseudomolecules with N50 contig and N50 scaffold sizes of 7.5 Mb and 29.0 Mb, respectively. In all, 32,164 genes were annotated, including 53 candidate terpene synthases and 47 putative clusters of specialized metabolite biosynthetic pathways. Whole-genome duplication analyses revealed three putative events, which together with local tandem duplication events, contributed to gene family expansion of terpene synthases. Kolavenyl diphosphate is a gateway to many of *C. americana*'s bioactive terpenoids; experimental validation confirmed that *CamTPS2* encodes kolavenyl diphosphate synthase. Syntenic analyses with *Tectona grandis* L. f. (teak), a member of the Tectonoideae clade of Lamiaceae known for exceptionally strong wood resistant to insects, revealed 963 collinear blocks and 21,297 *C. americana* syntelogs. *Conclusions:* Access to the *C. americana* genome provides a roadmap for rapid discovery of genes encoding plant-derived agrichemicals and a key resource for understanding the evolution of chemical diversity in Lamiaceae.

Data Description

Introduction

Mints (Lamiaceae) are the sixth largest family of flowering plants and include many species grown for use as culinary herbs (basil, rosemary, thyme), food additives and flavorings (peppermint, spearmint), pharmaceuticals and health-promoting activities (skullcap, bee balm), feline euphoria induction (catnip), wood (teak), fragrance (lavender, patchouli), insect repellents (peppermint, rosemary), and ornamentals (coleus, chaste tree, beautyberry). This diverse set of uses for Lamiaceae is due in part to their production of specialized metabolites, primarily terpenes (monoterpenes, sesquiterpenes, diterpenes) and iridoids (irregular terpenes). Through an integrated phylogenetic-genomic-chemical approach, the evolutionary basis of Lamiaceae chemical diversity was shown to involve gene family expansion, differential gene expression, diversion of metabolic flux, and parallel evolution [1]. Genome sequences are currently available for a number of Lamiaceae species and are providing new insights into these phenomena, yet are primarily limited to members of Nepetoideae [2-5], the most species- and monoterpene-rich of the 12 major mint clades (= traditional subfamilies). As for the remaining major clades, a genome sequence is available only for *Tectona grandis* L. f. (teak; Tectonoideae) [6]. To expand our knowledge of the genome evolution underlying chemodiversity in this important family, we generated a chromosome-scale assembly of *Callicarpa americana* L. (American beautyberry), a species renowned for its charismatic purple fruits (Figure 1A). *Callicarpa* occupies a pivotal phylogenetic position as a representative from the early-diverging mint lineage, Callicarpoideae [1]. The species is native to North America (southern U. S. A., northern Mexico), North Atlantic (Bermuda, Bahamas), and Cuba, and has known insect repellent activity [7, 8] due to production of spathulenol, intermedeol, and callicarpenal [9]. Access to its genome will enable discovery of the genes encoding the biosynthetic pathways for these terpenes and the potential for heterologous expression of botanical-derived insect repellents; the genome is also an important evolutionary reference for the mint family.

Plant material, DNA and RNA extraction, library preparation, and sequencing

Leaf tissue from a greenhouse-cultivated accession of *C. americana* (voucher: N. García 4530 [FLAS]) was harvested and frozen in liquid nitrogen. High-molecular-weight DNA for Pacific Biosciences (PacBio) libraries was extracted using a modified cetyl trimethylammonium bromide (CTAB) method [10] and treated with RNase A [10]. Large (>15 kb) insert libraries were constructed using the Pacific Biosciences SMRTbell Template prep kit 1.0-SPV3 and sequenced on 11 PacBio Sequel SMRT Cells (Pacific Biosciences, Menlo Park, CA) at the University of Georgia Genomics and Bioinformatics Core. DNA was extracted from young leaf tissue using a modified CTAB method [10] and an Illumina-compatible 250-bp size selected genomic paired-end library was constructed for use in error correction. Sequencing was performed on an Illumina HiSeq 4000 (Illumina, San Diego, CA) in paired-end mode generating 150 nt reads. For transcriptome analyses, RNA was isolated from mature and young leaves, stems, petioles, roots, flowers (open and closed), and ripened whole fruits (denoted by the deep purple color) from growth-chamber-grown plants using a hot phenol method [11]. Illumina TruSeq Stranded mRNA (polyA mRNA) libraries were constructed and sequenced on an Illumina HiSeq 4000 to 150 nt in paired-end mode. All Illumina sequencing was performed at the Research Technology Support Facility at Michigan State University.

Genome assembly

The average flow cytometry genome size estimate of *C. americana* was 538 Mb, and we assembled the genome using 45 Gb (81x coverage) PacBio reads ($\geq$ 1 kb) using Canu (v1.7; [12]; Tables S1, S2). The Canu assembly was polished with two rounds of Arrow (v2.2.2; [13]). Final polishing was then performed with Pilon (v1.22; [14]) using whole-genome shotgun Illumina reads that were trimmed using Cutadapt (v1.15; [15]) and aligned to the assembly with BWA-MEM (v0.7.17; [16]). The polished Canu contigs (965 total) had an N50 of 7,510,543 bp totaling 506,106,333 bp (Table 1), consistent with the estimated genome size. A chromosome count was performed using root tips as described previously [17], revealing 34 chromosomes (Figure 1B); as *C. americana* is diploid, this represents a haploid chromosome number of 17. The Canu contigs were then scaffolded into 17 pseudochromosomes using the Phase Genomics Proximo Hi-C genome scaffolding platform [18]. The final assembly has an N50 scaffold size of

29,054,287 bp representing 506,362,408 bp on 328 scaffolds; 493,744,786 bp are contained within the 17 pseudochromosomes leaving 322 scaffolds representing 12,617,622 bp unanchored (Table 1).

To assess the genic representation in the final assembly, RNA-sequencing (RNA-seq) reads from eight libraries (Table S1) were processed using Cutadapt (v1.15; -n 2 -m 100 -q 10; [15]) to trim adapters and remove low-quality sequence. Cleaned RNA-seq reads were aligned to the genome using HiSAT2 (v2.1.0) [19] with the parameters: --max-intronlen 5000 --rna-strandness RF, revealing an average alignment percentage of 96.03% (Table S3). Analysis using Benchmarking Universal Single-Copy Orthologs (v3.0.2; [20]) with the Embryophyta v9 database revealed 93.8% complete orthologs (1,351), of which, 1,241 (86.2%) were single copy and 110 (7.6%) were duplicated; 1.3% of the orthologs were fragmented (19), and 4.9% (70) were missing. Collectively, these data demonstrate a high-quality assembly of the *C. americana* genome.

Genome annotation

To annotate the genome, we generated a species-specific custom repeat library using RepeatModeler (v1.0.8; [21]). Protein-coding genes were removed using ProtExcluder (v1.1; [22]), and Viridiplantae repeats from RepBase [23] were used to create a final custom repeat library that was used to mask the genome. Repeat-masked versions of the genome were generated using RepeatMasker (v4.0.6; [24]; -s -nolow -no_is -gff); in total, 55.9% of the genome was masked. Genome-guided transcripts were assembled from the HISAT2 (v2.1.0; --max-intronlen 5000 --rna-strandness RF; [25]) alignments of each RNA-seq library using Trinity (v2.6.6; --SS_lib_type RF --min_contig_length 500 --genome_guided_max_intron 5000 --genome_guided_bam; Table S3; [26]). To train AUGUSTUS, genome-guided RNA-seq alignments from the young leaf library were used as evidence; initial gene predictions were made on the hard-masked assembly. Gene models were improved using PASA2 (v2.1.0; [27, 28]) and the individual library genome-guided transcript assemblies as transcript evidence. Two rounds of

annotation comparison were performed to generate the working gene model set which comprised 36,480 genes (loci) encoding 67,826 gene models (Table 2).

High-confidence gene models were identified using protein domain and gene expression abundance. Working gene models were searched against PFAM (v32; [29]) with hmmscan (HMMER v3.1b2) with a cutoff of --domE 1e-3 -E 1e-5. Gene expression values (transcripts per million (TPM; Table S4)) for the working gene model set were generated using Kallisto (v0.45.0; [30]) and cleaned RNA-seq reads from each library. Gene models were identified as high confidence if they had a TPM value > 0 in at least one RNA-seq library and/or had a PFAM domain match. Partial gene models and models with matches to transposable element-related PFAM domains were excluded from the high-confidence model set. Functional annotation was assigned by first searching the gene model predicted proteins against the *Arabidopsis* proteome (TAIR10; [31]), the PFAM database (v32, [32]), and Swiss-Prot plant proteins (release 2015_08). The search results were processed in the same order and the function of the first hit encountered was assigned to the gene model. The final high-confidence gene set contained 32,164 loci encoding 62,993 gene models (Table 2).

Comparative genome analyses

Callicarpa is the only genus of Callicarpoideae, with ~170 species. In addition to being the first species of *Callicarpa* with a genome sequence, the *C. americana* genome is useful for comparative studies because of its phylogenetic position within an early-diverging mint lineage. To better understand orthologous relationships within Lamiaceae, we used Orthofinder2 (v2.3.7; [33]) with six angiosperm species: *Callicarpa americana* (this study), *Amborella trichopoda* Baill [34] (*Amborella*), *Oryza sativa* L. (Rice, MSU v7), *Arabidopsis thaliana* (L.) Heynh (Araport 11; [35]), and two Lamiaceae species: *Tectona grandis* (teak, Tectonoideae; [6]) and *Salvia splendens* Ker Gawl. (scarlet sage; Nepetoideae [4]) (Figure 2) to define orthologous and paralogous clusters. A total of 9,026 orthologous groups contained at least one protein from each of the six species (Figure 2A) [1]. *Tectona grandis* (Tectonoideae), *S. splendens* (Nepetoideae), and *C. americana* (Callicarpoideae) represent three major subclades of

Lamiaceae; the OrthoFinder2 analysis identified 1,247 orthogroups that were unique to Lamiaceae. Gene ontology (GO) terms were assigned to the *C. americana* predicted proteome by searching the representative gene models against the Interpro databases using Iprscan (v5.34.73.0; [36]). TopGO (v2.36.0; [37]). Analysis of Lamiaceae-specific genes revealed numerous biological process terms associated with response to stress (Table S5), including defense response (GO:0006952), response to wounding (GO:0009611), and innate immunity (GO:0045087). Species of Lamiaceae are well known for their chemical diversity [1], and Lamiaceae-specific orthologous groups were enriched in molecular function terms including oxidoreductase activity (GO:0016705; GO:0016702), catechol oxidase activity (GO:0004097), and transferase activity (GO:0004097; GO:0016758) (Table S5).

Synteny analyses between *T. grandis* and *C. americana* were performed with MCScanX (7b61f32; [38]) to identify inter-species collinear blocks. We identified 963 collinear blocks, representing 456 Mb of unique *C. americana* sequence; 31,235 *C. americana* genes were present in the collinear blocks, of which, 21,297 were syntelogs with *T. grandis* (Figure 2B). Ancient whole-genome duplication (WGD) events were inferred from estimates of divergence at synonymous sites (K_S) among paralogous gene pairs present in the *C. americana* genome and compared with previous transcriptome-based inferences [39]. Coding sequences representing the longest isoform of each gene were filtered from the high-confidence gene set and analyzed with DupPipe using default settings [40]. Following an analysis workflow used previously with Lamiaceae [6, 39], significant peaks in the observed K_S distribution were identified with Gaussian mixture models and corroborated with results from a SiZer analysis [41]. Of the four components predicted by the mixture models (Table S6; Figure 3), mean values at K_S = 0.12, 0.47, 1.74 were supported as significant data features by SiZer results, providing evidence for three ancient WGD events in *C. americana*. Of these putative WGDs, events placed at K_S = 0.12 and K_S = 1.74 were not previously detected or supported by transcriptome-based analyses, highlighting the benefits of WGD inferences from genomic data (discussed in [39]).

Specialized metabolite analyses

Callicarpa americana produces a range of bioactive diterpenoids derived from the C_{20} clerodane skeleton [42], a less common instance of the labdanoid diterpenes. These include the C_{16} nor-diterpenoid (-)-callicarpenal with a range of mosquito, tick, and arthropod repellent activities [8]. Clerodane-type diterpenes are derived from the precursor kolavenyl diphosphate (KPP), which is formed by class II diterpene synthases (diTPS) of the terpene synthase c (TPS-c) subfamily [43-45]. Here, we describe the annotation and validation of the KPP synthase in *C. americana*, a gateway to many of its bioactive terpenoids. Using the assembled genomic and transcriptomic data, we performed a sequence similarity search with BLASTP comparing the *C. americana* peptide models against a set of reference TPSs (Supplemental Text). Peptides shorter than 350 amino acids or having less than 30% identity to the most similar reference sequence were filtered out, yielding a total of 53 candidate TPSs (Table S7). We used phylogenetic clustering (Figure 4; Supplemental Text) with known TPSs to identify and classify candidates most likely to catalyze the formation of KPP. This resulted in eight putative diTPSs from the TPS-c subfamily; class II diTPSs are typically involved in formation of the necessary diphosphate intermediates of the labdane-type chemistry. Of the eight candidates, four were successfully cloned from cDNA and transferred into the plant expression vector pEAQ [6] as described previously. The others were not further pursued due to low expression levels or a lack of expression in tissues relevant for callicarpenal formation. Expression analysis of tissue-specific accumulation of transcripts for the diTPSs (Figure 5) showed the highest expression in young leaves and flowers for *CamTPS2*, consistent with the presence of callicarpenal in leaves. Characterization of the candidates through transient expression in *Nicotiana benthamiana*, as described previously ([46]; Supplemental Text),showed that CamTPS1 and CamTPS3 catalyze the formation of *ent*-copalyl diphosphate (Figure 6), the first step in the biosynthesis of the ubiquitous *ent*-kaurane type plant growth hormone gibberellic acid (GA) and specialized metabolites in the *ent*-configuration found in this genus. CamTPS6 yielded (+)-copalyl diphosphate, precursor of calliterpenone, a rare (+)-kaurane type diterpene found across several species of *Callicarpa* [42]. (+)-Copalyl diphosphate is also the intermediate to the common diterpene miltiradiene, precursor to many defense related diterpenoids found in other Lamiaceae and previously identified in other *Callicarpa* species [42]. Finally, CamTPS2 was

confirmed to yield the plausible precursor of callicarpenal, KPP (Figure 6). All products were confirmed by reference combinations of diTPS.

Genes encoding some specialized metabolic pathways are found physically clustered in plant genomes [47, 48]. We utilized the PlantiSMASH analytical pipeline [49] to identify physically clustered specialized metabolic pathway genes (Table 3). The most frequent type of cluster encoded saccharides (15), terpenes (9), uncharacterized clusters (8), and alkaloids (5). Several clusters of *C. americana* TPSs indicate significant expansion of the family by local tandem duplications (Figure 4). Consistent with earlier findings in *S. miltiorrhizza* and *T. grandis*, where the genes involved in miltiradiene biosynthesis were found clustered [2, 6], *CamTPS6* was identified as part of a large cluster of putative terpene biosynthetic genes, including *CamTPS9,* the gene encoding the subsequently acting class I enzyme CamTPS9. The cluster also carries several genes encoding cytochromes P450 of relevant subfamilies of the CYP71 clan, the largest repository for enzymes involved in terpene functionalization [50].

Conclusion

The insect repellent activity of *C. americana* is due to the production of the terpenoids spathulenol, intermedeol, and callicarpenal [9], and access to a chromosome-scale genome assembly of *C. americana* permitted identification of kolavenyl diphosphate synthase which synthesizes kolavenyl diphosphate, a precursor to callicarpenal. As the sixth largest angiosperm family, and with extensive chemical diversity, Lamiaceae are an ideal group for application of phylogenomic data-mining, a powerful approach for biosynthetic pathway discovery. Generation of the genome of *C. americana*, of the early-diverging Callicarpoideae clade of Lamiaceae, provides a roadmap for rapid discovery of genes encoding plant-derived agrichemicals and a key resource for understanding the evolution of both chemical diversity and mint genomes.

Availability of supporting data

All sequences generated in this study are available in the NCBI SRA under BioProject PRJNA529675. The genome assembly, annotation files, expression matrix, and other supporting data can be accessed at the GigaScience database GigaDB (doi to be provided) and via the Dryad Digital Repository (doi:10.5061/dryad.931zcrjgj; URL for reviewing purposes only: https://datadryad.org/stash/share/c7DVVdtsgpj2VmzLrjw_k3NOasck08EqHmAGwaDflFg). Genbank accession identifiers for cloned TPSs are (to be provided upon publication). Original raw GC-MS data were deposited to Zenodo (doi.org/10.5281/zenodo.3672159).

Additional files

Table S1: RNA-Seq, whole-genome shotgun, and Hi-C libraries used in this study.

Table S2. PacBio flow cells used in this study.

Table S3. *Callicarpa americana* RNA-seq alignment and genome-guided assembly transcript metrics.

Table S4. Expression abundances of *Callicarpa americana* genes.

Table S5. Gene ontology enrichment analyses of Lamiaceae-specific genes.

Table S6. Gaussian mixture modeling and SiZer results for the K_S distribution estimated from the genome and transcriptome of *Callicarpa americana* L. Shown here are the number of inferred components, along with their corresponding means ($\boldsymbol{\mu}$), mixing proportions ($\boldsymbol{\lambda}$), and standard deviations ($\boldsymbol{\sigma}$) estimated by mixtools. The number of components corroborated by a SiZer analysis is indicated in brackets, with corresponding values of $\boldsymbol{\mu}$, $\boldsymbol{\lambda}$, and $\boldsymbol{\sigma}$ from mixture models denoted with an asterisk (*). Transcriptome-based results from Godden et al. [39].

Figure S1. GC-MS analysis of extracts from *Nicotiana benthamiana* assays heterologously expressing reference diTPS and enzymes from *C. americana*. (A) Formation of miltiradiene

through (+)-copalyl diphosphate (CamTPS6), (B) *ent*-kaurene through *ent*-copalyl diphosphate (CamTPS1), and (C) kolavelool through kolavenyl diphosphate (CamTPS2). Reference enzymes NmTPS1, *Nepeta mussini*; CfTPS3, *Coleus forskohlii*; ZmAN2, *Zea mays*; SsSCS, *Salvia sclarea* [45, 46, 51-53].

Table S7. Terpene synthases identified in this study.

Table S8. GenBank protein identifiers of the TPSs used for construction of phylogenetic tree.

Abbreviations

BLAST: Basic Local Alignment Search Tool; BUSCO: Benchmarking Universal Single-Copy Orthologs; CRL: Custom Repeat Library; KPP: kolavenyl diphosphate; NCBI: National Center for Biotechnology Information; RNA-seq: RNA-sequencing; SRA: Sequence Read Archive; TPM: transcripts per million; TPS: Terpene synthase

Competing interests

The authors declare no competing interests.

Funding

Funds for this study were provided by a grant to C.R.B., D.S., and P.S. from the National Science Foundation Plant Genome Research Program (IOS-1444499), a grant to C.R.B. and Bj.H. from the Michigan State University Strategic Partnership Grants Program, and from Hatch funds to C.R.B. (MICL02431). Bj.H. gratefully acknowledges the U.S. Department of Energy-Great Lakes Bioenergy Research Center Cooperative Agreement DE-FC02-07ER64494 and DE-SC0018409, the Michigan State University Strategic Partnership Grant program “Plant-inspired Chemical Diversity”, startup funding from the Department of Molecular Biology and Biochemistry, Michigan State University, and support from Michigan State University AgBioResearch (MICL02454).

Author contributions

JPH performed the genome assembly, annotation, and comparative analyses. BV and JW isolated nucleic acids and performed quality assessments. HW and JJ performed the chromosome counting. GG and TJK performed the whole-genome duplication analyses. BH, ERL, DS, PS, and CRB designed the experiments. WWB performed the phylogenetic analyses and built the CamTPS repository. ERL identified and functionally characterized the terpene synthases. CRB, JH, GG, and BH wrote the manuscript. All authors approved the final manuscript.

Acknowledgements

None

Figure Legends

Figure 1. A. *Callicarpa americana* L. (beautyberry) plant with fruit. B. Somatic chromosome squash of a root tip cell of *C. americana* with 2*n* = 34. Bar = 10 μm.

Figure 2. Comparative genome analyses with *Callicarpa americana* L. A. Orthologous groups between *C. americana* and five other angiosperms (*Amborella trichopoda* Baill [34] (*Amborella*), *Oryza sativa* L. (Rice, MSU v7), *Arabidopsis thaliana* (L.) Heynh (Araport 11;[35]), and two Lamiaceae species: *Tectona grandis* (teak, Tectonoideae; [6]) and *Salvia splendens* Ker Gawl. (scarlet sage; Nepetoideae [4])). B. Syntenic relationship between *T. grandis* and *C. americana*. The upper row shows the 17 *C. americana* pseudomolecules with syntenic alignments to the 19 *T. grandis* pseudomolecules.

Figure 3. Whole-genome duplication (WGD) events inferred from the *Callicarpa americana* L. (beautyberry) genome. Gaussian distributions produced by mixture models are shown as overlays on the K_S distribution, with red or blue color-coded peaks representing putative WGD events that were either corroborated or not corroborated, respectively, by a SiZer analysis

([41]; lower plot). The SiZer plot shows significant increases (blue) or decreases (red), or no significant changes (pink) across the K_S distribution at various (log transformed) bandwidths to distinguish true data features from noise.

Figure 4. Phylogenetic analysis and classification of the *Callicarpa americana* terpene synthase family [6]. Shown are the distinct terpene synthase gene families TPS-a to TPS-g. Highlighted in boxes are TPSs clustered in proximity on the genomic pseudomolecules. *C. americana* TPSs in bold; red stars, functionally characterized members of the TPS-c subfamily; dots on branches indicate bootstrap support equal to or greater than 80%. The phylogeny was rooted with the bifunctional *Physcomitrella patens* (moss) PpCPS/EKS. Annotation of *C. americana* and reference TPSs are given in Tables S7 and S8.

Figure 5. Tissue-specific expression of the *Callicarpa americana* L. terpene synthase gene family. Expression is in transcripts per million. TPS subfamily classification of *C. ameri*cana TPSs is given in Table S8. Red stars, functionally characterized members of the TPS-c subfamily.

Figure 6. Activities of functionally characterized *Callicarpa americana* L. TPS-c. Dotted arrows indicate putative further functionalization by class I diTPS and cytochromes P450 to diterpene products accumulating in *C. americana*.

Tables

Table 1. Metrics of final *Callicarpa americana* L. genome assembly.

Feature	Metric
Canu-derived Contigs	
N50 contig Size (bp)	7,510,543
Total Assembly Size (bp)	506,106,333
Number of Contigs	965
Maximum Contig Length (bp)	18,804,173
Minimum Contig Length (bp)	1,028
Hi-C Scaffolded Assembly	
N50 Scaffold Size (bp)	29,054,287
Total Assembly Size (bp)	506,362,408
Number of Scaffolds	328
Maximum Scaffold Length (bp)	39,429,362
Minimum Scaffold Length (bp)	1,028
Number of Pseudomolecules	17
Total Pseudomolecule Size (bp)	493,744,786
Number of Unanchored Scaffolds	311
Total Unanchored Scaffolds Size (bp)	12,617,622
Pseudomolecules	
Chr01 (bp)	39,429,362
Chr02 (bp)	32,953,817
Chr03 (bp)	32,428,638
Chr04 (bp)	32,381,817
Chr05 (bp)	31,681,419
Chr06 (bp)	31,029,626
Chr07 (bp)	29,370,463
Chr08 (bp)	29,054,287
Chr09 (bp)	28,692,425
Chr10 (bp)	28,677,202
Chr11 (bp)	28,224,296
Chr12 (bp)	27,270,263
Chr13 (bp)	27,197,714
Chr14 (bp)	27,108,606
Chr15 (bp)	23,772,120
Chr16 (bp)	22,946,943
Chr17 (bp)	21,525,788

Table 2. *Callicarpa americana* L. gene annotation summary

	Working Model Set	High-Confidence Model Set
Number of Gene Models	67,826	62,993
Number of Loci	36,480	32,164
Maximum Transcript Length (bp)	16,862	15,978
Maximum CDS Length (bp)	16,269	15,294
Average Transcript Length (bp)	2,004.6	2,096.2
Average CDS Length (bp)	1,305.6	1,355.2
Average Exon Length (bp)	323.5	323.8
Average Intron Length (bp)	500.4	497.4
Single Exon Transcripts	18,140	14,496

Table 3. Physically clustered specialized metabolite biosynthetic pathways in *Callicarpa americana* L. as identified by PlantiSMASH.

Type	Number
Alkaloid	5
Lignan	2
Lignan-Saccharide	1
Polyketide	3
Saccharide	15
Saccharide-Polyketide	1
Saccharide-Terpene	2
Terpene	9
Terpene-Polyketide	1
Uncharacterized	8
Total	47

Supplementary text

Phylogenetic tree

C. americana TPSs were identified by Blastp (v. 2.2.31+) [54] using a set of reference terpene synthases against the gene models. Hits with less than 350 amino acids or less than 30% identity to the reference sequences were filtered out. Reference sequences for functionally characterized TPS are given in table S8. Sequences were aligned using the MUSCLE program from MEGA [55]. A maximum likelihood tree was generated using Jones-Taylor-Thornton model with MEGA X [55] with 1,000 bootstrap repetitions. The tree figure was generated using FigTree v1.4.3 [56].

Heatmap generation

Gene expression heat maps were generated by using ClustVis web tool [57], using TPM values of the TPS gene expression in different tissues of *C. americana* (Table S4).

diTPS cloning

From RNA (extracted as detailed in the main text), cDNA was prepared using the Invitrogen SuperScript™ IV One-Step RT-PCR System. After cloning into pJET1.2 (Thermo Fisher Scientific, Waltham, MA, USA), TPSs were transferred into pEAQ-HT [58] using In-Fusion® HD Cloning Plus (Takara Bio, California, USA) for transient expression in *Nicotiana benthamiana*.

Oligonucleotides for cloning of *C. americana* TPS candidates (given in 5’ to 3’):

Cam_TPS1_For	AAGCTCTCCTCTGCCGTTAAA
Cam_TPS1_Rev	CACAACTTTCATGTACATACTATACC
Cam_TPS2_For	ATGTCATTTGCTTCCCATGCCA
Cam_TPS2_Rev	CAGAACAGGAAGTGTAACTCTACC
Cam_TPS3_For	TCCAATCACACCAACGTTAATTTC
Cam_TPS3_Rev	GATTTACATGTACGTACATGGTCAGAG
Cam_TPS6_For	CTTTGCTACACTGCAGACAAC
Cam_TPS6_Rev	AGTTCGACCGAATTGCGGAAACA

Functional characterization of diTPSs by transient expression in *N. benthamiana*

DiTPS candidates and control genes were transiently expressed in *N. benthamiana* leaves as previously described [46]. To increase product accumulation, diTPSs were co-expressed with genes from the upstream pathway providing the substrate, CfDXS and CfGGPPS (Cf, *Coleus forskohlii*) [51, 52]. Five days after infiltration, leaf tissue was extracted over-night in hexane before analyzed by GC-MS.

GC-MS analyses were performed on an Agilent 7890A GC with an Agilent VF-5ms column (30 m x 250 µm x 0.25 µm, with 10m EZ-Guard) and an Agilent 5975C detector. The inlet was set to 275°C splitless injection, He carrier gas with column flow of 1 mL/min. The oven program was 40°C hold 1 min, 40 °C/min to 200°C and hold 4.5 min, 20°C/min to 240°C, 10°C/min to 280°C, 40°C/min to 320°C hold 3 min. All analyses were done in duplicate. Original raw GC-MS data were deposited to Zenodo (doi.org/10.5281/zenodo.3672159).

References

1. Mint Evolutionary Genomics Consortium. Phylogenomic Mining of the Mints Reveals Multiple Mechanisms Contributing to the Evolution of Chemical Diversity in Lamiaceae. Mol Plant. 2018;11 8:1084-96. doi:10.1016/j.molp.2018.06.002.
2. Xu H, Song J, Luo H, Zhang Y, Li Q, Zhu Y, et al. Analysis of the genome sequence of the medicinal plant *Salvia miltiorrhiza*. Mol Plant. 2016;9 6:949-52. doi:10.1016/j.molp.2016.03.010.
3. Malli RPN, Adal AM, Sarker LS, Liang P and Mahmoud SS. *De novo* sequencing of the *Lavandula angustifolia* genome reveals highly duplicated and optimized features for essential oil production. Planta. 2019;249 1:251-6. doi:10.1007/s00425-018-3012-9.
4. Dong AX, Xin HB, Li ZJ, Liu H, Sun YQ, Nie S, et al. High-quality assembly of the reference genome for scarlet sage, *Salvia splendens*, an economically important ornamental plant. Gigascience. 2018;7 7 doi:10.1093/gigascience/giy068.
5. Zhao Q, Yang J, Cui MY, Liu J, Fang Y, Yan M, et al. The Reference Genome Sequence of *Scutellaria baicalensis* Provides Insights into the Evolution of Wogonin Biosynthesis. Mol Plant. 2019;12 7:935-50. doi:10.1016/j.molp.2019.04.002.
6. Zhao D, Hamilton JP, Bhat WW, Johnson SR, Godden GT, Kinser TJ, et al. A chromosomal-scale genome assembly of *Tectona grandis* reveals the importance of tandem gene duplication and enables discovery of genes in natural product biosynthetic pathways. Gigascience. 2019;8(3):giz005. doi:10.1093/gigascience/giz005.
7. Krajick K. Medical entomology. Keeping the bugs at bay. Science. 2006;313 5783:36-8. doi:10.1126/science.313.5783.36.
8. Cantrell CL, Klun, J.A. Callicarpenal and Intermedeol: Two natural arthropod feeding deterrent and repellent compounds Iidentified from the southern folk remedy plant, *Callicarpa americana*. Recent Developments in Invertrebrate Repellents. Washington DC: American Chemical Society; 2011.

9. Cantrell CL, Klun JA, Bryson CT, Kobaisy M and Duke SO. Isolation and identification of mosquito bite deterrent terpenoids from leaves of American (*Callicarpa americana*) and Japanese (*Callicarpa japonica*) beautyberry. J Agric Food Chem. 2005;53 15:5948-53. doi:10.1021/jf0509308.
10. Doyle JJ, Doyle, J.L. A rapid DNA isolation procedure for small quantities of fresh leaf tissue. Phytochemical Bulletin. 1987;19:11-5.
11. Davidson RM, Gowda M, Moghe G, Lin H, Vaillancourt B, Shiu SH, et al. Comparative transcriptomics of three Poaceae species reveals patterns of gene expression evolution. Plant Journal. 2012;71 3:492-502. doi:10.1111/j.1365-313X.2012.05005.x.
12. Koren S, Walenz BP, Berlin K, Miller JR, Bergman NH and Phillippy AM. Canu: scalable and accurate long-read assembly via adaptive k-mer weighting and repeat separation. Genome Res. 2017;27 5:722-36. doi:10.1101/gr.215087.116.
13. PacBio® variant and consensus caller. https://github.com/PacificBiosciences/GenomicConsensus. Accessed May 2018.
14. Walker BJ, Abeel T, Shea T, Priest M, Abouelliel A, Sakthikumar S, et al. Pilon: an integrated tool for comprehensive microbial variant detection and genome assembly improvement. PLoS One. 2014;9 11:e112963. doi:10.1371/journal.pone.0112963.
15. Martin M. Cutadapt removes adapter sequences from high-throughput sequencing reads. EMBnetjournal. 2011;17 1 doi:http://dx.doi.org/10.14806/ej.17.1.200.
16. Li H. Aligning sequence reads, clone sequences and assembly contigs with BWA-MEM. arXiv. 2013;1303.3997v2.
17. Braz GT, He L, Zhao H, Zhang T, Semrau K, Rouillard JM, et al. Comparative oligo-FISH Mapping: An Efficient and Powerful Methodology to Reveal Karyotypic and Chromosomal Evolution. Genetics. 2018;208:513-23. doi:10.1534/genetics.117.300344.
18. Peichel CL, Ross JA, Matson CK, Dickson M, Grimwood J, Schmutz J, et al. The master sex-determination locus in threespine sticklebacks is on a nascent Y chromosome. Curr Biol. 2004;14 16:1416-24.
19. Kim D, Langmead B and Salzberg SL. HISAT: a fast spliced aligner with low memory requirements. Nature Methods. 2015;12 4:357-60. doi:10.1038/nmeth.3317.

20. Simao FA, Waterhouse RM, Ioannidis P, Kriventseva EV and Zdobnov EM. BUSCO: assessing genome assembly and annotation completeness with single-copy orthologs. Bioinformatics. 2015;31 19:3210-2. doi:10.1093/bioinformatics/btv351.

21. Smit A, Hubley, R.: RepeatModeler. http://www.repeatmasker.org/. Accessed October 2018.

22. Campbell MS, Law M, Holt C, Stein JC, Moghe GD, Hufnagel DE, et al. MAKER-P: a tool kit for the rapid creation, management, and quality control of plant genome annotations. Plant Physiol. 2014;164 2:513-24. doi:10.1104/pp.113.230144.

23. Jurka J, Kapitonov VV, Pavlicek A, Klonowski P, Kohany O and Walichiewicz J. Repbase Update, a database of eukaryotic repetitive elements. Cytogenet Genome Res. 2005;110 1-4:462-7.

24. Chen N. Using RepeatMasker to identify repetitive elements in genomic sequences. Curr Protoc Bioinformatics. 2004;Chapter 4:Unit 4 10.

25. Kim D, Paggi JM, Park C, Bennett C and Salzberg SL. Graph-based genome alignment and genotyping with HISAT2 and HISAT-genotype. Nat Biotechnol. 2019;37 8:907-15. doi:10.1038/s41587-019-0201-4.

26. Grabherr MG, Haas BJ, Yassour M, Levin JZ, Thompson DA, Amit I, et al. Full-length transcriptome assembly from RNA-Seq data without a reference genome. Nature Biotechnology. 2011;29 7:644-52. doi:10.1038/nbt.1883.

27. Haas BJ, Delcher AL, Mount SM, Wortman JR, Smith RK, Jr., Hannick LI, et al. Improving the Arabidopsis genome annotation using maximal transcript alignment assemblies. Nucleic Acids Res. 2003;31 19:5654-66.

28. PASA2. http://pasapipeline.github.io/. Accessed October 2018.

29. Campbell MA, Haas BJ, Hamilton JP, Mount SM and Buell CR. Comprehensive analysis of alternative splicing in rice and comparative analyses with Arabidopsis. BMC Genomics. 2006;7:327.

30. Bray NL, Pimentel H, Melsted P and Pachter L. Near-optimal probabilistic RNA-seq quantification. Nat Biotechnol. 2016;34 5:525-7. doi:10.1038/nbt.3519.

31. The Arabidopsis Information Resource. Arabidopsis.org. Accessed October 2018.

32. Finn RD, Coggill P, Eberhardt RY, Eddy SR, Mistry J, Mitchell AL, et al. The Pfam protein families database: towards a more sustainable future. Nucleic Acids Res. 2016;44 D1:D279-85. doi:10.1093/nar/gkv1344.
33. Emms DM, Kelly, S. OrthoFinder2: fast and accurate phylogenomic orthology analysis from gene sequences. bioRxiv. 2018; https://doi.org/10.1101/466201.
34. Amborella Genome Project. The Amborella genome and the evolution of flowering plants. Science. 2013;342 6165:1241089. doi:10.1126/science.1241089.
35. Cheng C-Y, Krishnakumar V, Chan AP, Thibaud-Nissen F, Schobel S and Town CD. Araport11: a complete reannotation of the Arabidopsis thaliana reference genome. The Plant Journal. 2017;89 4:789-804. doi:10.1111/tpj.13415.
36. Jones P, Binns D, Chang H-Y, Fraser M, Li W, McAnulla C, et al. InterProScan 5: genome-scale protein function classification. Bioinformatics. 2014;30 9:1236-40. doi:10.1093/bioinformatics/btu031.
37. Alexa A, Rahnenfuhrer, J. topGO: Enrichment Analysis for Gene Ontology. R package version 2.38.1. 2019.
38. Paterson AH, Li J and Wang Y. MCScanX-transposed: detecting transposed gene duplications based on multiple colinearity scans. Bioinformatics. 2013;29 11:1458-60. doi:10.1093/bioinformatics/btt150.
39. Godden GT, Kinser TJ, Soltis PS and Soltis DE. Phylotranscriptomic Analyses Reveal Asymmetrical Gene Duplication Dynamics and Signatures of Ancient Polyploidy in Mints. Genome Biol Evol. 2019;11 12:3393-408. doi:10.1093/gbe/evz239.
40. Barker MS, Dlugosch KM, Dinh L, Challa RS, Kane NC, King MG, et al. EvoPipes.net: Bioinformatic Tools for Ecological and Evolutionary Genomics. Evol Bioinform Online. 2010;6:143-9. doi:10.4137/EBO.S5861.
41. Chaudhuri P, Marron, J. S. SiZer for Exploration of Structures in Curves. J Am Stat Assoc 1999;94:807.
42. Jones WP and Kinghorn AD. Biologically active natural products of the genus *Callicarpa*. *Current Bioactive Compounds*. 2008, p. 15-32.

43. Hansen NL, Heskes AM, Hamberger B, Olsen CE, Hallström BM, Andersen-Ranberg J, et al. The terpene synthase gene family in *Tripterygium wilfordii* harbors a labdane-type diterpene synthase among the monoterpene synthase TPS-b subfamily. *The Plant Journal*. 2017;89:429-41.

44. Chen X, Berim A, Dayan FE and Gang DR. A (–)-kolavenyl diphosphate synthase catalyzes the first step of salvinorin A biosynthesis in *Salvia divinorum*. *Journal of Experimental Botany*. 2017, p. 1109-22.

45. Pelot KA, Mitchell R, Kwon M, Hagelthorn DM, Wardman JF, Chiang A, et al. Biosynthesis of the psychotropic plant diterpene salvinorin A: Discovery and characterization of the *Salvia divinorum* clerodienyl diphosphate synthase. *The Plant Journal*. 2017; 89:885-897.

46. Johnson SR, Bhat WW, Bibik J, Turmo A, Hamberger B, Mint Evolutionary Genomics Consortium, et al. A database-driven approach identifies additional diterpene synthase activities in the mint family (Lamiaceae). J Biol Chem. 2018;25:1349-62. doi:10.1074/jbc.RA118.006025.

47. Nutzmann HW, Huang A and Osbourn A. Plant metabolic clusters - from genetics to genomics. New Phytol. 2016;211 3:771-89. doi:10.1111/nph.13981.

48. Liu Z, Suarez Duran HG, Harnvanichvech Y, Stephenson MJ, Schranz ME, Nelson D, et al. Drivers of metabolic diversification: how dynamic genomic neighbourhoods generate new biosynthetic pathways in the Brassicaceae. New Phytol. 2019; doi:10.1111/nph.16338.

49. Kautsar SA, Suarez Duran HG, Blin K, Osbourn A and Medema MH. plantiSMASH: automated identification, annotation and expression analysis of plant biosynthetic gene clusters. Nucleic Acids Res. 2017;45 W1:W55-W63. doi:10.1093/nar/gkx305.

50. Hamberger B and Bak S. Plant P450s as versatile drivers for evolution of species-specific chemical diversity. *Philosophical Transactions of the Royal Society B: Biological Sciences*. 2013.

51. Andersen-Ranberg J, Kongstad KT, Nielsen MT, Jensen NB, Pateraki I, Bach SS, et al. Expanding the landscape of diterpene structural diversity through stereochemically

controlled combinatorial biosynthesis. Angewandte Chemie - International Edition. 2016;55 6:2142-6. doi:10.1002/anie.201510650.

52. Pateraki I, Andersen-Ranberg J, Hamberger B, Heskes AM, Martens HJ, Zerbe P, et al. Manoyl oxide (13R), the biosynthetic precursor of forskolin, is synthesized in specialized root cork cells in *Coleus forskohlii*. Plant Physiology. 2014;164 3:1222-36. doi:10.1104/pp.113.228429.

53. Harris LJ, Saparno A, Johnston A, Prisic S, Xu M, Allard S, et al. The Maize An2 Gene is Induced by Fusarium Attack and encodes an *ent*-Copalyl Diphosphate Synthase. *Plant Molecular Biology*. 2005;59:881-94.

54. Camacho C, Coulouris G, Avagyan V, Ma N, Papadopoulos J, Bealer K, et al. BLAST+: architecture and applications. BMC Bioinformatics. 2009;10:421. doi:10.1186/1471-2105-10-421.

55. Kumar S, Stecher G and Tamura K. MEGA7: Molecular Evolutionary Genetics Analysis Version 7.0 for Bigger Datasets. Mol Biol Evol. 2016;33 7:1870-4. doi:10.1093/molbev/msw054.

56. Figtree: Figtree. http://tree.bio.ed.ac.uk/software/figtree/.Accessed December 2019

57. ClustVis Web tool: https://biit.cs.ut.ee/clustvis/. Accessed December 2019.

58. Sainsbury F, Thuenemann EC and Lomonossoff GP. pEAQ: versatile expression vectors for easy and quick transient expression of heterologous proteins in plants. Plant Biotechnol J. 2009;7:682-93. doi:10.1111/j.1467-7652.2009.00434.x.

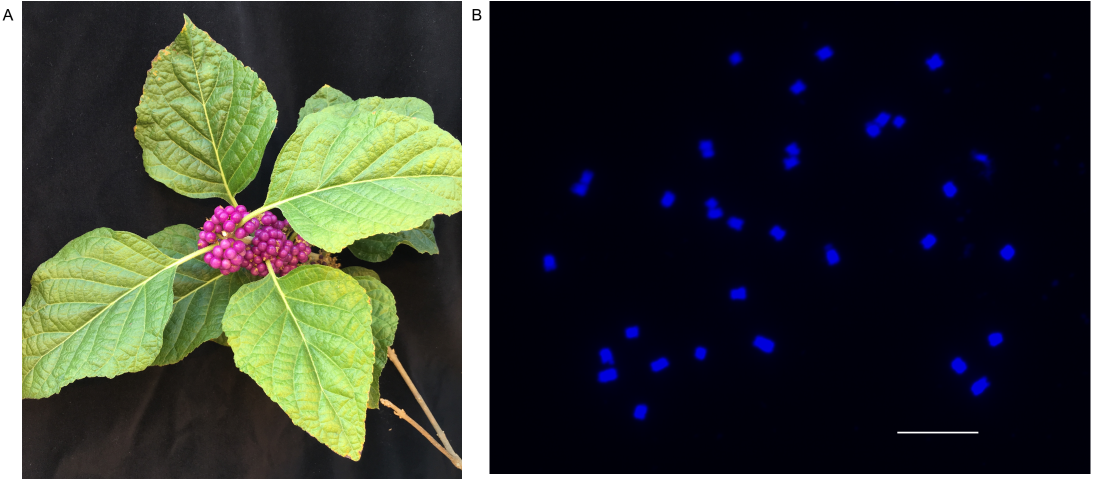
A
B

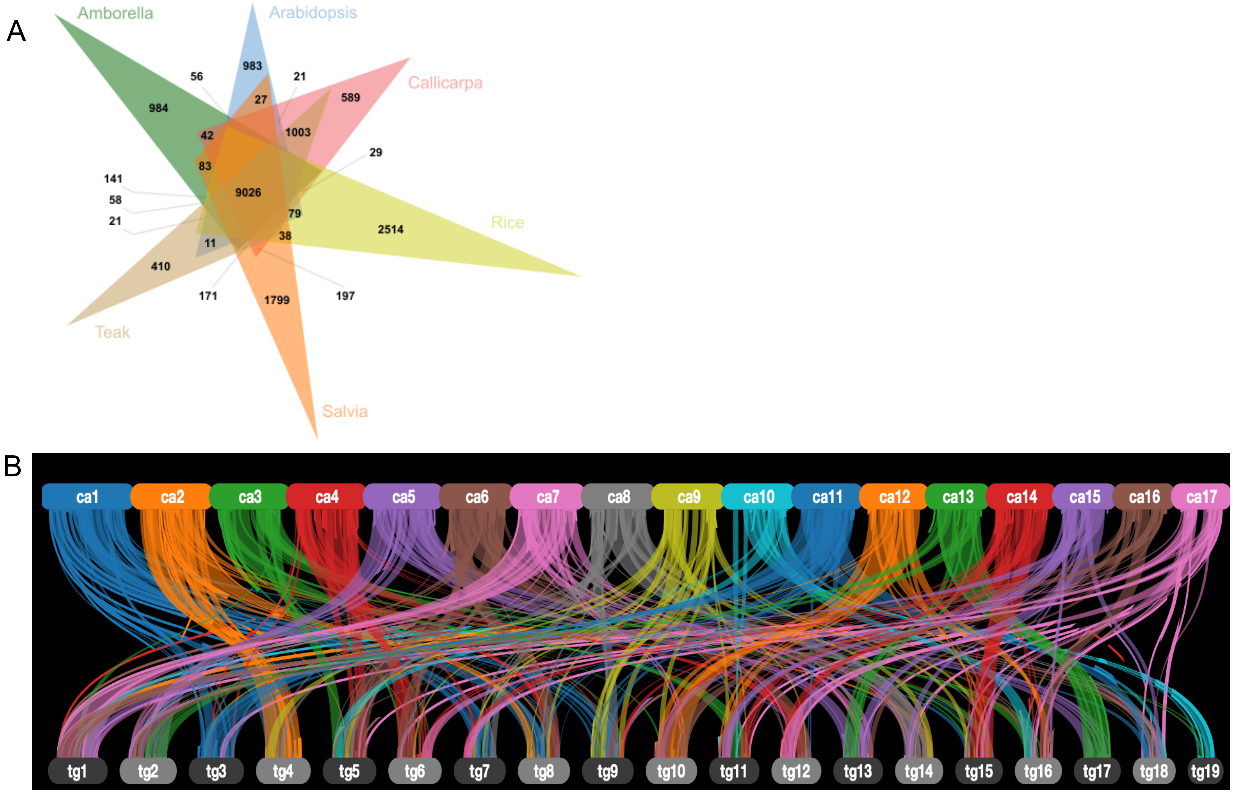
A
Amborella
Arabidopsis
Callicarpa
Rice
Teak
Salvia
983
56
21
984
27
589
42
1003
29
83
141
9026
58
79
21
2514
38
11
410
171
1799
197
B
ca1 ca2 ca3 ca4 ca5 ca6 ca7 ca8 ca9 ca10 ca11 ca12 ca13 ca14 ca15 ca16 ca17
tg1 tg2 tg3 tg4 tg5 tg6 tg7 tg8 tg9 tg10 tg11 tg12 tg13 tg14 tg15 tg16 tg17 tg18 tg19

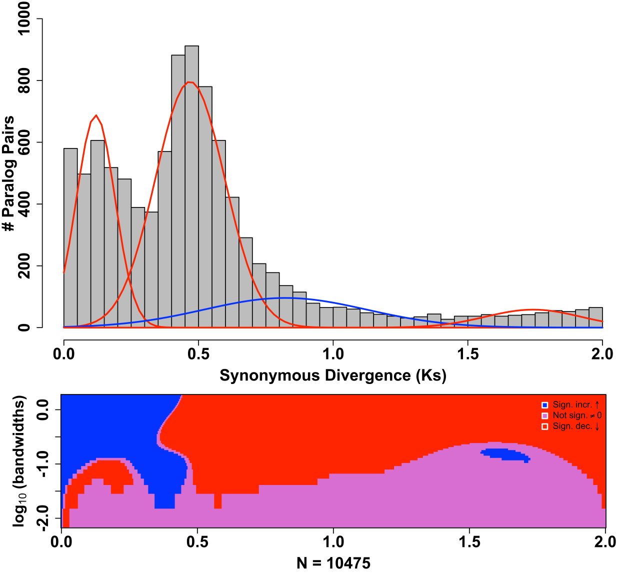

Paralog Pairs
0
200
400
600
800
1000
0.0
0.5
1.0
1.5
2.0
Synonymous Divergence (Ks)
log10 (bandwidths)
0.0
-1.0
-2.0
Sign. incr. ↑
Not sign. ≠ 0
Sign. dec. ↓
N = 10475

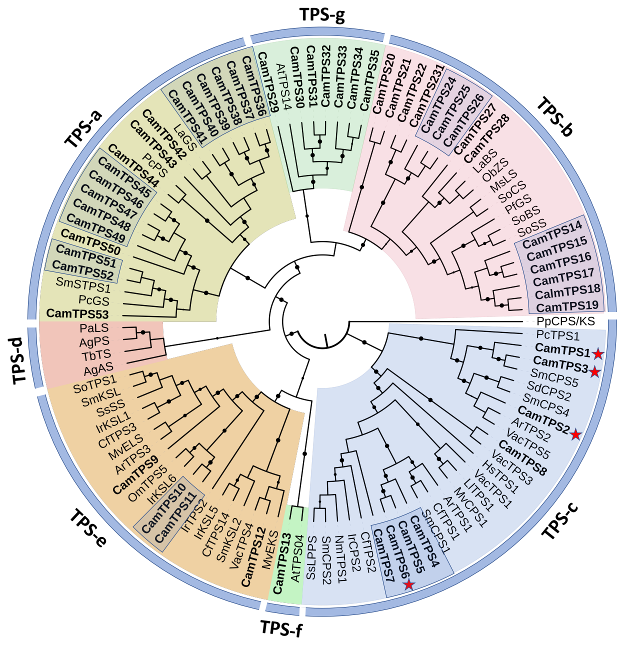

TPS-g
TPS-b
TPS-c
TPS-f
TPS-e
TPS-d
TPS-a
CamTPS29
AtTPS14
CamTPS30
CamTPS31
CamTPS32
CamTPS33
CamTPS34
CamTPS35
CamTPS20
CamTPS21
CamTPS22
CamTPS231
CamTPS24
CamTPS25
CamTPS26
CamTPS27
CamTPS28
LaBS
ObZS
MsLS
SoCS
PfGS
SoBS
SoSS
CamTPS14
CamTPS15
CamTPS16
CamTPS17
CalmTPS18
CamTPS19
PpCPS/KS
PcTPS1
CamTPS1
CamTPS3
SmCPS5
SdCPS2
SmCPS4
CamTPS2
ArTPS2
VacTPS5
CamTPS8
VacTPS3
HsTPS1
VacTPS1
LlTPS1
MvCPS1
ArTPS1
CfTPS1
SmCPS1
CamTPS4
CamTPS5
CamTPS6
CamTPS7
CfTPS2
IrCPS2
NmTPS1
SmCPS2
SsLPPS
AtTPS04
CamTPS13
MvEKS
CamTPS12
VacTPS4
SmKSL2
CfTPS14
IrKSL5
IrTPS2
CamTPS11
CamTPS10
IrKSL6
OmTPS5
CamTPS9
ArTPS3
MvELS
CfTPS3
IrKSL1
SsSS
SmKSL
SoTPS1
AgAS
TbTS
AgPS
PaLS
CamTPS53
PcGS
SmSTPS1
CamTPS52
CamTPS51
CamTPS50
CamTPS49
CamTPS48
CamTPS47
CamTPS46
CamTPS45
CamTPS44
PcPS
CamTPS43
CamTPS42
LaGS
CamTPS41
CamTPS40
CamTPS39
CamTPS38
CamTPS37
CamTPS36

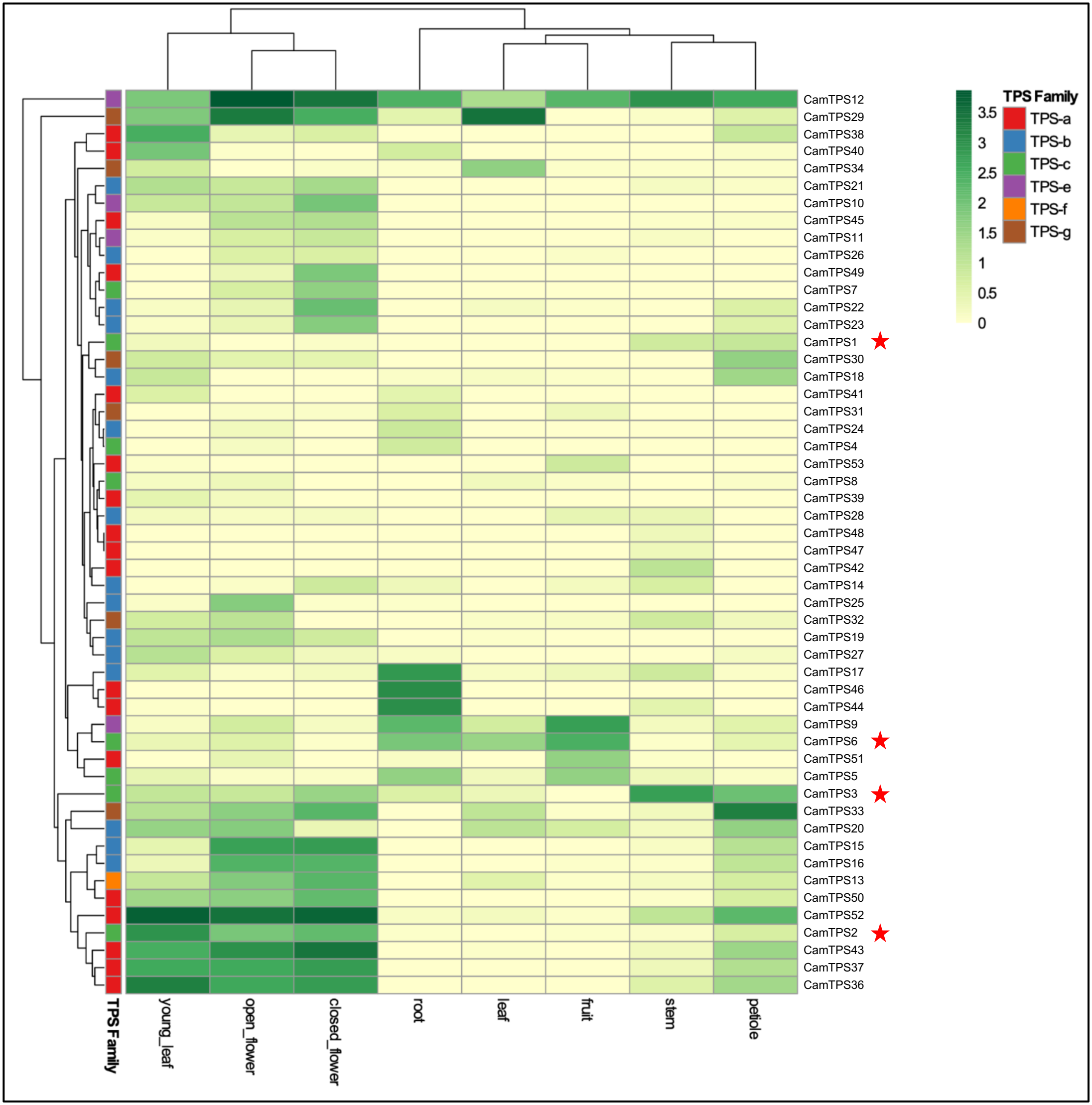

CamTPS12
CamTPS29
CamTPS38
CamTPS40
CamTPS34
CamTPS21
CamTPS10
CamTPS45
CamTPS11
CamTPS26
CamTPS49
CamTPS7
CamTPS22
CamTPS23
CamTPS1
CamTPS30
CamTPS18
CamTPS41
CamTPS31
CamTPS24
CamTPS4
CamTPS53
CamTPS8
CamTPS39
CamTPS28
CamTPS48
CamTPS47
CamTPS42
CamTPS14
CamTPS25
CamTPS32
CamTPS19
CamTPS27
CamTPS17
CamTPS46
CamTPS44
CamTPS9
CamTPS6
CamTPS51
CamTPS5
CamTPS3
CamTPS33
CamTPS20
CamTPS15
CamTPS16
CamTPS13
CamTPS50
CamTPS52
CamTPS2
CamTPS43
CamTPS37
CamTPS36
TPS Family
young_leaf
open_flower
closed_flower
root
leaf
fruit
stem
petiole
3.5
3
2.5
2
1.5
1
0.5
0
TPS Family
TPS-a
TPS-b
TPS-c
TPS-e
TPS-f
TPS-g

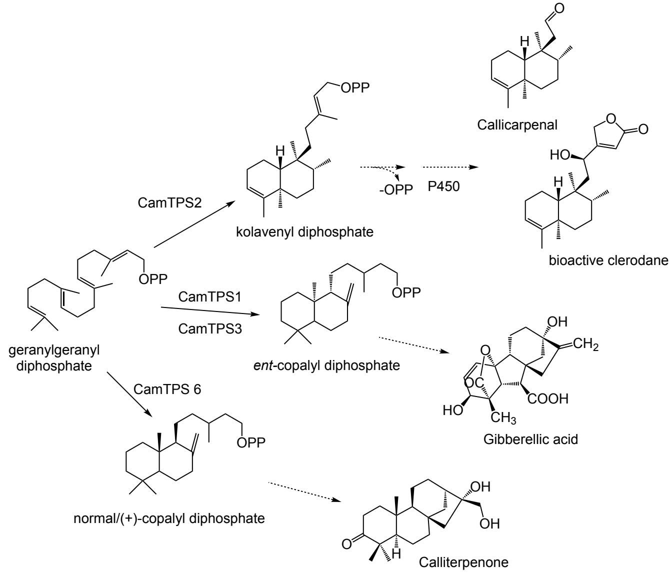
Callicarpenal
bioactive clerodane
kolavenyl diphosphate
CamTPS2
-OPP
P450
geranylgeranyl diphosphate
CamTPS1
CamTPS3
ent-copalyl diphosphate
Gibberellic acid
CamTPS 6
normal/(+)-copalyl diphosphate
Calliterpenone
OPP
HO
OH
COOH
OH

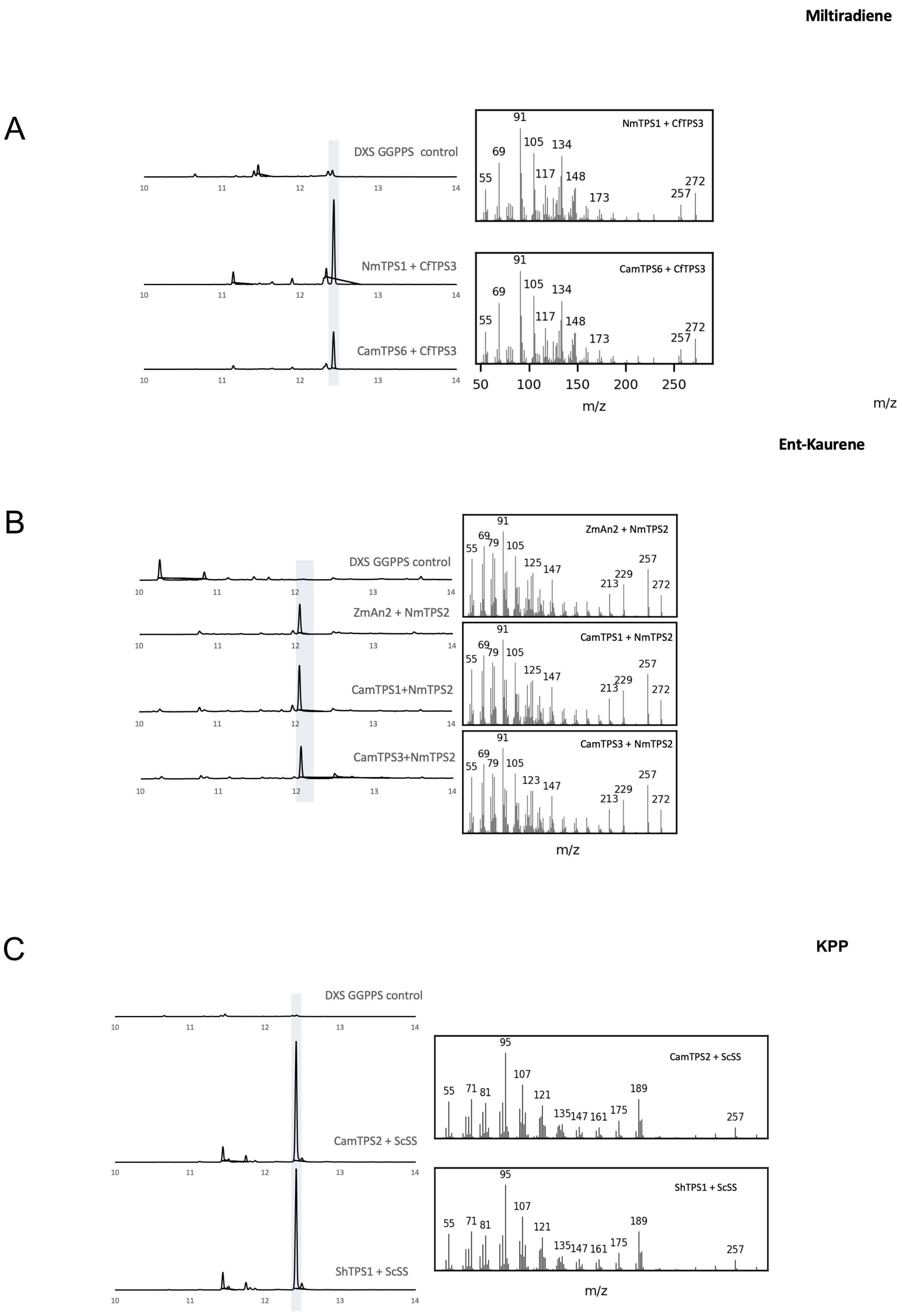

Miltiradiene
A
DXS GGPPS control
NmTPS1 + CfTPS3
CamTPS6 + CfTPS3
10
11
12
13
14
NmTPS1 + CfTPS3
91
69
105
134
55
117
148
173
257
272
CamTPS6 + CfTPS3
91
69
105
134
55
117
148
173
257
272
50
100
150
200
250
m/z
m/z
Ent-Kaurene
B
DXS GGPPS control
ZmAn2 + NmTPS2
CamTPS1+NmTPS2
CamTPS3+NmTPS2
ZmAn2 + NmTPS2
91
69
79
55
105
125
147
213
229
257
272
CamTPS1 + NmTPS2
91
69
79
55
105
125
147
213
229
257
272
CamTPS3 + NmTPS2
91
69
79
55
105
123
147
213
229
257
272
m/z
C
KPP
DXS GGPPS control
CamTPS2 + ScSS
ShTPS1 + ScSS
CamTPS2 + ScSS
95
107
55
71
81
121
135
147
161
175
189
257
ShTPS1 + ScSS
95
107
55
71
81
121
135
147
161
175
189
257
m/z

Table S1: RNA-Seq, whole-genome shotgun, and Hi-C libraries used in this study.

Description	SRA BioSample	SRA Run ID	Type of library	Total Reads
Mature leaf total RNA	SAMN11461722	SRR8927028	RNA-Seq	64,758,847
Young leaf total RNA	SAMN11461723	SRR8927027	RNA-Seq	64,102,615
Stem total RNA	SAMN11461724	SRR8927030	RNA-Seq	63,334,609
Petiole total RNA	SAMN11461725	SRR8927029	RNA-Seq	64,505,790
Root total RNA	SAMN11461726	SRR8927024	RNA-Seq	34,049,452
Open flower total RNA	SAMN11461727	SRR8927023	RNA-Seq	53,825,309
Closed flower total RNA	SAMN11461728	SRR8927026	RNA-Seq	43,872,498
Whole fruit total RNA	SAMN11461729	SRR8927025	RNA-Seq	52,872,604
Leaf tissue for Hi-C	SAMN11463016	SRR8928348	Hi-C	368,401,363
Young leaf DNA	SAMN11462982	SRR8927066	WGS	135,334,284
Leaf DNA for PacBio sequencing	SAMN11462983	SRR8932629	WGS (PacBio)	4,259,656
Leaf DNA for PacBio sequencing	SAMN11462983	SRR8932628	WGS (PacBio)	578,595

Table S2. PacBio flow cells used in this study.

Flow Cell	SRA Run ID	Number of Reads	Total Bases (bp)	Number of Reads >= 1 kb	Total Bases (bp) of Reads >= 1kb
m54193_180208_222314	SRR8932629	792,206	6,058,752,842	638,540	5,988,509,957
m54193_180214_024450	SRR8932629	741,027	5,975,777,488	600,251	5,911,727,105
m54193_180216_214838	SRR8932629	507,161	5,746,584,671	438,062	5,713,267,589
m54193_180217_075436	SRR8932629	515,407	5,723,396,037	448,759	5,691,420,913
m54193_180217_180425	SRR8932629	523,011	5,317,858,636	452,289	5,283,423,048
m54193_180218_041402	SRR8932629	515,839	5,018,010,038	440,138	4,981,656,768
m54193_180222_221210	SRR8932629	328,825	2,975,854,654	283,371	2,953,851,725
m54193_180223_081748	SRR8932629	336,180	3,134,210,757	292,354	3,112,640,936
m54193_180305_222630	SRR8932628	132,510	1,417,617,355	117,082	1,409,600,690
m54193_180225_003437	SRR8932628	336,689	2,904,575,688	288,854	2,881,496,621
m54193_180307_144557	SRR8932628	109,396	1,112,574,235	96,016	1,105,673,125
Total		4,838,251	45,385,212,401	4,095,716	45,033,268,477

Table S3. *Callicarpa americana* RNA-seq alignment and genome-guided assembly transcript metrics.

Library Name	SRA Run ID	Tissue	Read Pairs*	Cleaned Read Pairs	HISAT2 Overall Alignment Rate	Number of Genome Guided Transcripts
CAR_AF	SRR8927028	Mature leaf	64,758,847	64,279,081	96.31%	78,678
CAR_AG	SRR8927027	Young leaf	64,102,615	63,521,728	96.37%	82,706
CAR_AH	SRR8927030	Stem	63,334,609	62,597,824	96.47%	85,654
CAR_AI	SRR8927029	Petiole	64,505,790	63,950,337	96.16%	85,962
CAR_AJ	SRR8927024	Root	34,049,452	33,167,513	96.78%	58,309
CAR_AK	SRR8927023	Open flower	53,825,309	53,241,605	94.17%	83,248
CAR_AL	SRR8927026	Closed flower	43,872,498	43,482,707	95.70%	80,546
CAR_AM	SRR8927025	Whole fruit	52,872,604	52,732,153	96.31%	77,273

*All reads are 150 nt paired end

96.03%

Table S4. Expression abundances of *Callicarpa americana* genes.

Too big to include as a PDF; provided as a separate excel file

Table S5. Gene ontology enrichment analyses of Lamiaceae specific genes.

Gene Ontology	Term	Annotation	Significant	Expected	Classic Fisher	p-value
Biological Process	GO:0048544	recognition of pollen	156	115	23.23	< 1e-30
Biological Process	GO:0006468	protein phosphorylation	1494	434	222.42	< 1e-30
Biological Process	GO:0006952	defense response	102	42	15.19	1.40E-08
Biological Process	GO:0055114	oxidation-reduction process	1664	317	247.73	6.00E-07
Biological Process	GO:0006420	arginyl-tRNA aminoacylation	8	7	1.19	1.10E-05
Biological Process	GO:0045944	positive regulation of transcription by RNA polymerase II	69	24	10.27	3.10E-05
Biological Process	GO:0006355	regulation of transcription, DNA-templated	962	192	143.22	6.40E-05
Biological Process	GO:0043086	negative regulation of catalytic activity	21	11	3.13	6.40E-05
Biological Process	GO:0009611	response to wounding	25	12	3.72	8.90E-05
Biological Process	GO:0006788	heme oxidation	6	4	0.89	0.0057
Biological Process	GO:0010112	regulation of systemic acquired resistance	7	4	1.04	0.0118
Biological Process	GO:0006887	exocytosis	55	15	8.19	0.0124
Biological Process	GO:0046148	pigment biosynthetic process	47	13	7	0.0171
Biological Process	GO:0045087	innate immune response	9	6	1.34	0.0221
Biological Process	GO:0042545	cell wall modification	82	19	12.21	0.0301
Biological Process	GO:0006508	proteolysis	540	87	80.39	0.0355
Biological Process	GO:0070588	calcium ion transmembrane transport	23	7	3.42	0.0445
Biological Process	GO:0006542	glutamine biosynthetic process	10	4	1.49	0.0487
Molecular Function	GO:0043531	ADP binding	373	322	67.7	< 1e-30
Molecular Function	GO:0004674	protein serine/threonine kinase activity	212	111	38.48	1.10E-29
Molecular Function	GO:0016705	oxidoreductase activity, acting on paired donors, with incorporation or reduction of molecular oxygen	580	196	105.27	7.40E-21
Molecular Function	GO:0005506	iron ion binding	572	189	103.82	2.30E-18
Molecular Function	GO:0020037	heme binding	628	189	113.98	8.00E-14
Molecular Function	GO:0004672	protein kinase activity	1509	434	273.88	8.40E-12
Molecular Function	GO:0004523	RNA-DNA hybrid ribonuclease activity	86	42	15.61	8.10E-11
Molecular Function	GO:0004097	catechol oxidase activity	20	17	3.63	1.60E-10
Molecular Function	GO:0000981	DNA-binding transcription factor activity, RNA polymerase II-specific	14	12	2.54	7.90E-08
Molecular Function	GO:0000987	proximal promoter sequence-specific DNA binding	14	12	2.54	7.90E-08
Molecular Function	GO:0004970	ionotropic glutamate receptor activity	30	18	5.44	4.00E-07
Molecular Function	GO:0016747	transferase activity, transferring acyl groups other than amino-acyl groups	259	71	47.01	4.70E-07
Molecular Function	GO:0046910	pectinesterase inhibitor activity	15	11	2.72	4.60E-06
Molecular Function	GO:0030247	polysaccharide binding	81	32	14.7	5.30E-06
Molecular Function	GO:0016758	transferase activity, transferring hexosyl groups	463	106	84.03	2.20E-05
Molecular Function	GO:0004814	arginine-tRNA ligase activity	8	7	1.45	4.30E-05
Molecular Function	GO:0004867	serine-type endopeptidase inhibitor activity	27	14	4.9	7.60E-05
Molecular Function	GO:0005524	ATP binding	2407	494	436.86	0.00069
Molecular Function	GO:0004857	enzyme inhibitor activity	166	63	30.13	0.00382
Molecular Function	GO:0004869	cysteine-type endopeptidase inhibitor activity	17	8	3.09	0.00596
Molecular Function	GO:0004392	heme oxygenase (decyclizing) activity	6	4	1.09	0.01189
Molecular Function	GO:0004455	ketol-acid reductoisomerase activity	4	3	0.73	0.02065
Molecular Function	GO:0016702	oxidoreductase activity, acting on single donors with incorporation of molecular oxygen, incorporation of two atoms of oxygen	54	16	9.8	0.02692
Molecular Function	GO:0004185	serine-type carboxypeptidase activity	59	17	10.71	0.02997
Molecular Function	GO:0008234	cysteine-type peptidase activity	98	18	17.79	0.03409
Molecular Function	GO:0004534	5'-3' exoribonuclease activity	5	3	0.91	0.04467

Table S6. Gaussian mixture modeling and SiZer results for the *KS* distribution estimated from the genome and transcriptome of *Callicarpa americana* L. Shown here are the number of inferred components, along with their corresponding means (**μ**), mixing proportions (**λ**), and standard deviations (**σ**) estimated by mixtools. The number of components corroborated by a SiZer analysis are indicated in brackets, with corresponding values of **μ**, **λ**, and **σ** from mixture models denoted with an asterisk (*). Transcriptome-based results from Godden et al. 2019.

Data	Components	**μ**	**λ**	**σ**
Genome	4 [3]	0.118*, 0.469*, 0.822, 1.743*	0.259*, 0.536*, 0.152, 0.054*	0.072*, 0.129*, 0.302, 0.176*
Transcriptome	2 [1]	0.513*, 1.345	0.780*, 0.220	0.174*, 0.403

Table S7. Terpene synthases identified in this study.

Gene ID	Genome identifier
CaTPS1	Calam.06G186600.3
CaTPS2	Calam.06G186900.1
CaTPS3	Calam.09G037800.1
CaTPS4	Calam.10G111700.1
CaTPS5	Calam.10G111900.1
CaTPS6	Calam.10G112000.1
CaTPS7	Calam.10G112900.1
CaTPS8	Calam.10G113600.2
CaTPS9	Calam.10G111100.1
CaTPS10	Calam.10G112800.1
CaTPS11	Calam.01G001600.1
CaTPS12	Calam.05G103700.1
CaTPS13	Calam.05G061600.1
CaTPS14	Calam.04G077500.1
CaTPS15	Calam.04G064600.1
CaTPS16	Calam.04G077300.1
CaTPS17	Calam.04G077600.1
CaTPS18	Calam.04G077700.2
CaTPS19	Calam.04G077900.1
CaTPS20	Calam.05G073200.1
CaTPS21	Calam.05G073300.1
CaTPS22	Calam.06G177300.1
CaTPS23	Calam.16G000600.1
CaTPS24	Calam.02G131500.1
CaTPS25	Calam.02G131300.1
CaTPS26	Calam.02G131400.1
CaTPS27	Calam.06G086600.1
CaTPS28	Calam.05G073000.1
CaTPS29	Calam.08G145900.1

CaTPS30	Calam.03G069500.1
CaTPS31	Calam.05G072700.1
CaTPS32	Calam.13G138200.2
CaTPS33	Calam.04G211000.2
CaTPS34	Calam.04G210600.1
CaTPS35	Calam.04G210900.1
CaTPS36	Calam.16G030500.1
CaTPS37	Calam.16G004300.1
CaTPS38	Calam.16G030300.1
CaTPS39	Calam.16G004100.1
CaTPS40	Calam.16G030600.1
CaTPS41	Calam.16G004500.1
CaTPS42	Calam.03G147600.1
CaTPS43	Calam.09G074500.1
CaTPS44	Calam.05G011300.2
CaTPS45	Calam.02G051900.1
CaTPS46	Calam.02G017800.1
CaTPS47	Calam.02G020100.1
CaTPS48	Calam.02G003100.1
CaTPS49	Calam.02G029600.1
CaTPS50	Calam.08G011000.1
CaTPS51	Calam.02G034900.1
CaTPS52	Calam.02G034800.1
CaTPS53	Calam.10G106300.1

Table S8. GenBank protein identifiers of the TPSs used for construction of phylogenetic tree.

Species number	Species	Name	TPS Family	Genbank Peptide
1	*Salvia officinalis*	*SoSS*	Tps-b	O81193
2	*Salvia officinalis*	*SoBS*	Tps-b	O81192
3	*Salvia officinalis*	*SoCS*	Tps-b	O81191
4	*Lavendula angustifolia*	*LaBS*	Tps-b	Q2XSC4
5	*Ocimum basilicum*	*ObZS*	Tps-b	Q5SBP4
6	*Mentha spicata*	*MsLS*	Tps-b	AGN90911
7	*Pogostemon cablin*	*PcGS*	Tps-a	Q49SP5
8	*Salvia miltiorrhiza*	*SmSTPS1*	Tps-a	A0A1W6GW32
9	*Perilla frutescens*	*PfGS*	Tps-b	ABB30218
10	*Lavendula angustifolia*	*LaGS*	Tps-a	AGL98420
11	*Pogostemon cablin*	*PcPS*	Tps-a	AYB35974
12	*Arabidopsis thaliana*	*AtTPS04*	Tps-f	NP_564772
13	*Arabidopsis thaliana*	*AtTPS14*	Tps-g	NP_176361
14	*Physcomitrella patens*	*PpCPS/KS*	Tps-d	XP_024380398
15	*Abies grandis*	*AgAS*	Tps-d	Q38710
16	*Taxus brevifolia*	*TbTS*	Tps-d	Q41594
17	*Picea abies*	*PaLS*	Tps-d	AAS47694
18	*Abies grandis*	*AgPS*	Tps-d	O24475
19	*Salvia sclarea*	*SsSS*	Tps-e	AFU61898
20	*Plectranthus barbatus*	*CfTPS14*	Tps-e	AGN70881
21	*Salvia miltiorrhiza*	*SmKLS2*	Tps-e	AHJ59325
22	*Marrubium vulgare*	*MvEKS*	Tps-e	AIE77093
23	*Marrubium vulgare*	*MvELS*	Tps-e	AIE77094
24	*Salvia miltiorrhiza*	*SmKSL*	Tps-e	ABV08817
25	*Isodon rubescens*	*IrSKL5*	Tps-e	ASC55317
26	*Isodon rubescens*	*IrSKL6*	Tps-e	ASC55318
27	*Plectranthus barbatus*	*CfTPS3*	Tps-e	AHW04048
28	*Isodon rubescens*	*IrSKL1*	Tps-e	APJ36376

29	*Isodon rubescens*	*IrTPS2*	Tps-e	ARO38140
30	*Isodon rubescens*	*IrCPS2*	Tps-c	APJ36372
31	*Salvia sclarea*	*SsLPPS*	Tps-c	AFU61897
32	*Salvia miltiorrhiza*	*SmCPS1*	Tps-c	AHJ59321
33	*Salvia miltiorrhiza*	*SmCPS2*	Tps-c	AHJ59322
34	*Salvia miltiorrhiza*	*SmCPS5*	Tps-c	AHJ59324
35	*Marrubium vulgare*	*MvCPS1*	Tps-c	AIE77090
36	*Salvia miltiorrhiza*	*SmCPS4*	Tps-c	AKN91186
37	*Salvia divinorum*	*SdCPS2*	Tps-c	APH81400
38	*Plectranthus barbatus*	*CfTPS2*	Tps-c	AHW04047
39	*Plectranthus barbatus*	*CfTPS1*	Tps-c	AHW04046
40	*Vitex agnus castus*	*VacTPS1*	Tps-c	AUT77120
41	*Vitex agnus castus*	*VacTPS3*	Tps-c	AUT77122
42	*Vitex agnus castus*	*VacTPS4*	Tps-e	AUT77123
43	*Vitex agnus castus*	*VacTPS5*	Tps-c	AUT77124
44	*Ajuga reptans*	*ArTPS3*	Tps-e	AZB50367
45	*Origanum majorana*	*OmTPS5*	Tps-e	AZB50373
46	*Salvia officinalis*	*SoTPS1*	Tps-e	AZB50376
47	*Ajuga reptans*	*ArTPS1*	Tps-c	AZB50377
48	*Ajuga reptans*	*ArTPS2*	Tps-c	AZB50378
49	*Hyptis sauveolans*	*HsTPS1*	Tps-c	AZB50380
50	*Leonotis leonorus*	*LlTPS1*	Tps-c	AZB50381
51	*Nepata mussinii*	*NmTPS1*	Tps-c	AZB50382
52	*Pogostemon cablin*	*PCTPS1*	Tps-c	AZB50385

Table S4

Click here to access/download
Supplementary Material
TableS4.xlsx
