# Supplementary material for: Generation of a chromosome-scale genome assembly of the insect-repellent terpenoid-producing Lamiaceae species, Callicarpa americana
Source: Gigascience. 2020 Sep 7;9(9):giaa093. doi: 10.1093/gigascience/giaa093 (PMC7476102; doi:10.1093/gigascience/giaa093)
Supplement: giaa093_GIGA-D-20-00049_Revision_1 [file giaa093_giga-d-20-00049_revision_1.pdf]

GigaScience

Generation of a chromosome-scale genome assembly of the insect-repellent terpenoid-producing Lamiaceae species, Callicarpa americana

--Manuscript Draft--

**Manuscript Number:**	GIGA-D-20-00049R1
**Full Title:**	Generation of a chromosome-scale genome assembly of the insect-repellent terpenoid-producing Lamiaceae species, Callicarpa americana
**Article Type:**	Data Note
**Funding Information:**	Directorate for Biological Sciences (IOS-1444499) — Prof. C Robin Buell<br>US Department of Agriculture (MICL02431) — Prof. C Robin Buell<br>US Department of Agriculture (MICL02454) — Dr Bjoern Hamberger<br>US Department of Energy (DE-FC02-07ER64494) — Dr Bjoern Hamberger<br>US Department of Energy (DE-SC0018409) — Dr Bjoern Hamberger<br>Michigan State University Strategic Partnership Grant (None) — Dr Bjoern Hamberger, Prof. C Robin Buell
**Abstract:**	Background: Plants exhibit wide chemical diversity due to the production of specialized metabolites which function as pollinator attractants, defensive compounds, and signaling molecules. Lamiaceae (mints) are known for their chemodiversity and have been cultivated for use as culinary herbs as well as sources of insect repellents, health-promoting compounds, and fragrance. Findings: We report the chromosome-scale genome assembly of Callicarpa americana L. (American beautyberry), a species within the early-diverging Callicarpoideae clade of Lamiaceae, known for its metallic purple fruits and use as an insect repellent due to its production of terpenoids. Using long read sequencing and Hi-C scaffolding, we generated a 506.1 Mb assembly spanning 17 pseudomolecules with N50 contig and N50 scaffold sizes of 7.5 Mb and 29.0 Mb, respectively. In all, 32,164 genes were annotated, including 53 candidate terpene synthases and 47 putative clusters of specialized metabolite biosynthetic pathways. Our analyses revealed three putative whole-genome duplication events, which together with local tandem duplications, contributed to gene family expansion of terpene synthases. Kolavenyl diphosphate is a gateway to many of the bioactive terpenoids in C. americana; experimental validation confirmed that CamTPS2 encodes kolavenyl diphosphate synthase. Syntenic analyses with Tectona grandis L. f. (teak), a member of the Tectonoideae clade of Lamiaceae known for exceptionally strong wood resistant to insects, revealed 963 collinear blocks and 21,297 C. americana syntelogs. Conclusions: Access to the C. americana genome provides a roadmap for rapid discovery of genes encoding plant-derived agrichemicals and a key resource for understanding the evolution of chemical diversity in Lamiaceae.
**Corresponding Author:**	C Robin Buell<br>Michigan State University<br>East Lansing, Michigan UNITED STATES
**Corresponding Author Secondary Information:**	
**Corresponding Author's Institution:**	Michigan State University
**Corresponding Author's Secondary Institution:**	
**First Author:**	John P Hamilton
**First Author Secondary Information:**	
**Order of Authors:**	John P Hamilton

Powered by Editorial Manager® and ProduXion Manager® from Aries Systems Corporation

	Grant Godden
	Emily Lanier
	Wajid W Bhat
	Taliesin Kinser
	Brieanne Vaillancourt
	Haiyan Wang
	Joshua Wood
	Jiming Jiang
	Pamela S Soltis
	Douglas E Soltis
	Bjoern Hamberger
	C Robin Buell
**Order of Authors Secondary Information:**	
**Response to Reviewers:**	Dear Editor,<br>We have revised our manuscript and uploaded a point-by-point response to reviewers concerns as well as a marked-up version of our manuscript (both available as Supplementary Files). We feel that we have addressed the reviewer's concerns and that our manuscript is now suitable for publication in GigaScience.<br>Sincerely,<br>C. Robin Buell
**Additional Information:**	
**Question**	**Response**
Are you submitting this manuscript to a special series or article collection?	No
**Experimental design and statistics**<br><br>Full details of the experimental design and statistical methods used should be given in the Methods section, as detailed in our Minimum Standards Reporting Checklist. Information essential to interpreting the data presented should be made available in the figure legends.<br><br>Have you included all the information requested in your manuscript?	Yes
**Resources**<br><br>A description of all resources used, including antibodies, cell lines, animals and software tools, with enough information to allow them to be uniquely identified, should be included in the	Yes

Methods section. Authors are strongly encouraged to cite Research Resource Identifiers (RRIDs) for antibodies, model organisms and tools, where possible.<br><br>Have you included the information requested as detailed in our Minimum Standards Reporting Checklist?	
**Availability of data and materials**<br><br>All datasets and code on which the conclusions of the paper rely must be either included in your submission or deposited in publicly available repositories (where available and ethically appropriate), referencing such data using a unique identifier in the references and in the “Availability of Data and Materials” section of your manuscript.<br><br>Have you have met the above requirement as detailed in our Minimum Standards Reporting Checklist?	Yes

Powered by Editorial Manager® and ProduXion Manager® from Aries Systems Corporation

Manuscript
Click here to access/download;Manuscript;Callicarpa_Ms_v13.docx

DATA NOTE

Generation of a chromosome-scale genome assembly of the insect-repellent terpenoid-producing Lamiaceae species, *Callicarpa americana*

John P. Hamilton[1], Grant T. Godden[2], Emily Lanier[3], Wajid Waheed Bhat[3], Taliesin J. Kinser[2,4], Brieanne Vaillancourt[1], Haiyan Wang[1], Joshua C. Wood[1], Jiming Jiang[1,5,6], Pamela S. Soltis[2], Douglas E. Soltis[2,4], Bjoern Hamberger[3,6], and C. Robin Buell[1,6,7]

[1]Department of Plant Biology, Michigan State University, East Lansing, MI 48824, USA
[2]Florida Museum of Natural History, University of Florida, Gainesville, FL 32611, USA
[3]Department of Biochemistry & Molecular Biology, Michigan State University, East Lansing, MI 48824, USA
[5]Department of Horticulture, Michigan State University, East Lansing MI 48824 USA
[6]MSU AgBioResearch, Michigan State University, East Lansing MI 48824 USA
[7]Plant Resilience Institute, Michigan State University, East Lansing MI 48824 USA

∗Correspondence address. C. Robin Buell, Department of Plant Biology, Michigan State University, 612 Wilson Road, East Lansing, MI 48824, USA, E-mail: buell@msu.edu, +1-517-353-5597

Keywords: beautyberry, callicarpenal, clerodane, gene cluster, insect repellent, kolavenyl diphosphate, specialized metabolites, terpene synthase

ORCIDs:
John P. Hamilton, 0000-0002-8682-5526;
Grant T. Godden, 0000-0003-2628-4729;
Emily Lanier, 0000-0002-1878-4566;
Wajid Waheed Bhat, 0000-0002-7049-8671;
Taliesin J. Kinser, 0000-0002-9497-5399;
Brieanne Vaillancourt, 0000-0002-6795-5173;
Joshua C. Wood, 0000-0002-7691-6088;
Jiming Jiang, 0000-0002-6435-6140;
Pamela S. Soltis, 0000-0001-9310-8659;
Douglas E. Soltis, 0000-0001-8638-4137;
Bjoern Hamberger, 0000-0003-1249-1807;
C. Robin Buell, 0000-0002-6727-4677

Abstract

Background: Plants exhibit wide chemical diversity due to the production of specialized metabolites which function as pollinator attractants, defensive compounds, and signaling molecules. Lamiaceae (mints) are known for their chemodiversity and have been cultivated for use as culinary herbs as well as sources of insect repellents, health-promoting compounds, and fragrance. *Findings:* We report the chromosome-scale genome assembly of *Callicarpa americana* L. (American beautyberry), a species within the early-diverging Callicarpoideae clade of Lamiaceae, known for its metallic purple fruits and use as an insect repellent due to its production of terpenoids. Using long read sequencing and Hi-C scaffolding, we generated a 506.1 Mb assembly spanning 17 pseudomolecules with N50 contig and N50 scaffold sizes of 7.5 Mb and 29.0 Mb, respectively. In all, 32,164 genes were annotated, including 53 candidate terpene synthases and 47 putative clusters of specialized metabolite biosynthetic pathways. Our analyses revealed three putative whole-genome duplication events, which together with local tandem duplications, contributed to gene family expansion of terpene synthases. Kolavenyl diphosphate is a gateway to many of the bioactive terpenoids in *C. americana*;

experimental validation confirmed that *CamTPS2* encodes kolavenyl diphosphate synthase. Syntenic analyses with *Tectona grandis* L. f. (teak), a member of the Tectonoideae clade of Lamiaceae known for exceptionally strong wood resistant to insects, revealed 963 collinear blocks and 21,297 *C. americana* syntelogs. *Conclusions:* Access to the *C. americana* genome provides a roadmap for rapid discovery of genes encoding plant-derived agrichemicals and a key resource for understanding the evolution of chemical diversity in Lamiaceae.

Data Description

Introduction

Mints (Lamiaceae) are the sixth largest family of flowering plants and include many species grown for use as culinary herbs (basil, rosemary, thyme), food additives and flavorings (peppermint, spearmint), pharmaceuticals and health-promoting activities (skullcap, bee balm), feline euphoria induction (catnip), wood (teak), fragrance (lavender, patchouli), insect repellents (peppermint, rosemary), and ornamentals (coleus, chaste tree, beautyberry). This diverse set of uses for Lamiaceae is due in part to their production of specialized metabolites, primarily terpenes (monoterpenes, sesquiterpenes, diterpenes) and iridoids (irregular terpenes). Through an integrated phylogenetic-genomic-chemical approach, the evolutionary basis of Lamiaceae chemical diversity was shown to involve gene family expansion, differential gene expression, diversion of metabolic flux, and parallel evolution [1]. Genome sequences are currently available for a number of Lamiaceae species and are providing new insights into these phenomena, yet are primarily limited to members of Nepetoideae [2-5], the most species- and monoterpene-rich of the 12 major mint clades (= traditional subfamilies). As for the remaining major clades, a genome sequence is available only for *Tectona grandis* L. f. (teak; Tectonoideae) [6]. To expand our knowledge of the genome evolution underlying chemodiversity in this important family, we generated a chromosome-scale assembly of *Callicarpa americana* L. (American beautyberry, NCBI:txid204211), a species renowned for its charismatic purple fruits (Figure 1A). *Callicarpa* occupies a pivotal phylogenetic position as a representative from the early-diverging mint lineage, Callicarpoideae [1]. The species is native to North America (southern U. S. A., northern Mexico), North Atlantic (Bermuda, Bahamas), and Cuba, and has known insect repellent activity [7, 8] due to production of spathulenol, intermedeol, and callicarpenal [9]. Access to its genome will enable discovery of the genes encoding the biosynthetic pathways for these terpenes and the potential for heterologous expression of botanical-derived insect repellents; the genome is also an important evolutionary reference for the mint family.

Plant material, DNA and RNA extraction, library preparation, and sequencing

Leaf tissue from a greenhouse-cultivated accession of *C. americana* (voucher: N. García 4530 [FLAS]) was harvested and frozen in liquid nitrogen. High-molecular-weight DNA for Pacific Biosciences (PacBio) libraries was extracted using a modified cetyl trimethylammonium bromide (CTAB) method ((2% cetyl trimethylammonium bromide (CTAB), 100 mM Tris, 1.4 M sodium chloride, 20 mM EDTA, 1% 2-Mercaptoethanol) [10] and treated with RNase A. Large (>15 kb) insert libraries were constructed using the Pacific Biosciences SMRTbell Template prep kit 1.0-SPV3 and sequenced on 11 PacBio Sequel SMRT Cells (Pacific Biosciences, Menlo Park, CA) at the University of Georgia Genomics and Bioinformatics Core. DNA was extracted from young leaf tissue using a modified CTAB method (2% cetyl trimethylammonium bromide (CTAB), 100 mM Tris, 1.4 M sodium chloride, 20 mM EDTA, 1% 2-Mercaptoethanol, 2% Polyvinylpyrrolidone (PVP)) method [10] and treated with RNase A. An Illumina-compatible 250-bp size selected genomic paired-end library was constructed for use in error correction. Sequencing was performed on an Illumina HiSeq 4000 (RRID:SCR_016386； Illumina, San Diego, CA) in paired-end mode generating 150 nt reads. A proximity ligation (Hi-C) library was constructed from *C. americana* leaf tissue as described previously [11, 12] and sequenced on an Illumina HiSeq 4000. For transcriptome analyses, RNA was isolated from mature and young leaves, stems, petioles, roots, flowers (open and closed), and ripened whole fruits (denoted by the deep purple color) from growth-chamber-grown plants using a hot phenol method [13]. Illumina TruSeq Stranded mRNA (polyA mRNA) libraries were constructed and sequenced on an Illumina HiSeq 4000 to 150 nt in paired-end mode. All Illumina sequencing was performed at the Research Technology Support Facility at Michigan State University.

Genome assembly

The average flow cytometry genome size estimate of *C. americana* was 538 Mb, and we assembled the genome using 45 Gb (81x coverage) PacBio reads ($\geq$ 1 kb) using Canu (v1.7; RRID:SCR_015880; [14]; Tables S1, S2) with the parameters minReadLength=1000 genomeSize=530m. The Canu assembly was polished with two rounds of Arrow (v2.2.2; [15]) using alignments of the PacBio reads generated with pbalign(v0.3.1; [16]). Final polishing was

then performed with Pilon (v1.22; RRID:SCR_014731; [17]) using whole-genome shotgun Illumina reads that were trimmed using Cutadapt (v1.15; RRID:SCR_011841; [18]) with the parameters -n 2 -m 100 -q 10 and aligned to the assembly with BWA-MEM (v0.7.17; RRID:SCR_010910; [19]). The polished Canu contigs (965 total) had an N50 of 7,510,543 bp totaling 506,106,333 bp (Table 1), consistent with the estimated genome size. A chromosome count was performed using root tips as described previously [20], revealing 34 chromosomes (Figure 1B); as *C. americana* is diploid, this represents a haploid chromosome number of 17. The Canu contigs were then scaffolded into 17 pseudochromosomes using the Hi-C reads (Table S1) and the Phase Genomics Proximo Hi-C genome scaffolding platform as described in Jibran et al. [21]. The final assembly has an N50 scaffold size of 29,054,287 bp representing 506,362,408 bp on 328 scaffolds; 493,744,786 bp are contained within the 17 pseudochromosomes leaving 311 scaffolds representing 12,617,622 bp unanchored (Table 1).

To assess the genic representation in the final assembly, RNA-sequencing (RNA-seq) reads from eight libraries (Table S1) were processed using Cutadapt (v1.15; -n 2 -m 100 -q 10; [18]) to trim adapters and remove low-quality sequence. Cleaned RNA-seq reads were aligned to the genome using HiSAT2 (v2.1.0; RRID:SCR_015530; [22]) with the parameters: --max-intronlen 5000 --rna-strandness RF, revealing an average alignment percentage of 96.03% (Table S3). Analysis using Benchmarking Universal Single-Copy Orthologs (v3.0.2; RRID:SCR_015008; [23]) with the Embryophyta v9 database revealed 93.8% complete orthologs (1,351), of which, 1,241 (86.2%) were single copy and 110 (7.6%) were duplicated; 1.3% of the orthologs were fragmented (19), and 4.9% (70) were missing. Collectively, these data demonstrate a high-quality assembly of the *C. americana* genome.

To estimate the heterozygosity of the genome, canonical k-mers (k=21) from the Illumina WGS reads were counted using Jellyfish2 (v2.2.9; RRID:SCR_005491; [24]). The kmer count histogram was analyzed by the online version of GenomeScope (RRID:SCR_017014) [25, 26] and the heterozygosity of the genome was estimated at 0.158% (Figure S1).

Genome annotation

To annotate the genome, we generated a species-specific custom repeat library using RepeatModeler (v1.0.8; RRID:SCR_015027 ; [27]). Protein-coding genes were removed using ProtExcluder (v1.1; [28]), and Viridiplantae repeats from RepBase [29] were used to create a final custom repeat library that was used to mask the genome. Repeat-masked versions of the genome were generated using RepeatMasker (v4.0.6; RRID:SCR_012954; [30]; -s -nolow -no_is -gff); in total, 55.9% of the genome was masked. Genome-guided transcripts were assembled from the HISAT2 (v2.1.0; RRID:SCR_015530; [31]; --max-intronlen 5000 --rna-strandness RF) alignments of each RNA-seq library using Trinity (v2.6.6; RRID:SCR_013048; --SS_lib_type RF --min_contig_length 500 --genome_guided_max_intron 5000 --genome_guided_bam; Table S3; [32]). To train AUGUSTUS, genome-guided RNA-seq alignments from the young leaf library were used as evidence; initial gene predictions were made on the hard-masked assembly. Gene models were improved using PASA2 (v2.1.0; RRID:SCR_014656; [33, 34]) and the individual library genome-guided transcript assemblies as transcript evidence. Two rounds of annotation comparison were performed to generate the working gene model set which comprised 36,480 genes (loci) encoding 67,826 gene models (Table 2).

High-confidence gene models were identified using protein domain and gene expression abundance. Working gene models were searched against PFAM (v32; RRID:SCR_004726; [35]) with hmmscan (HMMER v3.1b2; RRID:SCR_00530) with a cutoff of --domE 1e-3 -E 1e-5. Gene expression values (transcripts per million (TPM; Table S4)) for the working gene model set were generated using Kallisto (v0.45.0; RRID:SCR_016582; [36]) and cleaned RNA-seq reads from each library. Gene models were identified as high confidence if they had a TPM value > 0 in at least one RNA-seq library and/or had a PFAM domain match. Partial gene models and models with matches to transposable element-related PFAM domains were excluded from the high-confidence model set. Functional annotation was assigned by first searching the gene model predicted proteins against the *Arabidopsis* proteome (TAIR10; RRID:SCR_004618; [37]), the PFAM database (v32; RRID:SCR_004726; [38]), and Swiss-Prot plant proteins (release 2015_08; RRID:SCR_004426). The search results were processed in the same order and the function of

the first hit encountered was assigned to the gene model. The final high-confidence gene set contained 32,164 loci encoding 62,993 gene models (Table 2).

Comparative genome analyses

Callicarpa is the only genus of Callicarpoideae, with ~170 species. In addition to being the first species of *Callicarpa* with a genome sequence, the *C. americana* genome is useful for comparative studies because of its phylogenetic position within an early-diverging mint lineage. To better understand orthologous relationships within Lamiaceae, we used Orthofinder (v2.3.7; RRID:SCR_017118; [39]) with six angiosperm species: *Callicarpa americana* (this study), *Amborella trichopoda* Baill [40] (*Amborella*), *Oryza sativa* L. (Rice, MSU v7; [41]), *Arabidopsis thaliana* (L.) Heynh (Araport 11; [42]), and two Lamiaceae species: *Tectona grandis* (teak, Tectonoideae; [6]) and *Salvia splendens* Ker Gawl. (scarlet sage; Nepetoideae [4]) (Figure 2) to define orthologous and paralogous clusters. A total of 9,026 orthologous groups contained at least one protein from each of the six species (Figure 2A) [1]. *Tectona grandis* (Tectonoideae), *S. splendens* (Nepetoideae), and *C. americana* (Callicarpoideae) represent three major subclades of Lamiaceae; the OrthoFinder analysis identified 1,247 orthogroups that were unique to Lamiaceae. Gene ontology (GO) terms were assigned to the *C. americana* predicted proteome by searching the representative gene models against the Interpro databases using InterProScan (v5.34.73.0; RRID:SCR_005829; [43]). TopGO (v2.36.0; RRID:SCR_014798; [44]). Analysis of Lamiaceae-specific genes revealed numerous biological process terms associated with response to stress (Table S5), including defense response (GO:0006952), response to wounding (GO:0009611), and innate immunity (GO:0045087). Species of Lamiaceae are well known for their chemical diversity [1], and Lamiaceae-specific orthologous groups were enriched in molecular function terms including oxidoreductase activity (GO:0016705; GO:0016702), catechol oxidase activity (GO:0004097), and transferase activity (GO:0004097; GO:0016758) (Table S5).

Synteny analyses between *T. grandis* and *C. americana* were performed with MCScanX (git commit 7b61f32; [45, 46]) to identify inter-species collinear blocks. We identified 963

collinear blocks, representing 456 Mb of unique *C. americana* sequence; 31,235 *C. americana* genes were present in the collinear blocks, of which, 21,297 were syntelogs with *T. grandis* (Figure 2B). Ancient whole-genome duplication (WGD) events were inferred from estimates of divergence at synonymous sites (K_S) among paralogous gene pairs present in the *C. americana* genome and compared with previous transcriptome-based inferences [47]. Coding sequences representing the longest isoform of each gene were filtered from the high-confidence gene set and analyzed with DupPipe using default settings [48]. Following an analysis workflow used previously with Lamiaceae [6, 47], significant peaks in the observed K_S distribution were identified with Gaussian mixture models, as implemented in the mixtools R package [49], and corroborated with results from a SiZer analysis [50]. Four components were predicted by the mixture models (Table S6; Figure 3), although only mean values at K_S = 0.12, 0.47, 1.74 were supported as significant data features by SiZer results, providing evidence for three ancient WGD events in *C. americana*. Of these putative WGDs, events placed at K_S = 0.12 and K_S = 1.74 were not previously detected or supported by transcriptome-based analyses, highlighting the benefits of WGD inferences from genomic data (discussed in [47]). We found no evidence for a shared ancient WGD based on K_S results for *C. americana* and *T. grandis* using genomic data. Only one putative ancient WGD event (K_S = 0.60) was detected in *T. grandis* [6, 47], and available chromosome counts (i.e., 2*n* = 16 or 18 in *Callicarpa vs.* 2*n* = 36 in *Tectona* [51]) suggest that *Tectona* has experienced at least one unique WGD event following its divergence from its common ancestor with *C. americana*. Moreover, results from recent phylotranscriptomic analyses [47] are most consistent with independent WGDs in (i) the common ancestor of *Callicarpa*, *Westringia*, and *Prostanthera* and (ii) the common ancestor of all remaining Lamiaceae, indicating that the WGDs of *Callicarpa* and *Tectona* are not shared. In contrast, one analysis of transcriptomic data shows a single WGD in the ancestor of Lamiaceae, suggesting that *Callicarpa* and *Tectona* share an ancestral WGD [47]. However, the genome-based K_S results do not show this pattern, and the overall results indicate independent WGDs in these two early branches of mint phylogeny.

Specialized metabolite analyses

Callicarpa americana produces a range of bioactive diterpenoids derived from the C_{20} clerodane skeleton [52], a less common instance of the labdanoid diterpenes. These include the C_{16} nor-diterpenoid (-)-callicarpenal with a range of mosquito, tick, and arthropod repellent activities [8]. Clerodane-type diterpenes are derived from the precursor kolavenyl diphosphate (KPP), which is formed by class II diterpene synthases (diTPS) of the terpene synthase c (TPS-c) subfamily [53-55]. Here, we describe the annotation and validation of the KPP synthase in *C. americana*, a gateway to many of its bioactive terpenoids. Using the assembled genomic and transcriptomic data, we performed a sequence similarity search with BLASTP comparing the *C. americana* peptide models against a set of reference TPSs (Supplemental Text). Peptides shorter than 350 amino acids or having less than 30% identity to the most similar reference sequence were filtered out, yielding a total of 53 candidate TPSs (Table S7). We used phylogenetic clustering (Figure 4; Supplemental Text) with known TPSs to identify and classify candidates most likely to catalyze the formation of KPP. The complement, and distribution of TPSs discovered was found in accordance with plant species [56], reflecting general metabolism and species-specific evolution of specialized metabolism in *C. americana*. Specifically, our study resulted in eight putative diTPSs from the TPS-c subfamily; class II diTPSs are typically involved in formation of the necessary diphosphate intermediates of the labdane-type chemistry. Of the eight candidates, four were successfully cloned from cDNA and transferred into the plant expression vector pEAQ [6] as described previously. The others were not further pursued due to low expression levels or a lack of expression in tissues relevant for callicarpenal formation. Expression analysis of tissue-specific accumulation of transcripts for the diTPSs (Figure 5) showed the highest expression in young leaves and flowers for *CamTPS2*, consistent with the presence of callicarpenal in leaves. Characterization of the candidates through transient expression in *Nicotiana benthamiana*, and GC/MS analysis as described previously ([57]; Supplemental Text), showed that CamTPS1 and CamTPS3 catalyze the formation of *ent*-copalyl diphosphate (Figure 6), the first step in the biosynthesis of the ubiquitous *ent*-kaurane type plant growth hormone gibberellic acid (GA) and specialized metabolites in the *ent*-configuration found in this genus. CamTPS6 yielded (+)-copalyl diphosphate, precursor of calliterpenone, a rare (+)-kaurane type diterpene found across several species of *Callicarpa* [52]. (+)-Copalyl

diphosphate is also the intermediate to the common diterpene miltiradiene, precursor to many defense related diterpenoids found in other Lamiaceae and previously identified in other *Callicarpa* species [52]. Finally, CamTPS2 was confirmed to yield the possible precursor of callicarpenal, KPP (Figure 6). All products were confirmed by comparison with reference combinations of diTPS. Specifically, diTPS yielded access to *ent*-copalyl diphosphate (*ent*-CPP, CamTPS1 and Cam TPS3, Figure S2A), CPP in normal configuration ((+)-CPP, CamTPS6, Figure S2B) and kolavenyl diphosphate (KPP, CamTPS2, Figure S2C), all plausible precursors to the known chemical diversity of diterpene scaffolds in *C. americana*.

Genes encoding some specialized metabolic pathways are found physically clustered in plant genomes [58, 59]. We utilized the PlantiSMASH analytical pipeline [60] to identify physically clustered specialized metabolic pathway genes (Table 3). The most frequent type of cluster encoded saccharides (15), terpenes (9), uncharacterized clusters (8), and alkaloids (5). Several clusters of *C. americana* TPSs indicate significant expansion of the family by local tandem duplications (Figure 4). Consistent with earlier findings in *S. miltiorrhizza* and *T. grandis*, where the genes involved in miltiradiene biosynthesis were found clustered [2, 6], *CamTPS6* was identified as part of a large cluster of putative terpene biosynthetic genes, including *CamTPS9,* the gene encoding the subsequently acting class I enzyme CamTPS9. The cluster also carries several genes encoding cytochromes P450 of relevant subfamilies of the CYP71 clan, the largest repository for enzymes involved in terpene functionalization [61].

Conclusion

The insect repellent activity of *C. americana* is due to the production of the terpenoids spathulenol, intermedeol, and callicarpenal [9], and access to a chromosome-scale genome assembly of *C. americana* permitted identification of kolavenyl diphosphate synthase which synthesizes kolavenyl diphosphate, a precursor to callicarpenal. As the sixth largest angiosperm family, and with extensive chemical diversity, Lamiaceae are an ideal group for application of phylogenomic data-mining, a powerful approach for biosynthetic pathway discovery. Generation of the genome of *C. americana*, of the early-diverging Callicarpoideae clade of

Lamiaceae, provides a roadmap for rapid discovery of genes encoding plant-derived agrichemicals and a key resource for understanding the evolution of both chemical diversity and mint genomes.

Availability of supporting data

All sequences generated in this study are available in the NCBI SRA under BioProject PRJNA529675. The genome assembly, annotation files, expression matrix, and other supporting data can be accessed at the *GigaScience* GigaDB database [62]. Genbank accession identifiers for cloned TPSs are: MT083919- MT083922. Original raw GC-MS data were deposited to Zenodo [63] and Metabolights [64] under accession MTBLS1983.

Additional files

Table S1: RNA-Seq, whole-genome shotgun, and Hi-C libraries used in this study.

Table S2. PacBio flow cells used in this study.

Table S3. *Callicarpa americana* RNA-seq alignment and genome-guided assembly transcript metrics.

Table S4. Expression abundances of *Callicarpa americana* genes.

Table S5. Gene ontology enrichment analyses of Lamiaceae-specific genes.

Table S6. Gaussian mixture modeling and SiZer results for the K_S distribution estimated from the genome and transcriptome of *Callicarpa americana* L. Shown here are the number of inferred components, along with their corresponding means ($\boldsymbol{\mu}$), mixing proportions ($\boldsymbol{\lambda}$), and standard deviations ($\boldsymbol{\sigma}$) estimated by mixtools. The number of components corroborated by a SiZer analysis is indicated in brackets, with corresponding values of $\boldsymbol{\mu}$, $\boldsymbol{\lambda}$, and $\boldsymbol{\sigma}$ from mixture models denoted with an asterisk (*). Transcriptome-based results from Godden et al. [47].

Table S7. Terpene synthases identified in this study.

Table S8. GenBank protein identifiers of the TPSs used for construction of phylogenetic tree.

Table S9. Manually curated phylogeny for terpene synthases.

Figure S1. Estimated heterozygosity of *Callicarpa americana* L. as revealed by GenomeScope [25].

Figure S2. GC/MS data for TPS enzymes investigated alongside reference diTPS enzymes. Each class II diTPS is paired with a characterized class I diTPS and elution time/mass spectra compared to a pair of reference diTPS. A, CamTPS1 and CamTPS3 paired with NmTPS2 produce *ent*-kaurene, thus confirming that CamTPS1 and CamTPS3 both produce *ent*-CPP. The reference pair of ZmAn2 + NmTPS2 makes *ent*-kaurene from *ent*-CPP. B, CamTPS6 paired with CfTPS3 makes miltiradiene, confirming activity as a (+)-CPP synthase. The reference pair NmTPS1 + CfTPS3 makes miltiradiene from (+)-CPP. C, CamTPS2 paired with ScSS makes kolavelool, confirming CamTPS2 as a KPP synthase. The reference pair ShTPS1 + ScSS makes kolavelool from KPP. Reference enzymes NmTPS1, NmTPS2 *Nepeta mussini*; CfTPS3, *Coleus forskohlii*; ZmAN2, *Zea mays*; SsSCS, *Salvia sclarea* [55, 57, 65-67].

Abbreviations

BLAST: Basic Local Alignment Search Tool; BUSCO: Benchmarking Universal Single-Copy Orthologs; CRL: Custom Repeat Library; KPP: kolavenyl diphosphate; NCBI: National Center for Biotechnology Information; RNA-seq: RNA-sequencing; SRA: Sequence Read Archive; TPM: transcripts per million; TPS: Terpene synthase

Competing interests

The authors declare no competing interests.

Funding

Funds for this study were provided by a grant to C.R.B., D.S., and P.S. from the National Science Foundation Plant Genome Research Program (IOS-1444499), a grant to C.R.B. and Bj.H. from the Michigan State University Strategic Partnership Grants Program, and from Hatch funds to C.R.B. (MICL02431). Bj.H. gratefully acknowledges the U.S. Department of Energy-Great Lakes Bioenergy Research Center Cooperative Agreement DE-FC02-07ER64494 and DE-SC0018409, the Michigan State University Strategic Partnership Grant program “Plant-inspired Chemical Diversity”, startup funding from the Department of Molecular Biology and Biochemistry, Michigan State University, and support from Michigan State University AgBioResearch (MICL02454).

Author contributions

JPH performed the genome assembly, annotation, and comparative analyses. BV and JW isolated nucleic acids and performed quality assessments. HW and JJ performed the chromosome counting. GG and TJK performed the whole-genome duplication analyses. BH, ERL, DS, PS, and CRB designed the experiments. WWB performed the phylogenetic analyses and built the CamTPS repository. ERL identified and functionally characterized the terpene synthases. CRB, JH, GG, and BH wrote the manuscript. All authors approved the final manuscript.

Acknowledgements

None

Figure Legends

Figure 1. A. *Callicarpa americana* L. (beautyberry) plant with fruit. B. Somatic chromosome squash of a root tip cell of *C. americana* with $2n = 34$. Bar = 10 µm.

Figure 2. Comparative genome analyses with *Callicarpa americana* L. A. Upset plot showing orthologous groups between *C. americana* and five other angiosperms (*Amborella trichopoda* Baill [40] (*Amborella*), *Oryza sativa* L. (Rice, MSU v7), *Arabidopsis thaliana* (L.) Heynh (Araport 11;[42]), and two Lamiaceae species: *Tectona grandis* (teak, Tectonoideae; [6]) and *Salvia splendens* Ker Gawl. (scarlet sage; Nepetoideae [4])). Only the 30 largest intersections are shown. B. Syntenic relationship between *T. grandis* (teak) and *C. americana* (beautyberry). The upper row shows the 17 *C. americana* pseudomolecules with syntenic alignments to the 19 *T. grandis* pseudomolecules.

Figure 3. Whole-genome duplication (WGD) events inferred from the *Callicarpa americana* L. (beautyberry) genome. Gaussian distributions produced by mixture models in the mixtools R package [49] are shown as overlays on the K_S distribution, with red or blue color-coded peaks representing putative WGD events that were either corroborated or not corroborated (i.e., false positives), respectively, by results from SiZer analysis ([50]; lower plot). The SiZer plot shows significant increases (blue) or decreases (red), or no significant changes (pink) across the K_S distribution at various (log transformed) bandwidths to distinguish true data features from noise.

Figure 4. Phylogenetic analysis and classification of the *Callicarpa americana* terpene synthase family [6]. Shown are the distinct terpene synthase gene families TPS-a to TPS-g. Highlighted in boxes are TPSs clustered in proximity on the genomic pseudomolecules. *C. americana* TPSs in bold; red stars, functionally characterized members of the TPS-c subfamily; dots on branches indicate bootstrap support equal to or greater than 80%. The phylogeny was rooted with the bifunctional *Physcomitrella patens* (moss) PpCPS/EKS. Annotation of *C. americana* and reference TPSs are given in Tables S7 and S8.

Figure 5. Tissue-specific expression of the *Callicarpa americana* L. terpene synthase gene family. Expression is in transcripts per million. TPS subfamily classification of *C. americ*ana TPSs is given in Table S8. Red stars, functionally characterized members of the TPS-c subfamily.

Figure 6. Activities of functionally characterized *Callicarpa americana* L. TPS-c. Dotted arrows indicate putative further functionalization by class I diTPS and cytochromes P450 to diterpene products accumulating in *C. americana*.

Tables

Table 1. Metrics of final *Callicarpa americana* L. genome assembly.

Feature	Metric
Canu-derived Contigs	
N50 contig Size (bp)	7,510,543
NG50 contig Size (bp)	6,369,058
L50 contig count	25
LG50 contig count	27
Total Assembly Size (bp)	506,106,333
Number of Contigs	965
Maximum Contig Length (bp)	18,804,173
Minimum Contig Length (bp)	1,028
Hi-C Scaffolded Assembly	
N50 Scaffold Size (bp)	29,054,287
NG50 Scaffold Size (bp)	28,692,425
L50 Scaffold count	8
LG50 Scaffold count	9
Total Assembly Size (bp)	506,362,408
Number of Scaffolds	328
Maximum Scaffold Length (bp)	39,429,362
Minimum Scaffold Length (bp)	1,028
Number of Pseudomolecules	17
Total Pseudomolecule Size (bp)	493,744,786
Number of Unanchored Scaffolds	311
Total Unanchored Scaffolds Size (bp)	12,617,622
Pseudomolecules	
Chr01 (bp)	39,429,362
Chr02 (bp)	32,953,817
Chr03 (bp)	32,428,638
Chr04 (bp)	32,381,817
Chr05 (bp)	31,681,419
Chr06 (bp)	31,029,626
Chr07 (bp)	29,370,463
Chr08 (bp)	29,054,287
Chr09 (bp)	28,692,425
Chr10 (bp)	28,677,202
Chr11 (bp)	28,224,296
Chr12 (bp)	27,270,263
Chr13 (bp)	27,197,714
Chr14 (bp)	27,108,606
Chr15 (bp)	23,772,120

Chr16 (bp)	22,946,943
Chr17 (bp)	21,525,788

Table 2. *Callicarpa americana* L. gene annotation summary

	Working Model Set	High-Confidence Model Set
Number of Gene Models	67,826	62,993
Number of Loci	36,480	32,164
Maximum Transcript Length (bp)	16,862	15,978
Maximum CDS Length (bp)	16,269	15,294
Average Transcript Length (bp)	2,004.6	2,096.2
Average CDS Length (bp)	1,305.6	1,355.2
Average Exon Length (bp)	323.5	323.8
Average Intron Length (bp)	500.4	497.4
Single Exon Transcripts	18,140	14,496

Table 3. Physically clustered specialized metabolite biosynthetic pathways in *Callicarpa americana* L. as identified by PlantiSMASH.

Type	Number
Alkaloid	5
Lignan	2
Lignan-Saccharide	1
Polyketide	3
Saccharide	15
Saccharide-Polyketide	1
Saccharide-Terpene	2
Terpene	9
Terpene-Polyketide	1
Uncharacterized	8
Total	47

Supplementary methods

Phylogenetic tree

C. americana TPSs were identified by Blastp (v. 2.2.31+) [68] using a set of reference terpene synthases across all TPS-subfamilies against the gene models. Hits with less than 350 amino acids or less than 30% identity to the reference sequences were filtered out. Reference sequences for functionally characterized TPS are given in Table S8. Sequences were aligned using the MUSCLE program from MEGA [69], using default parameters and the alignment was manually verified for consistency (Table S9). A maximum likelihood tree was generated using Jones-Taylor-Thornton model with MEGA X [69] with 1,000 bootstrap repetitions. The tree figure was generated using FigTree v1.4.3 [70].

Heatmap generation

Gene expression heat maps were generated by using ClustVis web tool [71] with the default routine, using TPM values of the TPS gene expression in different tissues of *C. americana* (Table S4).

diTPS cloning

From RNA (extracted as detailed in the main text), cDNA was prepared using the Invitrogen SuperScript™ IV One-Step RT-PCR System. After cloning into pJET1.2 (Thermo Fisher Scientific, Waltham, MA, USA), TPSs were transferred into pEAQ-HT [72] using In-Fusion® HD Cloning Plus (Takara Bio, California, USA) for transient expression in *Nicotiana benthamiana*.
Oligonucleotides for cloning of *C. americana* TPS candidates (given in 5' to 3'):

Cam_TPS1_For	AAGCTCTCCTCTGCCGTTAAA
Cam_TPS1_Rev	CACAACTTTCATGTACATACTATACC
Cam_TPS2_For	ATGTCATTTGCTTCCCATGCCA
Cam_TPS2_Rev	CAGAACAGGAAGTGTAACTCTACC
Cam_TPS3_For	TCCAATCACACCAACGTTAATTTC
Cam_TPS3_Rev	GATTTACATGTACGTACATGGTCAGAG
Cam_TPS6_For	CTTTGCTACACTGCAGACAAC

Cam_TPS6_Rev AGTTCGACCGAATTGCGGAAACA

Functional characterization of diTPSs by transient expression in *N. benthamiana*

DiTPS candidates and reference genes were transiently expressed in *N. benthamiana* leaves as previously described in detail [57]. In brief, to increase product accumulation, diTPSs were co-expressed with genes from the upstream pathway providing the substrate, CfDXS and CfGGPPS (Cf, *Coleus forskohlii*) [65, 66]. Cultures containing different constructs were mixed in equal ratios to yield the appropriate combinations before infiltration into 4-5 weeks old plants. Plants were grown for an additional five days before metabolite extraction. Leaf discs of 2 cm diameter (approximately 0.1 g fresh weight) were cut from the infiltrated leaves. Diterpenes were extracted in 1 mL n-hexane with 1 mg/L 1-eicosene as internal standard (IS) at room temperature overnight in an orbital shaker at 200 rpm. Plant material was collected by centrifugation and the organic phase transferred to GC vials for analysis.

GC-MS analyses were performed on an Agilent 7890A GC with an Agilent VF-5ms column (30 m x 250 µm x 0.25 µm, with 10m EZ-Guard) and an Agilent 5975C detector. The inlet was set to 275°C splitless injection, He carrier gas with column flow of 1 mL/min. The oven program was 40°C hold 1 min, 40 °C/min to 200°C and hold 4.5 min, 20°C/min to 240°C, 10°C/min to 280°C, 40°C/min to 320°C hold 3 min. The detector was activated after a four-minute solvent delay. All analyses were done in duplicate. Original raw GC-MS data were deposited to Zenodo [63]

References

1. Mint Evolutionary Genomics Consortium. Phylogenomic Mining of the Mints Reveals Multiple Mechanisms Contributing to the Evolution of Chemical Diversity in Lamiaceae. Mol Plant. 2018;11 8:1084-96. doi:10.1016/j.molp.2018.06.002.
2. Xu H, Song J, Luo H, Zhang Y, Li Q, Zhu Y, et al. Analysis of the genome sequence of the medicinal plant *Salvia miltiorrhiza*. Mol Plant. 2016;9 6:949-52. doi:10.1016/j.molp.2016.03.010.
3. Malli RPN, Adal AM, Sarker LS, Liang P and Mahmoud SS. *De novo* sequencing of the *Lavandula angustifolia* genome reveals highly duplicated and optimized features for essential oil production. Planta. 2019;249 1:251-6. doi:10.1007/s00425-018-3012-9.
4. Dong AX, Xin HB, Li ZJ, Liu H, Sun YQ, Nie S, et al. High-quality assembly of the reference genome for scarlet sage, *Salvia splendens*, an economically important ornamental plant. Gigascience. 2018;7 7 doi:10.1093/gigascience/giy068.
5. Zhao Q, Yang J, Cui MY, Liu J, Fang Y, Yan M, et al. The Reference Genome Sequence of *Scutellaria baicalensis* Provides Insights into the Evolution of Wogonin Biosynthesis. Mol Plant. 2019;12 7:935-50. doi:10.1016/j.molp.2019.04.002.
6. Zhao D, Hamilton JP, Bhat WW, Johnson SR, Godden GT, Kinser TJ, et al. A chromosomal-scale genome assembly of *Tectona grandis* reveals the importance of tandem gene duplication and enables discovery of genes in natural product biosynthetic pathways. Gigascience. 2019;8(3):giz005. doi:10.1093/gigascience/giz005.
7. Krajick K. Medical entomology. Keeping the bugs at bay. Science. 2006;313 5783:36-8. doi:10.1126/science.313.5783.36.
8. Cantrell CL, Klun, J.A. Callicarpenal and Intermedeol: Two natural arthropod feeding deterrent and repellent compounds Iidentified from the southern folk remedy plant, *Callicarpa americana*. Recent Developments in Invertrebrate Repellents. Washington DC: American Chemical Society; 2011.
9. Cantrell CL, Klun JA, Bryson CT, Kobaisy M and Duke SO. Isolation and identification of mosquito bite deterrent terpenoids from leaves of American (*Callicarpa americana*) and Japanese (*Callicarpa japonica*) beautyberry. J Agric Food Chem. 2005;53 15:5948-53. doi:10.1021/jf0509308.
10. Doyle JJ, Doyle, J.L. . A rapid DNA isolation procedure for small quantities of fresh leaf tissue. Phytochemical Bulletin. 1987;19:11-5.
11. Bickhart DM, Rosen BD, Koren S, Sayre BL, Hastie AR, Chan S, et al. Single-molecule sequencing and chromatin conformation capture enable de novo reference assembly of the domestic goat genome. Nat Genet. 2017;49 4:643-50. doi:10.1038/ng.3802.
12. Burton JN, Adey A, Patwardhan RP, Qiu R, Kitzman JO and Shendure J. Chromosome-scale scaffolding of de novo genome assemblies based on chromatin interactions. Nat Biotechnol. 2013;31 12:1119-25. doi:10.1038/nbt.2727.

13. Davidson RM, Gowda M, Moghe G, Lin H, Vaillancourt B, Shiu SH, et al. Comparative transcriptomics of three Poaceae species reveals patterns of gene expression evolution. Plant Journal. 2012;71 3:492-502. doi:10.1111/j.1365-313X.2012.05005.x.
14. Koren S, Walenz BP, Berlin K, Miller JR, Bergman NH and Phillippy AM. Canu: scalable and accurate long-read assembly via adaptive k-mer weighting and repeat separation. Genome Res. 2017;27 5:722-36. doi:10.1101/gr.215087.116.
15. PacBio® variant and consensus caller. https://github.com/PacificBiosciences/GenomicConsensus. September 2019.
16. PacBio pbalign GitHub. https://github.com/PacificBiosciences/pbalign Accessed May 2018.
17. Walker BJ, Abeel T, Shea T, Priest M, Abouelliel A, Sakthikumar S, et al. Pilon: an integrated tool for comprehensive microbial variant detection and genome assembly improvement. PLoS One. 2014;9 11:e112963. doi:10.1371/journal.pone.0112963.
18. Martin M. Cutadapt removes adapter sequences from high-throughput sequencing reads. EMBnetjournal. 2011;17 1 doi:http://dx.doi.org/10.14806/ej.17.1.200.
19. Li H. Aligning sequence reads, clone sequences and assembly contigs with BWA-MEM. arXiv. 2013;1303.3997v2.
20. Braz GT, He L, Zhao H, Zhang T, Semrau K, Rouillard JM, et al. Comparative oligo-FISH Mapping: An Efficient and Powerful Methodology to Reveal Karyotypic and Chromosomal Evolution. Genetics. 2018;208:513-23. doi:10.1534/genetics.117.300344.
21. Jibran R, Dzierzon H, Bassil N, Bushakra JM, Edger PP, Sullivan S, et al. Chromosome-scale scaffolding of the black raspberry (*Rubus occidentalis* L.) genome based on chromatin interaction data. Hortic Res. 2018;5:8. doi:10.1038/s41438-017-0013-y.
22. Kim D, Langmead B and Salzberg SL. HISAT: a fast spliced aligner with low memory requirements. Nature Methods. 2015;12 4:357-60. doi:10.1038/nmeth.3317.
23. Simao FA, Waterhouse RM, Ioannidis P, Kriventseva EV and Zdobnov EM. BUSCO: assessing genome assembly and annotation completeness with single-copy orthologs. Bioinformatics. 2015;31 19:3210-2. doi:10.1093/bioinformatics/btv351.
24. Marcais G and Kingsford C. A fast, lock-free approach for efficient parallel counting of occurrences of k-mers. Bioinformatics. 2011;27 6:764-70. doi:10.1093/bioinformatics/btr011.
25. Vurture GW, Sedlazeck FJ, Nattestad M, Underwood CJ, Fang H, Gurtowski J, et al. GenomeScope: fast reference-free genome profiling from short reads. Bioinformatics. 2017;33 14:2202-4. doi:10.1093/bioinformatics/btx153.
26. GenomeScope Software. http://qb.cshl.edu/genomescope/ Accessed April 2020.
27. Smit A, Hubley, R.: RepeatModler. http://www.repeatmasker.org/.
28. Campbell MS, Law M, Holt C, Stein JC, Moghe GD, Hufnagel DE, et al. MAKER-P: a tool kit for the rapid creation, management, and quality control of plant genome annotations. Plant Physiol. 2014;164 2:513-24. doi:10.1104/pp.113.230144.
29. Jurka J, Kapitonov VV, Pavlicek A, Klonowski P, Kohany O and Walichiewicz J. Repbase Update, a database of eukaryotic repetitive elements. Cytogenet Genome Res. 2005;110 1-4:462-7.
30. Chen N. Using RepeatMasker to identify repetitive elements in genomic sequences. Curr Protoc Bioinformatics. 2004;Chapter 4:Unit 4 10.

31. Kim D, Paggi JM, Park C, Bennett C and Salzberg SL. Graph-based genome alignment and genotyping with HISAT2 and HISAT-genotype. Nat Biotechnol. 2019;37 8:907-15. doi:10.1038/s41587-019-0201-4.
32. Grabherr MG, Haas BJ, Yassour M, Levin JZ, Thompson DA, Amit I, et al. Full-length transcriptome assembly from RNA-Seq data without a reference genome. Nature Biotechnology. 2011;29 7:644-52. doi:10.1038/nbt.1883.
33. Haas BJ, Delcher AL, Mount SM, Wortman JR, Smith RK, Jr., Hannick LI, et al. Improving the Arabidopsis genome annotation using maximal transcript alignment assemblies. Nucleic Acids Res. 2003;31 19:5654-66.
34. PASA2. http://pasapipeline.github.io/. Accessed 26 March 2017.
35. Campbell MA, Haas BJ, Hamilton JP, Mount SM and Buell CR. Comprehensive analysis of alternative splicing in rice and comparative analyses with Arabidopsis. BMC Genomics. 2006;7:327.
36. Bray NL, Pimentel H, Melsted P and Pachter L. Near-optimal probabilistic RNA-seq quantification. Nat Biotechnol. 2016;34 5:525-7. doi:10.1038/nbt.3519.
37. The Arabidopsis Information Resource. Arabidopsis.org.
38. Finn RD, Coggill P, Eberhardt RY, Eddy SR, Mistry J, Mitchell AL, et al. The Pfam protein families database: towards a more sustainable future. Nucleic Acids Res. 2016;44 D1:D279-85. doi:10.1093/nar/gkv1344.
39. Emms DM and Kelly S. OrthoFinder: phylogenetic orthology inference for comparative genomics. Genome Biol. 2019;20 1:238. doi:10.1186/s13059-019-1832-y.
40. Amborella Genome Project. The Amborella genome and the evolution of flowering plants. Science. 2013;342 6165:1241089. doi:10.1126/science.1241089.
41. Kawahara Y, de la Bastide M, Hamilton JP, Kanamori H, McCombie WR, Ouyang S, et al. Improvement of the *Oryza sativa* Nipponbare reference genome using next generation sequence and optical map data. Rice. 2013;6 4 doi:doi:10.1186/1939-8433-6-4.
42. Cheng C-Y, Krishnakumar V, Chan AP, Thibaud-Nissen F, Schobel S and Town CD. Araport11: a complete reannotation of the Arabidopsis thaliana reference genome. The Plant Journal. 2017;89 4:789-804. doi:10.1111/tpj.13415.
43. Jones P, Binns D, Chang H-Y, Fraser M, Li W, McAnulla C, et al. InterProScan 5: genome-scale protein function classification. Bioinformatics. 2014;30 9:1236-40. doi:10.1093/bioinformatics/btu031.
44. Alexa A, Rahnenfuhrer, J. topGO: Enrichment Analysis for Gene Ontology. R package version 2.38.1. 2019.
45. Wang Y, Tang H, Debarry JD, Tan X, Li J, Wang X, et al. MCScanX: a toolkit for detection and evolutionary analysis of gene synteny and collinearity. Nucleic Acids Res. 2012;40 7:e49. doi:10.1093/nar/gkr1293.
46. MCScanX GitHub. https://github.com/wyp1125/MCScanX Accessed Jan 2019.
47. Godden GT, Kinser TJ, Soltis PS and Soltis DE. Phylotranscriptomic Analyses Reveal Asymmetrical Gene Duplication Dynamics and Signatures of Ancient Polyploidy in Mints. Genome Biol Evol. 2019;11 12:3393-408. doi:10.1093/gbe/evz239.
48. Barker MS, Dlugosch KM, Dinh L, Challa RS, Kane NC, King MG, et al. EvoPipes.net: Bioinformatic Tools for Ecological and Evolutionary Genomics. Evol Bioinform Online. 2010;6:143-9. doi:10.4137/EBO.S5861.

49. Benaglia T CD, Hunter DR, Young, D.S. mixtools : An R Package for Analyzing Finite Mixture Models. J Stat Softw. 2009;32:1-29.
50. Chaudhuri P, Marron, J. S. SiZer for Exploration of Structures in Curves. J Am Stat Assoc 1999;94:807.
51. Harley RM. Labiatae. In: JW K, editor. The families and genera of vascular plants: flowering plants—dicotyledons. Berlin: Springer-Verlag; 2004. p. 167–275.
52. Jones WP and Kinghorn AD. Biologically active natural products of the genus *Callicarpa*. *Current bioactive compounds*. 2008, p. 15-32.
53. Hansen NL, Heskes AM, Hamberger B, Olsen CE, Hallström BM, Andersen-Ranberg J, et al. The terpene synthase gene family in *Tripterygium wilfordii* harbors a labdane-type diterpene synthase among the monoterpene synthase TPS-b subfamily. The Plant Journal. 2017;89:429-41. doi:10.1111/tpj.13410.
54. Chen X, Berim A, Dayan FE and Gang DR. A (–)-kolavenyl diphosphate synthase catalyzes the first step of salvinorin A biosynthesis in S*alvia divinorum*. *Journal of Experimental Botany*. 2017, p. 1109-22.
55. Pelot KA, Mitchell R, Kwon M, Hagelthorn DM, Wardman JF, Chiang A, et al. Biosynthesis of the psychotropic plant diterpene salvinorin A: Discovery and characterization of the *Salvia divinorum* clerodienyl diphosphate synthase. The Plant Journal. 2017;89:885-97. doi:10.1111/tpj.13427.
56. Jiang SY, Jin J, Sarojam R and Ramachandran S. A Comprehensive Survey on the Terpene Synthase Gene Family Provides New Insight into Its Evolutionary Patterns. Genome Biol Evol. 2019;11 8:2078-98. doi:10.1093/gbe/evz142.
57. Johnson SR, Bhat WW, Bibik J, Turmo A, Hamberger B, Mint Evolutionary Genomics Consortium, et al. A database-driven approach identifies additional diterpene synthase activities in the mint family (Lamiaceae). J Biol Chem. 2018;25:1349-62. doi:10.1074/jbc.RA118.006025.
58. Nutzmann HW, Huang A and Osbourn A. Plant metabolic clusters - from genetics to genomics. New Phytol. 2016;211 3:771-89. doi:10.1111/nph.13981.
59. Liu Z, Suarez Duran HG, Harnvanichvech Y, Stephenson MJ, Schranz ME, Nelson D, et al. Drivers of metabolic diversification: how dynamic genomic neighbourhoods generate new biosynthetic pathways in the Brassicaceae. New Phytol. 2019; doi:10.1111/nph.16338.
60. Kautsar SA, Suarez Duran HG, Blin K, Osbourn A and Medema MH. plantiSMASH: automated identification, annotation and expression analysis of plant biosynthetic gene clusters. Nucleic Acids Res. 2017;45 W1:W55-W63. doi:10.1093/nar/gkx305.
61. Hamberger B and Bak S. Plant P450s as versatile drivers for evolution of species-specific chemical diversity. *Philosophical Transactions of the Royal Society B: Biological Sciences*. 2013.
62. Hamilton JP, Godden, G.T., Lanier, E., Bhat, W.W., Kinser, T.J. Vaillancourt, B., and Wang H, Wood, J.C., Jiang, J., Soltis, P.S., Soltis, D.E., Hamberger, B., Buell, C.R. (2020) Supporting data for "Generation of a chromosome-scale genome assembly of the insect-repellent terpenoid-producing Lamiaceae species, *Callicarpa americana*". *GigaScience* Database. http://dx.doi.org/10.5524/100777.

63. Hamilton JP, Godden, G.T., Lanier, E., et al. (2020). GC-MS data set for Generation of a chromosome-scale genome assembly of the insect-repellant terpenoid-producing Lamiaceae species, Callicarpa americana. Zenodo. http://doi.org/10.5281/zenodo.3672159
64. MetaboLights. https://www.ebi.ac.uk/metabolights/. Accessed August 2020.
65. Andersen-Ranberg J, Kongstad KT, Nielsen MT, Jensen NB, Pateraki I, Bach SS, et al. Expanding the landscape of diterpene structural diversity through stereochemically controlled combinatorial biosynthesis. Angewandte Chemie - International Edition. 2016;55 6:2142-6. doi:10.1002/anie.201510650.
66. Pateraki I, Andersen-Ranberg J, Hamberger B, Heskes AM, Martens HJ, Zerbe P, et al. Manoyl oxide (13R), the biosynthetic precursor of forskolin, is synthesized in specialized root cork cells in *Coleus forskohlii*. Plant Physiology. 2014;164 3:1222-36. doi:10.1104/pp.113.228429.
67. Harris LJ, Saparno A, Johnston A, Prisic S, Xu M, Allard S, et al. The Maize An2 Gene is Induced by Fusarium Attack and encodes an *ent*-Copalyl Diphosphate Synthase. Plant Molecular Biology. 2005;59:881-94. doi:10.1007/s11103-005-1674-8.
68. Camacho C, Coulouris G, Avagyan V, Ma N, Papadopoulos J, Bealer K, et al. BLAST+: architecture and applications. BMC Bioinformatics. 2009;10:421. doi:10.1186/1471-2105-10-421.
69. Kumar S, Stecher G and Tamura K. MEGA7: Molecular Evolutionary Genetics Analysis Version 7.0 for Bigger Datasets. Mol Biol Evol. 2016;33 7:1870-4. doi:10.1093/molbev/msw054.
70. Figtree: Figtree. http://tree.bio.ed.ac.uk/software/figtree/.2019.
71. ClustVis web tool: https://biit.cs.ut.ee/clustvis/. 2019.
72. Sainsbury F, Thuenemann EC and Lomonossoff GP. pEAQ: versatile expression vectors for easy and quick transient expression of heterologous proteins in plants. Plant Biotechnol J. 2009;7:682-93. doi:10.1111/j.1467-7652.2009.00434.x.

Click here to access/download
Supplementary Material
Response2ReviewresComments_v9.pdf

Marked up version of manuscript

Click here to access/download

Supplementary Material

MarkedUP.pdf

Table S1

Click here to access/download
Supplementary Material
TableS1.xlsx

Table S2

Click here to access/download
Supplementary Material
TableS2.xlsx

Table S3

Click here to access/download
Supplementary Material
Tables3.xlsx

Table S4

Click here to access/download
Supplementary Material
TableS4.xlsx

Table S5

Click here to access/download
Supplementary Material
TableS5.xlsx

Table S6

Click here to access/download
Supplementary Material
TableS6.xlsx

Table S7

Click here to access/download
Supplementary Material
TableS7.xlsx

Table S8

Click here to access/download
Supplementary Material
TableS8.xlsx

Table S9

Click here to access/download
Supplementary Material
TableS9.xlsx
