# Supplementary material for: Generation of a chromosome-scale genome assembly of the insect-repellent terpenoid-producing Lamiaceae species, Callicarpa americana
Source: Gigascience. 2020 Sep 7;9(9):giaa093. doi: 10.1093/gigascience/giaa093 (PMC7476102; doi:10.1093/gigascience/giaa093)
Supplement: giaa093_Supplemental_Files [file giaa093_supplemental_files.zip › SupplementalText1.docx]

**Supplementary text 1**

**Phylogenetic tree**

*C. americana* TPSs were identified by Blastp (v. 2.2.31+) [68] using a set of reference terpene synthases across all TPS-subfamilies against the gene models. Hits with less than 350 amino acids or less than 30% identity to the reference sequences were filtered out. Reference sequences for functionally characterized TPS are given in Table S8. Sequences were aligned using the MUSCLE program from MEGA [69], using default parameters and the alignment was manually verified for consistency (Table S9). A maximum likelihood tree was generated using Jones-Taylor-Thornton model with MEGA X [69] with 1,000 bootstrap repetitions. The tree figure was generated using FigTree v1.4.3 [70].

**Heatmap generation**

Gene expression heat maps were generated by using ClustVis web tool [71] with the default routine, using TPM values of the TPS gene expression in different tissues of *C. americana* (Table S4).

**diTPS cloning**

From RNA (extracted as detailed in the main text), cDNA was prepared using the Invitrogen SuperScript™ IV One-Step RT-PCR System. After cloning into pJET1.2 (Thermo Fisher Scientific, Waltham, MA, USA), TPSs were transferred into pEAQ-HT [72] using In-Fusion® HD Cloning Plus (Takara Bio, Mountain View, California, USA) for transient expression in *Nicotiana benthamiana*. Oligonucleotides for cloning of *C. americana* TPS candidates (given in 5’ to 3’):

Cam_TPS1_For AAGCTCTCCTCTGCCGTTAAA

Cam_TPS1_Rev CACAACTTTCATGTACATACTATACC

Cam_TPS2_For ATGTCATTTGCTTCCCATGCCA

Cam_TPS2_Rev CAGAACAGGAAGTGTAACTCTACC

Cam_TPS3_For TCCAATCACACCAACGTTAATTTC

Cam_TPS3_Rev GATTTACATGTACGTACATGGTCAGAG

Cam_TPS6_For CTTTGCTACACTGCAGACAAC

Cam_TPS6_Rev AGTTCGACCGAATTGCGGAAACA

**Functional characterization of diTPSs by transient expression in *N. benthamiana***

DiTPS candidates and reference genes were transiently expressed in *N. benthamiana* leaves as previously described in detail [57]. In brief, to increase product accumulation, diTPSs were co-expressed with genes from the upstream pathway providing the substrate, CfDXS and CfGGPPS (Cf, *Coleus forskohlii*) [65, 66]. Cultures containing different constructs were mixed in equal ratios to yield the appropriate combinations before infiltration into 4-5 weeks old plants. Plants were grown for an additional five days before metabolite extraction. Leaf discs of 2 cm diameter (approximately 0.1 g fresh weight) were cut from the infiltrated leaves. Diterpenes were extracted in 1 mL n-hexane with 1 mg/L 1-eicosene as internal standard (IS) at room temperature overnight in an orbital shaker at 200 rpm. Plant material was collected by centrifugation and the organic phase transferred to GC vials for analysis.

GC-MS analyses were performed on an Agilent 7890A GC with an Agilent VF-5ms column (30 m x 250 µm x 0.25 µm, with 10m EZ-Guard) and an Agilent 5975C detector. The inlet was set to 275°C splitless injection, He carrier gas with column flow of 1 mL/min. The oven program was 40°C hold 1 min, 40 °C/min to 200°C and hold 4.5 min, 20°C/min to 240°C, 10°C/min to 280°C, 40°C/min to 320°C hold 3 min. The detector was activated after a four-minute solvent delay. All analyses were done in duplicate. Original raw GC-MS data were deposited to Zenodo (doi.org/10.5281/zenodo.3672159).

**References**

68. Camacho C, Coulouris G, Avagyan V, et al. BLAST+: architecture and applications. BMC Bioinformatics 2009; 10:421.

69. Kumar S, Stecher G, Tamura K. MEGA7: molecular evolutionary genetics analysis version 7.0 for bigger datasets. Mol Biol Evol 2016;33(7):1870–4.

70. Figtree. http://tree.bio.ed.ac.uk/software/figtree/. Accessed January 2020.

71. ClustVis web tool. https://biit.cs.ut.ee/clustvis/. Accessed November 2019.

72. Sainsbury F, Thuenemann EC, Lomonossoff GP. pEAQ: versatile expression vectors for easy and quick transient expression of heterologous proteins in plants. Plant Biotechnol J 2009;7:682–93.
