# Supplementary material for: Generation of a chromosome-scale genome assembly of the insect-repellent terpenoid-producing Lamiaceae species, Callicarpa americana
Source: Gigascience. 2020 Sep 7;9(9):giaa093. doi: 10.1093/gigascience/giaa093 (PMC7476102; doi:10.1093/gigascience/giaa093)
Supplement: giaa093_Response_to_Reviewer_Comments_Original_Submission [file giaa093_response_to_reviewer_comments_original_submission.pdf]

Author's Response To Reviewer Comments

Close

Dear Editor,
We have revised our manuscript and uploaded a point-by-point response to reviewers concerns as well as a marked-up version of our manuscript (both available as Supplementary Files). We feel that we have addressed the reviewer's concerns and that our manuscript is now suitable for publication in GigaScience.
Sincerely,
C. Robin Buell

Close
