# Supplementary material for: Generation of a chromosome-scale genome assembly of the insect-repellent terpenoid-producing Lamiaceae species, Callicarpa americana
Source: Gigascience. 2020 Sep 7;9(9):giaa093. doi: 10.1093/gigascience/giaa093 (PMC7476102; doi:10.1093/gigascience/giaa093)
Supplement: giaa093_Reviewer_1_Report_Original_Submission — Niklas MÃ¤hler -- 3/25/2020 Reviewed [file giaa093_reviewer_1_report_original_submission.pdf]

Reviewer Report

Title: Generation of a chromosome-scale genome assembly of the insect-repellent terpenoid-producing Lamiaceae species, Callicarpa americana

Version: Original Submission **Date:** 3/25/2020

Reviewer name: Niklas MÃ¤hler

Reviewer Comments to Author:

The authors present a genome assembly of the mint species Callicarpa americana. In addition to the assembly itself, the authors also present a small comparative genomics study as well as a synteny analysis against Tectona grandis. Since mints are known for their secondary metabolite diversity, a section of the manuscript is dedicated to the identification and annotation of a kolavenyl diphosphate synthase.
The assembly seems to be of good quality and I think that this will be of great value to the scientific community. As far as I can tell, the choice of samples and tools seem sound. The manuscript reads well, and I don't have any major comments about the analysis or the manuscript itself. Below I list a few minor issues and questions that came to mind while going through the manuscript.
- I find that the methods are lacking some detail. All the software and their versions are listed, which is great, but the actual commandsâ€”or at least the non-default parametersâ€"that were used are in most cases left out. These are details that are easy to forget about, but here I'm taking the opportunity to remind the authors, as well as myself, to keep this in mind.
- To me it is not clear how the number of syntelogs were determined. I am not familiar with MCScanX, but from skimming the manual there is not a single mention of syntelogs, so I think that this could be expanded upon a bit more.
- In the section "Specialized metabolite analyses" I'm missing a more clear link between the candidate genes and the GC-MS data. Right now the authors refer to Figure 6, which shows the pathway in question annotated with the four candidate genes, but I would have liked to see more evidence from the GC-MS data that connect these genes with these particular steps. This might be related to my next point.
- Figure 7 (or at least the seventh figure) is never referred to, and there is no caption for it.
- Figure 1B: I would prefer to see the bar size in the figure itself instead of only in the caption.
- I would consider making figure 2A an UpSet plot (https://caleydo.org/tools/upset/) instead of a Venn diagram. That would make it much easier to associate the numbers to the different intersections.
- Figure 5: The unit for the heatmap is mentioned in the figure caption, but I would have liked to see it in the figure as well.
- Line 83 and 88: What was the modification made to the protocol?
- Line 111: 322 should probably be 311.
- Line 179: I assume that this is a commit ID, so in that case I would consider making that more clear.
- Table 1: I would consider adding the NG50 alongside the N50.
- Table S3: Should the percentage at the very bottom be aligned with the "HISAT2 Overall Alignment

Rate" instead of the "Cleaned Read Pairs" column?
- As a general comment for the tables, I usually find it easier to read them if column containing number are right-justified and text columns are left justified.
Best regards
//Niklas MÃ¤hler

Level of Interest

Please indicate how interesting you found the manuscript: Choose an item.

Quality of Written English

Please indicate the quality of language in the manuscript: Choose an item.

Declaration of Competing Interests

Please complete a declaration of competing interests, considering the following questions:

- Have you in the past five years received reimbursements, fees, funding, or salary from an organisation that may in any way gain or lose financially from the publication of this manuscript, either now or in the future?
- Do you hold any stocks or shares in an organisation that may in any way gain or lose financially from the publication of this manuscript, either now or in the future?
- Do you hold or are you currently applying for any patents relating to the content of the manuscript?
- Have you received reimbursements, fees, funding, or salary from an organization that holds or has applied for patents relating to the content of the manuscript?
- Do you have any other financial competing interests?
- Do you have any non-financial competing interests in relation to this paper?

If you can answer no to all of the above, write 'I declare that I have no competing interests' below. If your reply is yes to any, please give details below.

I declare that I have no competing interests.

I agree to the open peer review policy of the journal. I understand that my name will be included on my report to the authors and, if the manuscript is accepted for publication, my named report including any attachments I upload will be posted on the website along with the authors' responses. I agree for my report to be made available under an Open Access Creative Commons CC-BY license (http://creativecommons.org/licenses/by/4.0/). I understand that any comments which I do not wish to be included in my named report can be included as confidential comments to the editors, which will not be published.

Choose an item.

To further support our reviewers, we have joined with Publons, where you can gain additional credit to further highlight your hard work (see: https://publons.com/journal/530/gigascience). On publication of this paper, your review will be automatically added to Publons, you can then choose whether or not to claim your Publons credit. I understand this statement.

Yes Choose an item.
