# Supplementary material for: Generation of a chromosome-scale genome assembly of the insect-repellent terpenoid-producing Lamiaceae species, Callicarpa americana
Source: Gigascience. 2020 Sep 7;9(9):giaa093. doi: 10.1093/gigascience/giaa093 (PMC7476102; doi:10.1093/gigascience/giaa093)
Supplement: giaa093_Reviewer_2_Report_Original_Submission — Sunil Kumar Sahu, PhD., -- 3/31/2020 Reviewed [file giaa093_reviewer_2_report_original_submission.pdf]

Reviewer Report

Title: Generation of a chromosome-scale genome assembly of the insect-repellent terpenoid-producing Lamiaceae species, Callicarpa americana

Version: Original Submission **Date:** 3/31/2020

Reviewer name: Sunil Kumar Sahu, PhD.,

Reviewer Comments to Author:

Callicarpa americana, commonly called beautyberry, is a loose open shrub valued for its spectacular fruits. Being a member of Lamiaceae, they are also known for their rich chemodiversity. This work is of great interest, not only due to its biological property, but also due to its pivotal phylogenetic position as a representative from the early-diverging mint lineage. In the present study, by employing long read sequencing and Hi-C scaffolding, the authors report a high-quality chromosome scale genome assembly with N50 contig and N50 scaffold sizes of 7.5 Mb and 29.0 Mb, respectively. They focused on several candidate terpene synthases and putative clusters of specialized metabolite biosynthetic pathways. The dataset generated by the authors appears to be of good quality and I anticipate that it will be very useful for the research community. However, before accepting the manuscript in its current form, I have several concerns and would suggest some minor revisions as follows:

1. Assembly: The authors used Pacbio + HiC to generate a high-quality genome assembly. Although they described the details regarding Pacbio data assembly. I couldn't find much details regarding how the HiC scaffolding was performed (Line 107-109).
2. Although the genome assembly quality is pretty good, I was wondering why the duplicate number are higher (7.6%) [line 120-121]. I would also like to see the Heterozygosity ratio for this genome.
3. L191; the teak genome has at least one WGD event, while the C. americana has three WGD events, did they share any lineage-specific WGD event?
4. Annotation: Only the transcriptome data was used as an evidence to predict genes. I would suggest to add more evidences using homologue proteins using the tools like MAKER or the recent one BRAKER2.
5. In Figure 2B, I couldn't understand why a relatively distant species (Arabidopsis) was used for synteny analysis when more closely related genomes are available?
6. In Figure 3, Ks distribution: I doubt that the blue peak is correct. Therefore, I would suggest to use mixtools R package to validate the WGD peaks.
7. Most importantly, although it's a data note; the manuscript lacks description regarding the results with proper discussion. At least the results of figures should be described in a bit more detail.

Level of Interest

Please indicate how interesting you found the manuscript: Choose an item.

Quality of Written English

Please indicate the quality of language in the manuscript: Choose an item.

Declaration of Competing Interests

Please complete a declaration of competing interests, considering the following questions:

- Have you in the past five years received reimbursements, fees, funding, or salary from an organisation that may in any way gain or lose financially from the publication of this manuscript, either now or in the future?
- Do you hold any stocks or shares in an organisation that may in any way gain or lose financially from the publication of this manuscript, either now or in the future?
- Do you hold or are you currently applying for any patents relating to the content of the manuscript?
- Have you received reimbursements, fees, funding, or salary from an organization that holds or has applied for patents relating to the content of the manuscript?
- Do you have any other financial competing interests?
- Do you have any non-financial competing interests in relation to this paper?

If you can answer no to all of the above, write 'I declare that I have no competing interests' below. If your reply is yes to any, please give details below.

I declare that I have no competing interests

I agree to the open peer review policy of the journal. I understand that my name will be included on my report to the authors and, if the manuscript is accepted for publication, my named report including any attachments I upload will be posted on the website along with the authors' responses. I agree for my report to be made available under an Open Access Creative Commons CC-BY license (http://creativecommons.org/licenses/by/4.0/). I understand that any comments which I do not wish to be included in my named report can be included as confidential comments to the editors, which will not be published.

Choose an item.

To further support our reviewers, we have joined with Publons, where you can gain additional credit to further highlight your hard work (see: https://publons.com/journal/530/gigascience). On publication of this paper, your review will be automatically added to Publons, you can then choose whether or not to claim your Publons credit. I understand this statement.

Yes Choose an item.
